# Defining inkjet printing conditions of superconducting cuprate films through machine learning†

**DOI:** 10.1039/d1tc05913k

**Published:** 2022-04-07

**Authors:** Albert Queraltó, Adrià Pacheco, Nerea Jiménez, Susagna Ricart, Xavier Obradors, Teresa Puig

**Affiliations:** Institut de Ciència de Materials de Barcelona (ICMAB-CSIC) Campus UAB 08193 Bellaterra Catalonia Spain teresa.puig@icmab.es aqueralto@icmab.es +34 93 580 18 53

## Abstract

The design and optimization of new processing approaches for the development of rare earth cuprate (REBCO) high temperature superconductors is required to increase their cost-effective fabrication and promote market implementation. The exploration of a broad range of parameters enabled by these methods is the ideal scenario for a new set of high-throughput experimentation (HTE) and data-driven tools based on machine learning (ML) algorithms that are envisaged to speed up this optimization in a low-cost and efficient manner compatible with industrialization. In this work, we developed a data-driven methodology that allows us to analyze and optimize the inkjet printing (IJP) deposition process of REBCO precursor solutions. A dataset containing 231 samples was used to build ML models. Linear and tree-based (Random Forest, AdaBoost and Gradient Boosting) regression algorithms were compared, reaching performances above 87%. Model interpretation using Shapley Additive Explanations (SHAP) revealed the most important variables for each study. We could determine that to ensure homogeneous CSD films of 1 micron thickness without cracks after the pyrolysis, we need average drop volumes of 190–210 pl, and no. of drops between 5000 and 6000, delivering a total volume deposited close to 1 μl.

## Abstract 2

Machine learning provided helpful insights of the relation between the parameters that contribute to optimize the inkjet printing deposition of REBCO superconductors fabricated by the transient liquid assisted growth (TLAG-CSD) method.

## Graphical abstract

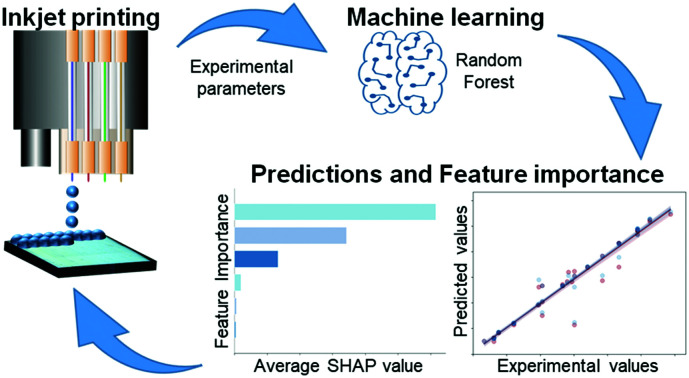

## 1. Introduction

The requirements to achieve a carbon neutral energy production demand the development of innovative technologies that enable the cost-effective generation and distribution of electricity from renewable energy sources. High-temperature superconductors (HTS) and particularly rare-earth cuprates (REBCO), discovered in 1986,^1^ have become the most important material type for HTS applications. The relatively high transition temperatures that allow HTS to operate using liquid nitrogen (77 K) but also at liquid helium (4.2 K), coupled with the outstanding properties exhibited in a wide range of magnetic fields and temperatures envisage a bright future for the superconductors’ industry. They have a wide market prospective implementation in sectors such as lightweight wind power generators for renewable electricity generation or its efficient transportation through coated conductor (CC) cables, but also in ultrahigh magnetic field applications where REBCO enable the generation of 20 T magnets required for confining the plasma in compact fusion reactors, or as low surface resistance coatings in high energy circular particle accelerators.^2–4^

However, the ceramic nature of REBCO and its high anisotropy require costly materials manufacturing processes and smart engineering solutions for their integration into functional devices. Specific architectures have been designed, known as coated conductors (CC).^5^ Although several companies are able to market CC,^6–12^ low-cost and robust fabrication methods that guarantee sufficiently small cost/performance ratios are essential and only few are available. Chemical solution deposition (CSD) methods have demonstrated low cost and fine capabilities in the fabrication of REBCO superconducting films. Recently, combining the cost effectiveness of environmentally-friendly precursor solutions with liquid assisted growth processes, newly developed transient-liquid assisted growth (TLAG-CSD) process was deployed.^13–18^ It allows reaching ultrafast growth rates above 100 nm s^−1^, as well as the versatility and scalability of deposition techniques such as inkjet printing or slot-die coating.

The complexity of the REBCO fabrication process based on the kinetically driven TLAG-CSD approach, involves multiple steps and a large number of experimental parameters that must be tuned simultaneously to reach a final optimized product. Several strategies are available to achieve such ambitious goal. The definitive screening design (DSD) is an approach that belongs to the Design of Experiments (DoE) methodology initially proposed by Ronald Fisher.^19^ It is based on selecting a small number of experimental parameters that contribute improving a target property and performing few tuning experiments using a factorial design, *i.e.* changing different parameters each time instead of tuning only one.^20–22^ The goal is to build conference matrices that are employed to construct models containing mainly, two-factor interactions and quadratic effects, and select the best ones using specific criteria such as Akaike's information criterion. DSD has been successfully applied to optimize the synthesis of mesoporous carbon, titania nanoparticles, as well as fine tune the fabrication of TFA-REBCO superconductors.^23–26^

On the other side of the spectrum we have the high-throughput approaches that explore a large number of parameters. High-throughput experimentation (HTE) is a methodology that is currently driving attention in fields such as catalysis,^27,28^ solar cells,^29,30^ batteries,^31^ electronics^32^ and superconductivity^33,34^ thanks to its capability for parallel sample fabrication and characterization. This expedites a swift generation of material databases containing a vast multiparameter space of compositions, processing conditions and properties. Specifically, we are using HTE for the exploration of a large variety of TLAG REBCO superconducting film parameters that go from solution stoichiometry variations to tuning of growth conditions.^17^ The novelty of the process together with the poor knowledge available due to its extremely non-equilibrium growth, makes HTE the most appropriate approach. In addition, the blossoming of a new data-driven paradigm thanks to the unceasing breakthroughs in artificial intelligence (AI) are promoting the development of high-throughput computational (HTC) tools, providing a platform to analyze the large amount of data generated from different sources in a timely manner that could be otherwise very time-consuming. In this sense, machine learning is a branch of AI that is contributing to accelerate materials development by identifying key elements in different steps of the fabrication process.^35–37^ Machine learning employs algorithms that receive input data to find hidden patterns and produce an output that is later used to drive the optimization of different aspects of materials development in varied research fields such as mechanical properties,^35,38^ photovoltaics,^39,40^ batteries^41,42^ and superconductivity.^43,44^

In this work, we propose our strategy for the development of machine learning models based on the high-throughput experimentation approach to optimize the CSD deposition to reach REBCO superconducting films. Specifically, we employ experimental data from the deposition of precursor solutions by drop-on-demand inkjet printing and use it to study the influence of different deposition parameters on the deposited film characteristics. We implement non-parametric tree-based machine learning algorithms which allow to capture non-linear relationships much better than multiple linear regression, albeit not outputting a model equation.

## 2. Methodology

### Experimental data acquisition

**Fig. 1:**
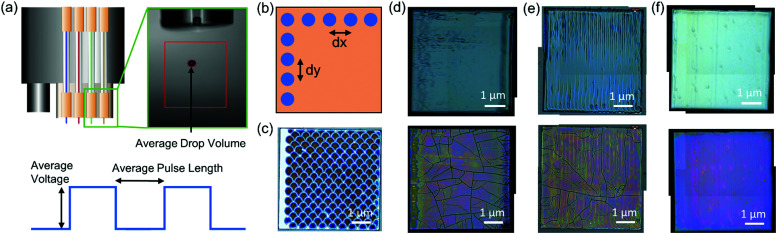
Schematic representation of (a) the inkjet printing setup, indicating the parameters to be tuned (voltage and pulse length) to obtain the average drop volume, and (b) the grid size showing the separation between drops, *i.e.*, drop pitch (d*x*) and line pitch (d*y*). (c) Optical microscopy (OM) image of a deposition with a 500 × 500 μm^2^ grid with an average drop volume (ADV) of 200 pl, a solution concentration of 0.75 M and 1.75% amine, (d) OM images of an inhomogeneous deposition (top) and associated pyrolysis (bottom) with a 50 × 100 μm^2^ grid, an ADV of 210 pl, a solution concentration of 1 M and 1.14% amine. (e) OM images of an inhomogeneous deposition (top) and associated pyrolysis (bottom) with a 50 × 100 μm^2^ grid, an ADV of 180 pl, a solution concentration of 1 M and 1.14% amine. (f) OM images of a homogeneous deposition (top) and pyrolysis (bottom) with a 50 × 95 μm^2^ grid, an ADV of 180 pl, a solution concentration of 1 M and 1.14% amine. The pyrolysis process was performed always with a O_2_ flux of 0.12 l min^−1^, at 5 °C min^−1^ up to 240 °C, and then at 3 °C min^−1^ up to 500 °C with a 5 min dwell.

Sample fabrication was done by drop-on-demand inkjet printing (IJP) of REBCO precursor solutions with a 1 M final concentration following the procedure described elsewhere.^17^ Briefly, the solutions were prepared by adding Y/Gd and Ba propionate precursors synthesized in-house, as well as commercial Cu(ac)_2_ (Merck KGaA) in the desired proportions into a 1-to-1 solvent mixture of propionic acid and butanol, as well as different quantities of a short C chain amine are added, boosting ink solubility and homogeneity, and also minimizing liquid movement during IJP. Additional details about solution preparation and amine composition cannot be disclosed due to confidentiality. Then, solutions were loaded in a multinozzle IJP system (Microdrop Technologies GmbH) and deposited on single-crystal (001) SrTiO_3_ (STO) substrates by using 1 or 2 nozzles (Fig. 1a). Successful IJP required adjusting drop formation and deposition parameters. The former consists of the voltage and pulse length at which the piezoelectric nozzles vibrate, enabling drop stabilization and control of its volume (Fig. 1a). The latter defines the grid size along *X* and *Y* directions which is comprised by the drop and line pitch (Fig. 1b). Finally, although we did not use this data in our models, IJP samples are heat-treated at 5 °C min^−1^ up to 240 °C and at 3 °C min^−1^ up to 500 °C for 5 min, in order to decompose the organic material and obtain nanocrystalline films (pyrolysis) that later will lead to the REBCO phase formation in a subsequent high-temperature heating step. The data of the parameters involved in the drop formation and deposition process that were used to build machine learning models was collected from 231 samples. Fig. 1c–f present some examples of homogeneous and inhomogeneous IJP depositions. Images were acquired with a Leica DM1750 M optical microscope. The experimental raw data for all samples used in the machine learning models was based on combinations of parameters that lead to uniform depositions, and is provided in the form of an Excel file at https://doi.org/10.20350/digitalCSIC/14016.

#### Variable definition

The dataset contains many experimental variables that depend on the number of nozzles used during deposition, either 1 or 2 nozzles. Feature simplification is typically performed in machine learning approaches to build the simplest possible models that brings a general comprehension of the underlying phenomena investigated. Thus, each one of the drop formation variables considered in our study has been averaged to combine the inputs for both nozzles. Below, we describe all the variables considered from the dataset to build the machine learning models:

• Average voltage, AV (V) is the voltage value obtained by averaging the voltages from nozzles 1 and 2. Each voltage is manually defined in the equipment software. Although it may vary depending on the nozzle used given the variability in their construction characteristics, we ensured that the nozzles used provided equivalent voltage values for our solutions.

• Average pulse length, APL (μs) is the average value between the pulse lengths from nozzles 1 and 2. Each pulse length is manually defined in the equipment software. Although it may vary depending on the nozzle used given the variability in their construction characteristics, we ensured that the nozzles used provided equivalent pulse lengths for our solutions.

• Amine (%) refers to the percentage of short C chain amine used in the preparation of the solution. The same amount of amine was added when two different REBCO precursor solutions were employed.

• Average drop volume, ADV (pl) is the average value between the drop volume from nozzles 1 and 2. The drop volume is obtained by adjusting the voltage and pulse length and inspecting the drop formation with a stroboscopic camera. It strongly depends on the solution rheology.

• Drop pitch, d*x* (μm) is defined as the spacing between drops in the *X* direction and it defines the grid size.

• Line pitch, d*y* (μm) is defined as the spacing between drops in the *Y* direction (lines) and it defines the grid size.

• No. of drops, NoD, one of the variables that we will model with machine learning, is the total number of drops deposited on a substrate that is calculated by considering the d*x* and d*y*, as well as the substrate width (*w*) and length (*l*) by using the following formula:1
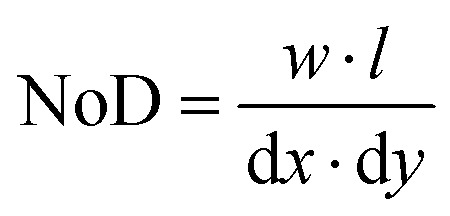


• Total volume deposited, TVD (μl), the other variable studied with machine learning, refers to the total amount of solution deposited on a substrate that it is calculated by multiplying the ADV and NoD:

2 TVD = ADV·NoD

#### Machine learning

Development and interpretation of machine learning models was done in Python using the Scikit-learn and SHAP libraries, respectively.^45,46^ The low dimensionality of our dataset (231 samples and 8 variables) restricted the type of algorithms that could be explored. Therefore, we used decision tree-based algorithms (ensemble methods) such as Random Forest (RF), AdaBoost (AB) and Gradient Boosting (GB) regressors which are also easy to implement and optimize in small datasets. A detailed description of the implementation and steps involved in our machine learning workflow, as well as additional details on the algorithms employed can be found in the ESI† (Section S1).

#### Code availability

The different steps of the machine learning model building process that is described in the ESI,† including preprocessing steps, figures, machine learning models and interpretability were done using Python. The code is freely available at https://doi.org/10.20350/digitalCSIC/14016.

## 3. Results and discussion

The deposition of uniform precursor REBCO precursor films by inkjet printing requires tuning and optimization of the experimental parameters involved in the solution (concentration, amine (%), viscosity, *etc.*), drop formation (AV, APL, and ADV), and deposition (d*x* and d*y*). These features will have a major influence in determining the no. of drops (NoD) and total volume deposited (TVD) on the substrates, which will impact on the deposition homogeneity, and liquid movement. While this imposes some constrains in the range of experimental data that could be used in our models, it ensures reaching high-quality depositions that will lead to crack-free and homogeneous pyrolyzed films, mandatory for subsequent studies at a later stage to investigate the REBCO epitaxial growth and superconducting properties. Hence, a study by machine learning of the hidden relations between these parameters, leading to uniform inkjet printing (IJP) depositions, will provide insights that contribute to understand and speed up the optimization of the deposition process as the initial step of the overall epitaxial REBCO film growth.

### 3.1. Feature correlation analysis

The construction of machine learning models is based on determining the importance of the different features on the target variables that we want to predict. In our case, we will build machine learning models for the NoD and TVD. Thus, it is necessary to previously explore all existing relationships for all variables in order to determine their importance and select which ones will be used in the models. This is typically done through the use of correlation matrices that represent the linear relationships between variables, two-by-two.

The coefficients (*ρ*) represented in the matrix range from −1 to 1, both extremes indicating a high correlation (negative or positive), while a value close to 0 indicates no correlation. Variables with values close to −1 or 1 are often used to discard one of the variables since they provide equivalent information.^47^ Nevertheless, since our dataset consists only of 231 samples and 8 variables, we will use all of them to study the existing relations. Detailed information about the theoretical aspects of correlation matrices and the statistical distribution of all variables in the dataset can be found in Sections S1 and S2 (ESI†).

**Fig. 2:**
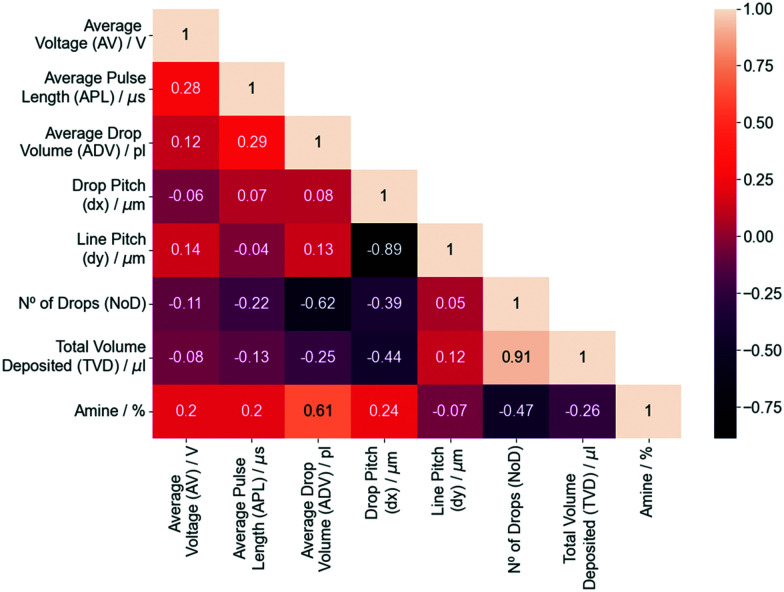
Correlation matrix showing the linear Pearson correlation coefficients (*ρ*) between all inkjet printing deposition variables.

Fig. 2 shows the correlation matrix for all the IJP variables considered in the dataset that will help us understand their relations before building machine learning models. As we mentioned previously, the ADV is experimentally determined by the combination of AV and APL during the operation of the IJP equipment. The correlation coefficients of the ADV with these variables are 0.12 and 0.29, respectively, indicating a very weak linear relationship from an optical perspective. In addition, the AV and APL have also a very low correlation between them (*ρ* = 0.28) which indicates that both variables should be kept in the modeling process. The amine displays also a considerably positive correlation (*ρ* = 0.61), indicating that higher ADVs are expected with more amine. However, such correlation may be also caused by the need to use higher voltages and pulse lengths with larger percentages of amine given the positive correlation between these variables and amine (*ρ* = 0.2). The need to raise the voltage and pulse length is likely caused by the increase in the viscosity of the solutions when more amine is incorporated to the solutions.^17^ Hence, we will initially also maintain these variables in our models.

The d*x* and d*y* are two variables contributing significantly to define uniform IJP depositions, especially because the sample set intends to define the best parameters in that homogeneous range to obtain films that eventually lead to crack-free pyrolysis of approximately 1 micron in thickness. Therefore, the d*x* and d*y* are linked due to these experimental requirements, following an inversely proportional relation with a high negative correlation between both variables (*ρ* = −0.89). We could have chosen other combinations of d*x* and d*y*, but it would have not provided useful information on the understanding and optimization of the IJP process. Hence, this implies one feature must decrease while the other increases since the requirements to reach high quality IJP depositions depend on how much volume is being ejected on the substrate that will eventually lead to our targeted thickness (around 1 micron after the pyrolysis step from the data used in our study).

The NoD is one of the target variables that we will model, and it is calculated from d*x* and d*y*. The correlation coefficients with the NoD and these variables are respectively −0.39 and 0.05. This would suggest that the NoD will diminish with d*x*, while there seems to be no correlation with d*y* despite increasing it should experimentally reduce the NoD given how it is calculated (eqn (1)). This relation seems illogical since one would expect that a larger d*y* leads to a lower NoD. However, the correlation matrix is only describing the linear relations between variables two-by-two and excludes any other interactions such as multicollinearity or non-linear behaviors which could lead to the observed correlation. Additionally, it could also hint that, in view of our data, d*x* has a greater effect in defining the NoD than d*y*. We can also identify additional relationships between the NoD and drop formation variables. The correlation coefficients are −0.11, −0.22, −0.47 and −0.62 for the AV, APL, Amine and ADV, pointing out that less drops will be deposited for large values of these variables. These results are expected since large ADVs are obtained by increasing the AV and APL. Furthermore, bigger drops will demand more space between them and a lower number to homogeneously cover the substrate surface for the targeted thickness.

The TVD is also a variable of interest that we will model using machine learning given its contribution to define the final pyrolyzed film quality and thickness (Fig. 1), and it is calculated from the NoD and the ADV (eqn (2)). Analyzing the correlation coefficients, the TVD has a *ρ* = 0.91 with the NoD which implies an almost linear relation between them and may indicate a large contribution of the NoD when we build the model. The correlation coefficient with the ADV is −0.25, which suggests a negative linear relationship between the two variables. Initially, this may seem counterintuitive since one would expect that a larger ADV leads to an also larger TVD but again, this analysis does not consider non-linear relationships. Besides that, the other drop formation features (AV, APL, and amine) also have negative and rather low coefficients (−0.08, −0.13 and −0.26) which entails a weak linear relation with the TVD. This could be expected since the AV, APL, and Amine are positively correlated with the ADV and their coefficient with the TVD should have the same sign. If we look at the relationships between the d*x* and d*y* with the TVD, we see that the correlation coefficients are respectively, −0.44, and 0.12. This implies that an increase in d*x* will contribute reducing the TVD which is in agreement with what one would expect if the distance between drops is enlarged. However, the positive coefficient for the d*y* suggests that the opposite trend occurs when the d*y* increases. This would disagree with our intuition, but one must also contemplate that d*x* and d*y* are inversely correlated, and again that only linear interactions between variables are being considered in the correlation matrix. For additional details on the relationships between variables, the reader should refer to Section S3 (ESI†). In summary, the analysis provided serves as a guide to determine the possible interactions between variables before building machine learning models. As we mentioned, this is due to the presence of multicollinearity which interferes with this bivariate analysis.^48^ To solve it, model interpretation will be based on the calculation of SHAP values which are designed to avoid it by assigning a larger relevance to one of the highly correlated variables.^46,48,49^ These analyses are very interesting for complex cases like the one studied here where additional constraints (*i.e.* obtain a homogeneous film after IJP deposition warranting a crack-free decomposition for an optimized pyrolyzed films thickness in the range of 1 micron) are imposed, since prompt intuition cannot be used.

### 3.2. Model for the variable NoD

The NoD deposited on a substrate by IJP has been used to develop the first Machine Learning (ML) model to validate the strategy followed in this study. This should go beyond the linear correlations interpretations allowed by the correlation matrix. We explained before that d*x* and d*y* are used to calculated it (eqn (1)) and, therefore, the predictions from the model should mainly depend on them. Nevertheless, we considered all features as descriptors except for the TVD that will not be used in this model given that it is calculated from the product between ADV and NoD. Essentially, the variables used for this model are AV, APL, ADV, d*x*, d*y* and amine.

**Fig. 3:**
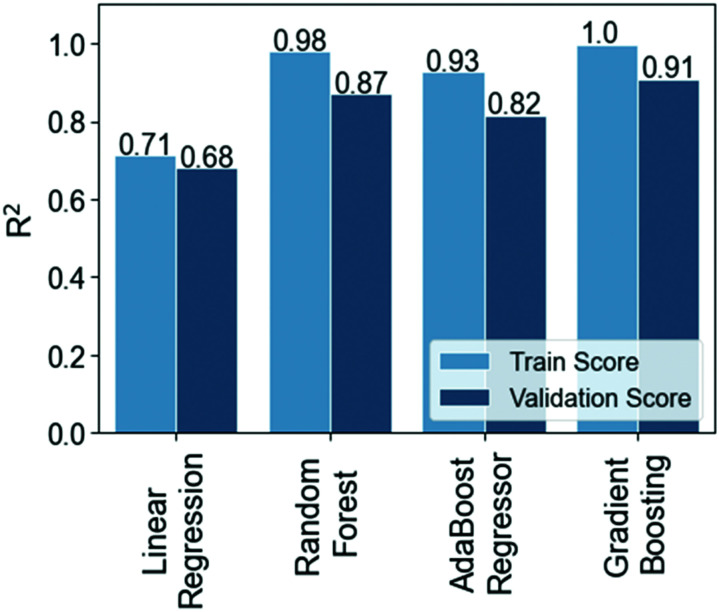
Precision (*R*^2^) values on the training and validation datasets for the no. of drops (NoD) models built with linear regression and ensemble methods using the default hyperparameters.

Four different ML algorithms were employed to predict the NoD based on these variables. The results procured by decision tree-based algorithms, *i.e.*, RF, AB and GB regressors, were compared with a linear regression model using the default hyperparameters (Fig. 3), revealing that the precisions (*R*^2^) for the models developed with ensemble methods are much larger in comparison to linear regression. The train and validation precisions are respectively 0.98 and 0.87 (RF), 0.93 and 0.82 (AB), and 1.0 and 0.91 (GB), while the linear regression has an *R*_train_^2^ = 0.71 and *R*_validation_^2^ = 0.68. This seems to imply that the relation between variables is not linear given that ensemble methods capture much better this type of relations. Although all models perform quite well, the characteristics and limitations of the dataset described in the ESI† (Sections S2 and S3), as well as the need to prevent overfitting make the RF regressor a much more reliable option due to the way the algorithm works (see Section S1, ESI†).^50^

After optimizing the parameters used in the RF algorithm to obtain the best model possible, *i.e.*, a no. of estimators of 30, minimum sample split of 3, and maximum depth of 10 (Section S4, ESI†), we evaluated it with the train and test sets which returned respectively scores of 0.98 and 0.94 (*R*^2^), and 175.84 and 288.83 (root mean squared error, RMSE). This suggests that the model can generalize quite well within the limitations of our experimental data, where the parameters used ensured the deposition of quite uniform films with none or little liquid movement. Our goal was to eventually obtain crack-free films after a subsequent pyrolysis step. Later, these films will be used for further treatment and investigation of the REBCO epitaxial growth and superconducting properties.

**Fig. 4:**
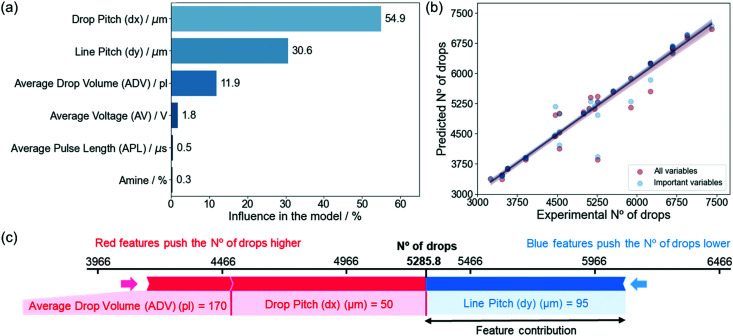
(a) Variable influence in the model created for the NoD. The percentage has been calculated by normalizing the average SHAP value obtained for each variable. (b) Predicted NoD as a function of the experimental values, comparing the model with all and only the important variables (d*x*, d*y* and ADV). (c) Prediction of the influence of model parameters on a specific no. of drops (NoD). The contribution of each feature is obtained based on their average values in the train set (〈d*x*〉 = 100.3 μm, 〈ADV〉 = 203.3 pl, and 〈d*y*〉 = 67.6 μm). The red color indicates the features that contribute increasing the predicted value, while blue is used for those that contribute to reduce it. The size of the arrow indicates how strong is the effect of each variable in the prediction.^46^

SHAP values are able to extract valuable information from black-box, non-parametric models which do not output an equation such as those derived from the RF algorithm.^46,51^ The global importance plots represent the contributions of independent variables on the modeled target variable and are calculated by aggregating the SHAP values for each individual prediction (Section S1, ESI†).^51^Eqn (1) shows that the NoD is calculated experimentally only from the d*x* and d*y*. However, the RF model and SHAP values clearly reveal that not only d*x* and d*y* contribute to define the NoD and the model predictions, but also the ADV (Fig. 4a). The influence of each variable on the model, calculated from the SHAP values, reveals that d*x* is the most important variable with a 54.9%, followed by d*y* with a 30.6% and the ADV in third place with an 11.9%. We also see that the AV, APL and amine have very little influence in defining the NoD with contributions between 0.3 and 1.8%. It is worth noting, that the ADV already considers the contribution of these three variables because it is experimentally defined by them during the IJP deposition process. Therefore, since they are negligible for the model, it is possible to remove them in order to simplify it without affecting too much its predictive power or even improving it (Section S4, ESI†).^52^

If we obtain some predictions for the NoD using both models (one with all variables and another with the most important) and compare them with the experimentally determined NoD in the test set (Fig. 4b), we see that the predictions obtained match very well with the experimental values. In addition, the difference between both models is quite insignificant as expected since both have very similar metrics (Section S4, ESI†).

In order to demonstrate the interest in this ML approximation, we have compared the predicted values obtained for the ML model with the most important experimental variables (ADV, d*x* and d*y*). This allows us to define which combination of experimental parameters would be required to obtain a specific NoD (Table S2, ESI†). This analysis also shows that a typical range for the NoD from 3500 to 6500 drops should guarantee a good and uniform coverage of the solution on a 5 × 5 mm^2^ substrate based on the experimental data used in our model. However, it must be noted that the ADV will also play an important role in determining the TVD, as we will see later, and this is known to affect the deposited film homogeneity and the final thickness after the pyrolysis process. Hence, this must also be considered when selecting the experimental conditions.

Based on these combinations, we built a plot that shows the contribution every independent variable has on the final output for a specific predicted NoD (Fig. 4c). In particular, if we wanted to deposit around 5286 ± 289 drops on a substrate which should lead to a TVD close to 1 μl, a d*x* of 50 μm, d*y* of 95 μm, and ADV of 170 pl would be required. It can also be seen that the d*x* and ADV have both a positive contribution (increase) on the NoD, while the d*y* contributes to decrease it. The relationships between a prediction and the experimental parameters determined by SHAP values in Fig. 4c are specific for each combination since they depend on the average values for each variable in the train set. Additional combinations of experimental variables and predicted NoD can be found in Section S4 (ESI†).

**Fig. 5:**
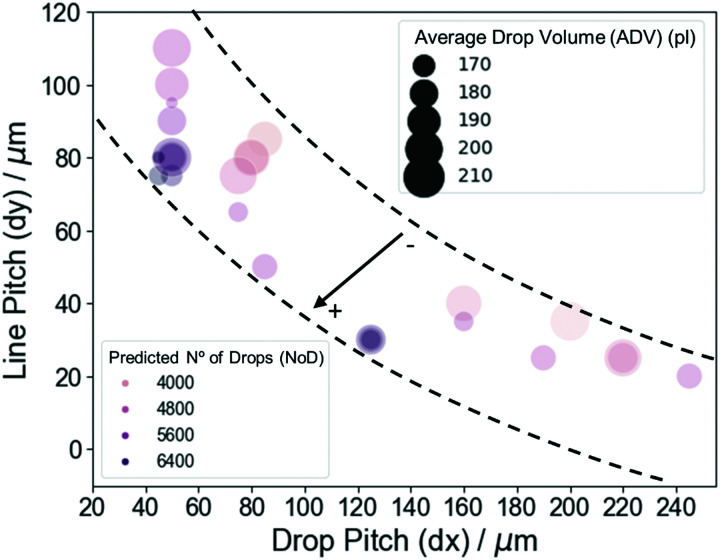
Scatterplot of the line pitch (d*y*) as a function of the drop pitch (d*x*) where the circle size corresponds to the value of the average drop volume (ADV) and color-coded we find the predicted value for the no. of drops (NoD). The dashed lines are a guide for the eye, while the arrow indicates the direction of increase for the predicted NoD.

As a final analysis of experimental interest from the ML model output, we represent the predicted values for the NoD color-coded in a phase-like diagram of d*x* and d*y* (Fig. 5), where the size of the circles corresponds to the ADV. The relation between d*x* and d*y* is quite linear with a *ρ* = −0.88, very similar to the correlation coefficient in Fig. 2. This plot demonstrates the relationship between the most important variables in the model and shows that despite the complex relationship between them and the NoD, one can extract the information on the values to be used for specific experimental conditions. We can identify two main regions where the predicted NoD is large, *i.e.*, 5600 ± 289 drops and above. The first one is located at values of d*x* between 75 and 90 μm, d*y* ∼ 50 μm, and ADV from 170 to 200 pl, approximately. The second region can be found at d*x* of ∼35 μm, d*y* around 120–130 μm, and ADV from 170 to 180 pl. Other areas with large NoD should roughly follow the bottom dashed line. On the other hand, the NoD will be 4800 ± 289 drops and smaller in areas close to the upper dashed line. For instance, d*x* between 100 and 120 μm, d*y* ∼ 50 μm, and ADV from 190 to 200 pl; or d*x* ∼ 40 μm, d*y* ∼ 200 μm, and ADV of 210 pl. We can also see that, in general, small ADV lead to large NoD values and *vice versa*, although the combination of d*x* and d*y* will have a more important contribution in defining the NoD. It is also worth noting that these results agree with the negative sign of the correlation coefficients for d*x* and ADV (*ρ* = −0.39 and −0.62), while disagree with the coefficient for d*y* (*ρ* = 0.05) (Fig. 2). However, we must remember that d*x* and d*y* are strongly correlated (*ρ* = −0.89). This again illustrates the importance of SHAP values in model interpretation for non-linear relationships, as well as independent variables that present multicollinearity.

In summary, the model developed allowed us to evaluate the importance of the experimental parameters in defining the NoD deposited on the substrates, as well as to comprehend the relation between them. Very precise predictions were obtained from the acquired experimental (test) data and using the RF-based models with *R*^2^ ∼ 0.94–0.95 and RMSE ∼ 267.31–288.83. We could also discern the influence of each variable on specific final values of the predicted NoD. We identified that the most important experimental parameters determining the resulting NoD are d*x*, d*y* and ADV. The general trend for these parameters indicates that small (large) d*x* and large (small) d*y* will keep a rather good homogeneity when using ADVs around 190–200 pl. However, increasing (decreasing) both d*x* and d*y* will result in more (less) drops deposited on the substrates which will then lead to the aforementioned inhomogeneous IJP depositions.

### 3.3. Model for the variable TVD

The second model we have built is for the TVD. This is a very relevant parameter to ensure that the films are homogeneous after the pyrolysis and do not present cracks, which again will guarantee that studies about the epitaxy of REBCO films and their physical properties can be performed properly. Since a critical thickness exists beyond which the strain generated during the pyrolysis will induce film cracking in CSD films.^15,18,53^ This is an essential problem that face many applications that require CSD films of large thickness (beyond few hundreds of nm). Hence, we developed a ML model to study which variables are the most important in defining the TVD. This will help in understanding the relation between experimental parameters that contribute to keep the homogeneous integrity of the deposited film after the pyrolysis. In addition, it will enable us to reach a precise control of these parameters and enhance the reproducibility of our films.

Although the TVD is defined by the product between NoD and ADV variables, in the initial stage, we decided to also include the AV, APL, d*x*, d*y* and amine. For the sake of completeness, we compared the precisions of linear regression and ensemble methods which all gave very similar values of *R*^2^ in the range of 0.89–0.99 for the validation set, including the linear regression model (Section S5, ESI†). This seems to imply that the relation between variables has a strong linear component which could come from the NoD (*ρ* = 0.91). However, based on the discussion made in the previous section, we selected the RF algorithm to build an enhanced version of the model for the TVD to identify any hidden non-linear relationships by considering the steps described in Section S1 (ESI†). The optimized parameters for the RF algorithm are a no. of estimators of 120, a minimum sample split of 3, and maximum depth of 10 (Section S5, ESI†). This model was evaluated on the train and test set, returning respective scores of 0.99 and 0.98 (*R*^2^), and 0.021 and 0.026 (RMSE) (Section S5, ESI†). These metrics show that the model is very adequate to predict the TVD within our experimental data, where only values associated to uniform depositions were considered.

**Fig. 6:**
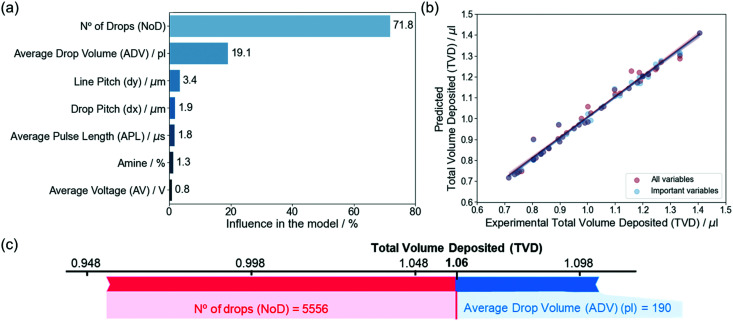
(a) Variable influence in the model created for the TVD. The percentage has been calculated by normalizing the average SHAP value obtained for each variable. (b) Predicted total volume deposited (TVD) as a function of the experimental values, comparing the model with all and only the important variables (no. of drops (NoD) and average drop volume (ADV)). (c) Prediction of the influence of model parameters on a specific total volume deposited (TVD). The contribution of each feature on the predicted TVD is obtained based on their average values in the train set (〈ADV〉 = 203.3 pl, and 〈NoD〉 = 4976.6 drops). The red color indicates the features that contribute increasing the predicted value, while blue is used for those that contribute to reduce it. The size of the arrow indicates how strong is the effect of each variable.^46^

Feature importance for the model that predicts the TVD (Fig. 6a) shows that the NoD has the largest weight with an influence of 71.8%, while the ADV is second with 19.1%, around 3.5 times lower. The remaining variables, *i.e.*, the d*x*, d*y*, APL, amine and AV, have very little significance in the model with percentages between 0.8 and 3.4%. These results agree with the parameters used to calculate the TVD (eqn (2)), but also highlight the weight of each feature in the TVD. Since the NoD is calculated from the d*x* and d*y*, which had influences of 54.9 and 30.6% (Section 3.2), it can be inferred that these two variables are also important for the TVD. As we mentioned before, the ADV already considers the contribution of the APL, amine and AV. These variables (d*x*, d*y*, APL, amine and AV) can be removed to build a simpler model without losing interpretability (Section S5, ESI†).

Fig. 6b shows the predictions of the TVD obtained with the models developed considering all the variables or only the NoD and the ADV on the test set. Equivalent results are reached for both models and the metrics are also very similar, 0.018–0.026 for the RMSE and 0.98–0.99 for the precision (Section S5, ESI†). Thus, it could be expected that the predicted values of the TVD are very similar between them, also precisely matching the experimental values from the test dataset. This simplification of variables enabled by the ML approach used, is very interesting from the experimental perspective, since it allows us to identify the most relevant parameters to consider.

The relationship between the predicted values for the TVD and the independent variables, *i.e.*, the experimental parameters that would be required to obtain them, has been extracted from the model with the most important variables (ADV and NoD) on the test set (Section S5, ESI†). As mentioned before, we were experimentally aiming for a TVD close to 1 μl on a 5 × 5 mm^2^ substrate which after a subsequent pyrolysis process would lead to films with a final thickness of about 1000 nm. Different combinations of experimental parameters can be used to get such result, which depend on the specific values of each independent variable, but the different combinations should all, in principle, ensure a homogeneous liquid distribution and eventually a pyrolyzed film of good quality. It must be remembered that our experimental data only considers combinations of parameters that produce rather uniform depositions. In addition, we can see that the TVD is largely affected by the combination we use.

Similarly to the NoD model, we built a plot that shows the relationship between the independent variables (NoD and ADV) with the TVD, we can see that to deposit a TVD of 1.06 ± 0.02 μl on a substrate we would require a NoD of 5556 drops (d*x* = 50 μm and d*y* = 90 μm) and a ADV of 190 pl. In this case, the NoD has a positive influence increasing the value of the predicted TVD, while ADV contributes to decrease it. As mentioned before, the predictions and their relation with the experimental parameters shown in Fig. 6c depend on the combination between experimental variables. Additional combinations of experimental variables and predicted TVD can be found in Section S5 (ESI†).

**Fig. 7:**
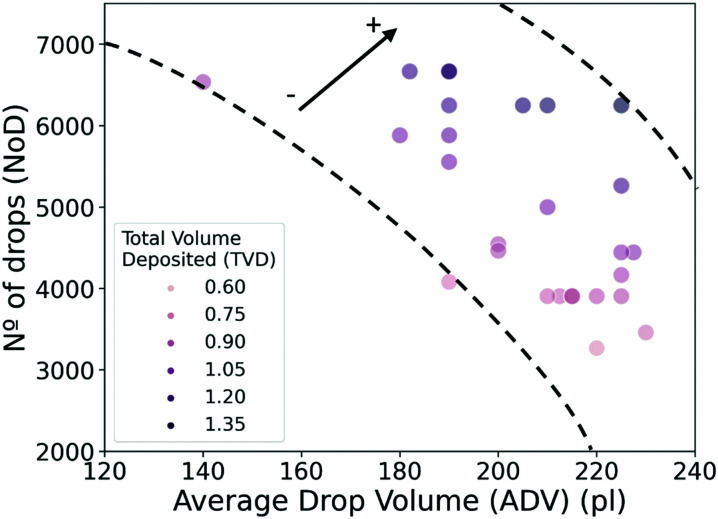
Scatterplot of the no. of drops (NoD) as a function of the average drop volume (ADV) and color-coded we find the predicted value for the total volume deposited (TVD). The dashed lines are a guide for the eye, while the arrow indicates the direction of increase for the predicted TVD.

Similarly to the NoD, a color-coded diagram of the TVD is represented as a function of the ADV and NoD (Fig. 7). This diagram summarizes all the previous results and shows the relationship of these variables with the TVD. We can see that large TVD values are mainly defined by the increase in NoD, while the contribution of the ADV seems less significant. In particular, TVD values of 1.05 ± 0.02 μl and above are located in a region of NoD between 5000 and 7000 drops, and ADV of 180–230 pl, while values of TVD smaller than 0.90 ± 0.02 μl are mostly found in the region of NoD below 5000 drops and ADV between 200 and 230 pl. From the experimental data used in the predictions, we can also see that large TVD are close to the upper dashed line, while small ones can be found near the dashed bottom line. If we consider the correlation coefficients (Fig. 2), we see that there is a high positive correlation of 0.91 between the TVD and NoD which agrees with the large weight of this variable in the model. On the other hand, the ADV has a negative correlation of −0.25. Although the negative value of the correlation seems to imply that the TVD should decrease with large values of the ADV, Fig. 7 clearly shows that the main reason for this is the use of lower NoD. The magnitude of the correlation coefficient for the ADV matches the weight of this feature in the model obtained from SHAP values (Fig. 6a). Finally, we can also define a range of experimental values for the ADV, between 190 and 210 pl, and NoD, between 5000 and 6000, that would lead to the previously mentioned TVD close to 1 μl in homogeneous layers.

It is also worth noting that these results agree with the negative sign of the correlation coefficients for d*x* and ADV (*ρ* = −0.39 and −0.62), while disagree with the coefficient for d*y* (*ρ* = 0.05) (Fig. 2). However, we must remember that d*x* and d*y* are strongly correlated (*ρ* = −0.89). This again illustrates the importance of SHAP values in model interpretation for non-linear relationships, as well as independent variables that present multicollinearity.

In summary, the predictions made on the experimental test data with the RF model show *R*^2^ of 0.98–0.99 and RMSE of 0.021–0.026, indicating that the TVD can be predicted with high precision. In addition, the most important parameters defining the TVD are the NoD (d*x* and d*y*) and the ADV. In particular, the NoD has a greater influence in achieving larger values of the TVD than the ADV.

## 4. Conclusions

We have developed a machine learning strategy that is used to analyze the results from inkjet printing deposition process of REBCO precursor solutions to extract meaningful information of the relationships between experimental parameters that enable homogeneous films. From the machine learning algorithms employed, the RF regressor was chosen to prevent overfitting due to the used dataset size (231 samples), and the noise detected in most features. Good performances (*R*^2^ > 0.87) and small errors (RMSE ∼ 267.31–288.83 (NoD) and 0.021–0.026 (TVD)) were achieved in the prediction of the NoD and TVD variables, indicating the suitability of the RF algorithm for the cases studied.

The SHAP values allowed us to extract information about the most important variables for each model, which is vital to understand which parameters must be tuned with higher priority. Particularly, we could identify that the NoD is mainly defined by d*x*, d*y* and ADV, whereas the TVD results from the contribution of the NoD and the ADV. Therefore, we can conclude that d*x*, d*y* and ADV define both the NoD and TVD.

The results obtained show the specific combinations we can do with the different experimental parameters to achieve specific values of NoD and TVD that match with our requirements for the quality and homogeneity of deposited films and their final thicknesses. Additionally, they provide us information of the relations between all parameters, enabling their proper tuning and optimization.

Finally, we demonstrate that machine learning can be used in the optimization of inkjet printing deposition, in particular we used it for the case of TLAG-REBCO precursor solutions, although the strategy and algorithms proposed here can be extended to other CSD functional solutions but also to the next steps of the REBCO film fabrication process by selecting the appropriate features and machine learning algorithms.

## Author contributions

T. P., X. O., S. R. and A. Q. conceived and designed the experiments, A. P. and A. Q. performed the IJP experiments, S. R. designed the ink precursors, N. J. and A. Q. developed the machine learning pipelines and models, N. J., A. P. and A. Q. characterized the films. The manuscript was written through contributions of all authors. All authors have given approval to the final version of the manuscript.

## Conflicts of interest

There are no conflicts to declare.

## Supplementary Material

TC-010-D1TC05913K-s001

TC-010-D1TC05913K-s002

TC-010-D1TC05913K-s003

TC-010-D1TC05913K-s004
