# Supplementary material for: Defining inkjet printing conditions of superconducting cuprate films through machine learning
Source: J Mater Chem C Mater. 2022 Apr 7;10(17):6885–95. doi: 10.1039/d1tc05913k (PMC9069570; doi:10.1039/d1tc05913k)

Notebook description

This notebook uses various Python-based machine learning libraries to build machine learning models capable of predicting the variable Nº of drops from other inkjet printing deposition experimental parameters.

Loading the libraries and Preprocessing

Import libraries

Load the libraries necessary to do data preparation and for machine learning.

In [ ]:

```
# EDA and plotting libraries
import numpy as np
import pandas as pd
import matplotlib.pyplot as plt
import matplotlib.gridspec as gridspec
import seaborn as sns
import itertools
import os

# The Patch library is imported to fix a legend issue, not allowing to show more th
from matplotlib.patches import Patch

# Library to format the axes
import matplotlib.ticker as mtick

# Plots appear in the same notebook cell
%matplotlib inline

# Seaborn style
sns.set_style("ticks", {"xtick.major.size": 8, "ytick.major.size": 8})
sns.set_context("paper")

# Activate latex text rendering for figure labels
from matplotlib import rc
rc('text', usetex=False)

# Models from Scikit-Learn
from sklearn.linear_model import LinearRegression
from sklearn.pipeline import Pipeline
from sklearn.ensemble import RandomForestRegressor, AdaBoostRegressor, GradientBoo

# Preprocessing and feature selection
from sklearn.preprocessing import StandardScaler, PolynomialFeatures
from sklearn.utils import shuffle

# Model Evaluations
from sklearn.model_selection import train_test_split, cross_val_score, KFold, cros
from sklearn.metrics import mean_squared_error, r2_score

# Tree interpreter
from treeinterpreter import treeinterpreter as ti

# Shap library to explain models intuitively
```

```
import shap
shap.initjs() # Activate Javascript option for Jupyter notebooks

# Function to display dataframes side_by_side
from IPython.display import display
from IPython.display import display_html
def display_side_by_side(*args):
    html_str=''
    for df in args:
        html_str+=df.to_html()
    display_html(html_str.replace('table','table style="display:inline"'),raw=True

# Ignore warning
import warnings
warnings.filterwarnings("ignore")
```

js

Load data

We will load the *data_final.csv* file with the preprocessed data.

In [ ]:
```
# Define the path
DATA_FINAL_PATH = "Data"
```

In [ ]:
```
# Load dataset
def load_data(path = DATA_FINAL_PATH):
    path = os.path.join(path, "data_final.csv")
    data_final = pd.read_csv(path)
    return data_final

data_final = load_data()

data_final.shape
```

Out[ ]:
```
(231, 8)
```

Check the dataset

We will check the head and tail of the dataset to confirm it has been loaded correctly.

In [ ]:
```
data_final.head()
```

Out[ ]:

	Average Voltage (V)	Average Pulse Length (us)	Average Drop Volume (pl)	Drop Pitch (um)	Line Pitch (um)	nº of drops	Total Volume Deposited (ul)	Amine (%)
**0**	135.0	26.0	185.0	175	75.0	1904.761905	0.352381	1.14
**1**	110.0	23.0	190.0	200	50.0	2500.000000	0.475000	1.14
**2**	135.0	26.0	190.0	175	50.0	2857.142857	0.542857	1.14
**3**	145.0	27.0	220.0	100	100.0	2500.000000	0.550000	1.14
**4**	130.0	26.0	219.0	95	95.0	2770.083102	0.606648	1.14

In [ ]:
```
data_final.tail()
```

Out[ ]:

	Average Voltage (V)	Average Pulse Length (us)	Average Drop Volume (pl)	Drop Pitch (um)	Line Pitch (um)	nº of drops	Total Volume Deposited (ul)	Amine (%)
**226**	155.000000	26.000000	225.0	50	80.0	6250.0	1.40625	2.00
**227**	155.000000	26.000000	225.0	50	80.0	6250.0	1.40625	2.00
**228**	155.000000	26.000000	225.0	50	80.0	6250.0	1.40625	2.00
**229**	153.500000	25.500000	238.0	50	80.0	6250.0	1.48750	2.00
**230**	149.873239	25.507042	180.0	50	50.0	10000.0	1.80000	1.14

Prediction of the variable Nº of drops

We will build a machine learning model to predict the **Nº of drops** deposited by considering all the dataset variables with the exception of the **Total Volume Deposited** that will be dropped from the dataset.

Select model variables and Train/Test split

We will select the variables used in the model, shuffle the data, and split the dataset into X and y. We split the data into train and test datasets by using the ratio 80:20.

In [ ]:

```
def split_data(data, test_ratio):

    # Drop columns
    data_NOD = data_final.drop(labels=['Total Volume Deposited (ul)'], axis=1, inpl

    # Check the dataset
    data_NOD

    # Shuffle the data
    data_NOD = shuffle(data_NOD, random_state = 42) # A random state was set to kee

    data_NOD.reset_index(inplace = True, drop = True)

    # Split the data set into X and y
    X_NOD = data_NOD.drop(labels=['nº of drops'], axis=1)
    y_NOD = data_NOD['nº of drops']

    # Split the training data into training and test sets (80:20)
    X_train_NOD, X_test_NOD, y_train_NOD, y_test_NOD = train_test_split(X_NOD,
                                                       y_NOD,
                                                       test_size=test_ratio, random_sta

    return X_train_NOD, y_train_NOD, X_test_NOD, y_test_NOD


X_train_NOD, y_train_NOD, X_test_NOD, y_test_NOD = split_data(data_final, 0.2)
```

In [ ]:

```
# Check the range of values from all columns
X_train_NOD.describe()
```

Out[ ]:

	Average Voltage (V)	Average Pulse Length (us)	Average Drop Volume (pl)	Drop Pitch (um)	Line Pitch (um)	Amine (%)
**count**	184.000000	184.000000	184.000000	184.000000	184.000000	184.000000
**mean**	150.313036	25.571226	203.336957	100.326087	67.562500	1.652391
**std**	22.363363	1.031120	19.421498	62.054112	25.930804	0.416547
**min**	95.000000	20.000000	140.000000	45.000000	20.000000	0.000000
**25%**	136.000000	25.000000	190.000000	50.000000	40.000000	1.140000
**50%**	149.936620	25.507042	205.000000	80.000000	80.000000	2.000000
**75%**	155.000000	26.000000	220.000000	160.000000	85.000000	2.000000
**max**	234.000000	30.000000	240.000000	250.000000	100.000000	2.300000

In [ ]:

```
# Check the range of values from all columns
X_test_NOD.describe()
```

Out[ ]:

	Average Voltage (V)	Average Pulse Length (us)	Average Drop Volume (pl)	Drop Pitch (um)	Line Pitch (um)	Amine (%)
**count**	47.000000	47.000000	47.000000	47.000000	47.000000	47.000000
**mean**	148.151483	25.255769	202.957447	107.659574	63.191489	1.668085
**std**	20.718978	1.272166	19.617492	64.898341	27.295197	0.400166
**min**	95.000000	20.000000	165.000000	45.000000	20.000000	1.140000
**25%**	137.500000	25.000000	188.500000	50.000000	35.000000	1.140000
**50%**	150.000000	25.500000	202.500000	85.000000	75.000000	2.000000
**75%**	157.500000	26.000000	220.000000	160.000000	85.000000	2.000000
**max**	233.000000	27.000000	235.000000	245.000000	110.000000	2.300000

In [ ]:

```
# Check the range of values from all columns
y_train_NOD.describe()
```

Out[ ]:

```
count      184.000000
mean      4976.595225
std       1224.097682
min       1904.761905
25%       3906.250000
50%       5000.000000
75%       5882.352941
max      10000.000000
Name: nº of drops, dtype: float64
```

In [ ]:

```
# Check the range of values from all columns
y_test_NOD.describe()
```

Out[ ]:

```
count      47.000000
mean     5044.874953
std      1219.078954
min      3246.753247
25%      3906.250000
50%      5000.000000
75%      6250.000000
max      7407.407407
Name: nº of drops, dtype: float64
```

Build models without hyperparameters

We will build the models with all default hyperparameters.

In [ ]:
```
# Dictionary with models
models = {
          'Linear Regression': LinearRegression(),
          'Random Forest': RandomForestRegressor(),
          'AdaBoost Regressor': AdaBoostRegressor(),
          'Gradient Boosting': GradientBoostingRegressor()
         }

# Create a function to fit and score models
def train_cross_val(models, X_train, y_train, score_name):

    # Set random seed
    np.random.seed(42)

    # Make a dictionary to keep model scores
    model_validation_scores = {}

    # K-Fold cross validation
    cv = KFold(n_splits = 5) # n_splits = 5 means 80:20 training:validation

    # Loop through models
    for name, model in models.items():
        # Fit the model to the training data
        model.fit(X_train, y_train)

        # Cross-validation
        model_validation_scores[name] = cross_validate(model, X_train, y_train,
                                                       cv = cv, scoring = score_nai

    return model_validation_scores
```

In [ ]:
```
# Check scores
cross_val_scores = train_cross_val(models=models, X_train=X_train_NOD, y_train=y_ti
```

In [ ]:
```
# Get values from each model
def get_dict_scores():

    # Define empty dictionaries
    train_score = {}
    test_score = {}

    # Loop through score dictionary values
    for k1, v1 in cross_val_scores.items():
        for k2, v2 in v1.items():
            if k2 == 'train_score':
                v2 = np.mean(v2)
                train_score[k1] = abs(v2)
            elif k2 == 'test_score':
                v2 = np.mean(v2)
                test_score[k1] = abs(v2)

    return train_score, test_score
```

In [ ]:
```
# Define function to merge scores and create a dataframe
def merge_scores():

    dict_scores = get_dict_scores()
    train_score = dict_scores[0]
```

```
    test_score = dict_scores[1]

    # Create a data frame with the scores from the train_score dictionary
    train_df = pd.DataFrame.from_dict(train_score, orient='index')
    train_df.reset_index(inplace = True)
    train_df = train_df.rename(columns = {'index': 'Models', 0: 'Avg. Score'})

    # Create a data frame with the scores from the test_score dictionary
    test_df = pd.DataFrame.from_dict(test_score, orient='index')
    test_df.reset_index(inplace = True)
    test_df = test_df.rename(columns = {'index': 'Models', 0: 'Avg. Score'})

    # Merge both dataframes
    df = pd.merge(train_df, test_df[['Models','Avg. Score']],on='Models', how='lef
    df = df.rename(columns = {'Avg. Score_x': 'Train Avg. Score', 'Avg. Score_y':

    # Melt dictionary by Models column
    model_compare = df.melt(id_vars = 'Models')
    model_compare = model_compare.rename(columns = {'variable': 'Score', 'value':

    return model_compare

merge_scores()
```

Out[ ]:

	Models	Score	Value
**0**	Linear Regression	Train Avg. Score	0.712472
**1**	Random Forest	Train Avg. Score	0.980316
**2**	AdaBoost Regressor	Train Avg. Score	0.928186
**3**	Gradient Boosting	Train Avg. Score	0.997853
**4**	Linear Regression	Test Avg. Score	0.680699
**5**	Random Forest	Test Avg. Score	0.870527
**6**	AdaBoost Regressor	Test Avg. Score	0.816068
**7**	Gradient Boosting	Test Avg. Score	0.909477

Figure 3

In [ ]:

```
def plot_scores():

    # Call the merge_scores function
    model_compare = merge_scores()

    # Plot the scores
    fig, ax = plt.subplots(figsize=(7,5))

    sns.barplot(x = 'Models', y = 'Value', data = model_compare, hue = 'Score', pa

    plt.xticks(fontsize=20, rotation=90);
    plt.xlabel("", fontsize=20);
    plt.yticks(fontsize=18);
    plt.ylabel(r"R$^2$", fontsize=20);

    plt.ylim([0, 1.1])
    plt.yticks(np.arange(0, 1.2, 0.2))

    model_names = ['Linear \nRegression', 'Random \nForest', 'AdaBoost \nRegressor
    ax.set_xticklabels(model_names)
```

```
    plt.legend(prop={"size":16}, loc = 4)

    x_offset = 0.00
    y_offset = 0.00

    for p in ax.patches:
        ax.annotate(str(round(p.get_height(),2)), (p.get_x() + 0.02, p.get_height(

    return plt.show()

plot_scores()
```

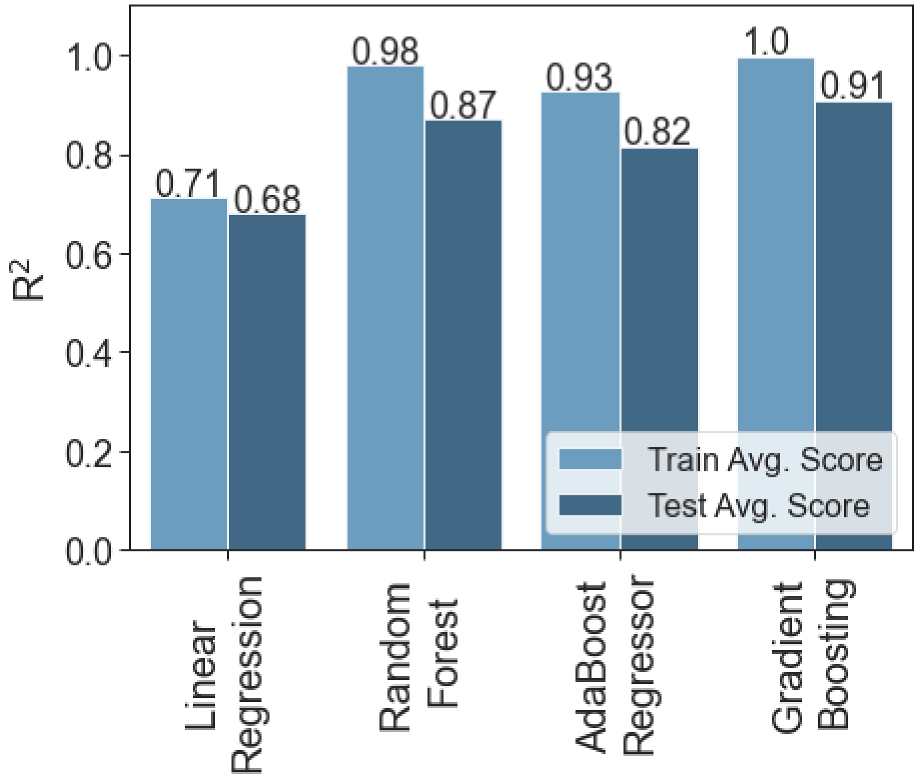

Evaluation function to calculate different error metrics

In [ ]:

```
# Create function to evaluate our model
def show_scores(model, X_train, X_test, y_train, y_test):
    train_preds = model.predict(X_train)
    val_preds = model.predict(X_test)
    scores = {"Train MSE": mean_squared_error(y_train, train_preds, squared=True),
              "Test MSE": mean_squared_error(y_test, val_preds, squared=True),
              "Train RMSE": mean_squared_error(y_train, train_preds, squared=False
              "Test RMSE": mean_squared_error(y_test, val_preds, squared=False),
              "Train R^2": model.score(X_train, y_train),
              "Test R^2": model.score(X_test, y_test)}
    return scores
```

Optimizing the Random Forest Regressor

We will optimize the hyperparameters for the Random Forest Regressor model, as well as explore the evolution of different metrics.

Number of estimators

Here, we will plot the evolution of the RMSE and R^2 as a function of the *n_estimators*, *min_sample_split* and *max_depth parameters*.

In [ ]:
```
# Function to evaluate the hyperparameters for the number of trees
def n_trees_tuning(X_train, y_train, n_trees, tree_step, cross_val):

    ### Iterate over the number of estimators in the Random Forest

    # Create a list to store the metrics
    index_estimators = []
    r2_estimators = []
    rmse_estimators = []

    cv = KFold(n_splits = cross_val) # n_splits = 5 means 80:20 training:validatio

    # Iterate over a hyperparameter
    for i in np.arange(0, n_trees, tree_step):
        test_model_rf_NOD = RandomForestRegressor(n_estimators = i,
                                                min_samples_leaf = 1,
                                                min_samples_split = 3,
                                                max_features = 'auto',
                                                max_depth = 10,
                                                max_samples = None,
                                                random_state = 42)

        val_scores_r2 = cross_val_score(test_model_rf_NOD, X_train, y_train, cv = 
        val_scores_rmse = cross_val_score(test_model_rf_NOD, X_train, y_train, cv 
                                                scoring = 'neg_root_mean_squared_error')

        # Store the scores in a list
        index_estimators.append(i)
        r2_estimators.append(val_scores_r2.mean())
        rmse_estimators.append(val_scores_rmse.mean())

    return index_estimators, r2_estimators, rmse_estimators
```

In [ ]:
```
index_estimators, r2_estimators, rmse_estimators = n_trees_tuning(X_train_NOD, y_tr
```

In [ ]:
```
# Create a dataframe with the number of estimators
rf_estimators_NOD = pd.DataFrame(
    {
        'Nº of Estimators': index_estimators,
        'R2 validation': r2_estimators,
        'RMSE validation': rmse_estimators
    }
)

# Convert negative RMSE to positive
rf_estimators_NOD['RMSE validation'] = rf_estimators_NOD['RMSE validation'].abs()
```

In [ ]:
```
# Function to evaluate the hyperparameters for the number of trees
def split_tuning(X_train, y_train, min_split, split_step, cross_val):

    ### Iterate over the min_sample_split in the Random Forest

    # Create a list to store the metrics
    index_min_sample = []
    rmse_min_sample = []
    r2_min_sample = []

    cv = KFold(n_splits = cross_val) # n_splits = 5 means 80:20 training:validatio
```

```
    # Iterate over a hyperparameter
    for i in np.arange(2, min_split, split_step):
        test_model_rf_NOD = RandomForestRegressor(n_estimators = 30,
                                                  min_samples_leaf = 1,
                                                  min_samples_split = i,
                                                  max_features = 'auto',
                                                  max_depth = 10,
                                                  max_samples = None,
                                                  random_state = 42)

        val_scores_r2_min_sample = cross_val_score(test_model_rf_NOD, X_train, y_tr
                                                   cv = cv, scoring = 'r2')
        val_scores_rmse_min_sample = cross_val_score(test_model_rf_NOD, X_train, y_
                                                     cv = cv, scoring = 'neg_root_me

        # Store the scores in a list
        index_min_sample.append(i)
        r2_min_sample.append(val_scores_r2_min_sample.mean())
        rmse_min_sample.append(val_scores_rmse_min_sample.mean())

    return index_min_sample, r2_min_sample, rmse_min_sample
```

In [ ]:
```
index_min_sample, r2_min_sample, rmse_min_sample = split_tuning(X_train_NOD, y_tra
```

In [ ]:
```
# Create a dataframe with the min_sample_split
rf_split_NOD = pd.DataFrame(
    {
        'Minimum Sample Split': index_min_sample,
        'R2 validation': r2_min_sample,
        'RMSE validation': rmse_min_sample
    }
)

# Convert negative RMSE to positive
rf_split_NOD['RMSE validation'] = rf_split_NOD['RMSE validation'].abs()
```

In [ ]:
```
# Function to evaluate the hyperparameters for the number of trees
def depth_tuning(X_train, y_train, depth, depth_step, cross_val):
    ### Iterate over the max_depth in the Random Forest

    # Create a list to store the metrics
    index_depth = []
    rmse_depth = []
    r2_depth = []

    cv = KFold(n_splits = cross_val) # n_splits = 5 means 80:20 training:validation

    # Iterate over a hyperparameter
    for i in np.arange(2, depth, depth_step):
        test_model_rf_NOD = RandomForestRegressor(n_estimators = 30,
                                                  min_samples_leaf = 1,
                                                  min_samples_split = 3,
                                                  max_features = 'auto',
                                                  max_depth = i,
                                                  max_samples = None,
                                                  random_state = 42)
        val_scores_r2_depth = cross_val_score(test_model_rf_NOD, X_train, y_train,
                                              cv = cv, scoring = 'r2')
        val_scores_rmse_depth = cross_val_score(test_model_rf_NOD, X_train, y_trai
                                                cv = cv, scoring = 'neg_root_mean_

        # Store the scores in a list
        index_depth.append(i)
```

```
        r2_depth.append(val_scores_r2_depth.mean())
        rmse_depth.append(val_scores_rmse_depth.mean())

    return index_depth, r2_depth, rmse_depth
```

In [ ]:
```
index_depth, r2_depth, rmse_depth = depth_tuning(X_train_NOD, y_train_NOD, depth=2(
```

In [ ]:
```
# Create a dataframe with the max_depth
rf_depth_NOD = pd.DataFrame(
    {
        'Maximum Depth': index_depth,
        'R2 validation': r2_depth,
        'RMSE validation': rmse_depth
    }
)

# Convert negative RMSE to positive
rf_depth_NOD['RMSE validation'] = rf_depth_NOD['RMSE validation'].abs()
```

Figure S7

In [ ]:
```
# Define the grid
fig, axs = plt.subplots(nrows = 1, ncols = 3, figsize=(14,4))

# Plot the different metrics as a function of the number of estimators
g1_rmse_scatter = sns.scatterplot(x = 'Nº of Estimators', y = 'RMSE validation', s=
                                  color = 'steelblue', data = rf_estimators_NOD, a>
g1_rmse_line = sns.lineplot(x = 'Nº of Estimators', y = 'RMSE validation', linewid
                            color = 'steelblue', data = rf_estimators_NOD, ax = ax:

# Twin object for two different y-axis on the sample plot axs[0]
ax1_twin=axs[0].twinx()

g1_r2_scatter = sns.scatterplot(x = 'Nº of Estimators', y = 'R2 validation', s=70,
                                color = 'chocolate', data = rf_estimators_NOD)
g1_r2_line = sns.lineplot(x = 'Nº of Estimators', y = 'R2 validation', linewidth =
                          color = 'chocolate', data = rf_estimators_NOD)

# Plot the different metrics as a function of the minimum sample split
g2_rmse_scatter = sns.scatterplot(x = 'Minimum Sample Split', y = 'RMSE validation
                                  color = 'steelblue', data = rf_split_NOD, ax = a>
g2_rmse_line = sns.lineplot(x = 'Minimum Sample Split', y = 'RMSE validation', line
                            color = 'steelblue', data = rf_split_NOD, ax = axs[1])

# Twin object for two different y-axis on the sample plot axs[1]
ax2_twin=axs[1].twinx()

g2_r2_scatter = sns.scatterplot(x = 'Minimum Sample Split', y = 'R2 validation', s=
                                color = 'chocolate', data = rf_split_NOD, ax = ax2_
g2_r2_line = sns.lineplot(x = 'Minimum Sample Split', y = 'R2 validation', linewid
                          color = 'chocolate', data = rf_split_NOD, ax = ax2_twin)

# Plot the different metrics as a function of the maximum depth
g3_rmse_scatter = sns.scatterplot(x = 'Maximum Depth', y = 'RMSE validation', s=70
                                  color = 'steelblue', data = rf_depth_NOD, ax = a>
g3_rmse_line = sns.lineplot(x = 'Maximum Depth', y = 'RMSE validation', linewidth =
                            color = 'steelblue', data = rf_depth_NOD, ax = axs[2])

# Twin object for two different y-axis on the sample plot axs[2]
ax3_twin=axs[2].twinx()

g3_r2_scatter = sns.scatterplot(x = 'Maximum Depth', y = 'R2 validation', s=70,
```

```
                                color = 'chocolate', data = rf_depth_NOD, ax = ax3_
g3_r2_line = sns.lineplot(x = 'Maximum Depth', y = 'R2 validation', linewidth = 2,
                          color = 'chocolate', data = rf_depth_NOD, ax = ax3_twin)

### Customize the axs[0] plot

# Customize the RMSE plot
axs[0].set_xlabel('Nº of estimators', fontsize = 20)
axs[0].set_ylabel('RMSE', fontsize = 20, color = 'steelblue')
axs[0].tick_params(labelsize = 18)
axs[0].set_xlim([0, 500])
axs[0].set_ylim([390, 450])
axs[0].set_xticks(np.arange(0, 510, 50))
axs[0].set_yticks(np.arange(390, 460, 10))
axs[0].spines['left'].set_color('steelblue')
axs[0].tick_params(axis='y', colors='steelblue')

# Customize the R2 plot
ax1_twin.set_ylabel(r'R$^2$', fontsize = 20, color = 'chocolate')
ax1_twin.tick_params(axis = 'y', labelsize = 18)
ax1_twin.set_ylim([0.84, 0.88])
ax1_twin.set_yticks(np.arange(0.84, 0.89, 0.01))
ax1_twin.spines['right'].set_color('chocolate')
ax1_twin.tick_params(axis='y', colors='chocolate')

### Customize the axs[1] plot

# Customize the RMSE plot
axs[1].set_xlabel('Minimum Sample Split', fontsize = 20)
axs[1].set_ylabel('RMSE', fontsize = 20, color = 'steelblue')
axs[1].tick_params(labelsize = 18)
axs[1].set_xlim([1, 11])
axs[1].set_ylim([390, 470])
axs[1].set_xticks(np.arange(1, 12, 1))
axs[1].set_yticks(np.arange(390, 480, 10))
axs[1].spines['left'].set_color('steelblue')
axs[1].tick_params(axis='y', colors='steelblue')

# Customize the R2 plot
ax2_twin.set_ylabel(r'R$^2$', fontsize = 20, color = 'chocolate')
ax2_twin.tick_params(axis = 'y', labelsize = 18, color = 'chocolate')
ax2_twin.set_ylim([0.84, 0.89])
ax2_twin.set_yticks(np.arange(0.84, 0.89, 0.01))
ax2_twin.spines['right'].set_color('chocolate')
ax2_twin.tick_params(axis='y', colors='chocolate')

### Customize the axs[2] plot

# Customize the RMSE plot
axs[2].set_xlabel('Maximum Depth', fontsize = 20)
axs[2].set_ylabel('RMSE', fontsize = 20, color = 'steelblue')
axs[2].tick_params(labelsize = 18)
axs[2].set_xlim([1, 20])
axs[2].set_ylim([380, 730])
axs[2].set_xticks(np.arange(1, 21, 2))
axs[2].set_yticks(np.arange(380, 730, 40))
axs[2].spines['left'].set_color('steelblue')
axs[2].tick_params(axis='y', colors='steelblue')

# Customize the R2 plot
ax3_twin.set_ylabel(r'R$^2$', fontsize = 20, color = 'chocolate')
ax3_twin.tick_params(axis = 'y', labelsize = 18, color = 'chocolate')
ax3_twin.set_ylim([0.60, 0.90])
ax3_twin.set_yticks(np.arange(0.60, 0.90, 0.05))
```

```
ax3_twin.spines['right'].set_color('chocolate')
ax3_twin.tick_params(axis='y', colors='chocolate')

# Figure labels
fig.text(-0.003, 1.35, '(a)', va='center', rotation='horizontal', fontsize = 20)
fig.text(0.46, 1.35, '(b)', va='center', rotation='horizontal', fontsize = 20)
fig.text(0.925, 1.35, '(c)', va='center', rotation='horizontal', fontsize = 20)

# set the spacing between subplots
plt.subplots_adjust(left=0.0,
                    bottom=0.0,
                    right=0.9,
                    top=0.9,
                    wspace=2.5,
                    hspace=0.5)

plt.tight_layout(rect=(0, 0, 1.4, 1.4));
```

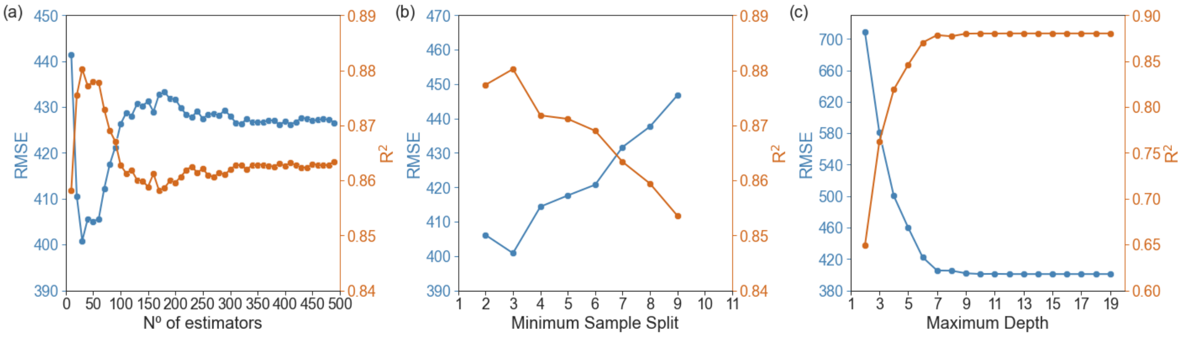

Train a model with the best parameters

The Random Forest will be trained on the best parameters to calculate the values of the evaluation metrics. Then, it will be fit to the whole train set and evaluated to a final test set.

In [ ]:

```
%%time
cv = KFold(n_splits = 5) # n_splits = 5 means 80:20 training:validation

# Most ideal hyperparameters
ideal_model_rf_NOD = RandomForestRegressor(n_estimators = 30,
                                           min_samples_leaf = 1,
                                           min_samples_split = 3,
                                           max_features = 'auto',
                                           max_depth = 10,
                                           max_samples = None,
                                           random_state = 42)

val_scores_r2 = cross_validate(ideal_model_rf_NOD, X_train_NOD, y_train_NOD,
                               cv = cv, scoring = 'r2', return_train_score = True)
val_scores_rmse = cross_validate(ideal_model_rf_NOD, X_train_NOD, y_train_NOD,
                                 cv = cv, scoring = 'neg_root_mean_squared_error',

# Scores
val_scores_r2
val_scores_rmse
```

```
CPU times: total: 516 ms
Wall time: 508 ms
```

Out[ ]:
```
{'fit_time': array([0.03696132, 0.04200268, 0.04402924, 0.04198599, 0.04303741]),
 'score_time': array([0.00600791, 0.00500917, 0.00398827, 0.004987  , 0.0050098
9]),
 'test_score': array([-276.77410026, -285.82456169, -584.75142429, -331.66774788,
       -525.06872448]),
 'train_score': array([-162.3777209 , -184.74833427, -157.9364842 , -213.56670322,
       -201.8276467 ])}
```

Calculate mean value of R2

In [ ]:
```
# Train score
val_scores_r2['train_score'].mean()
```

Out[ ]:
```
0.9769446690335784
```

In [ ]:
```
# Validation score
val_scores_r2['test_score'].mean()
```

Out[ ]:
```
0.8802218530548738
```

Calculate mean value of RMSE

In [ ]:
```
# Train score
abs(val_scores_rmse['train_score'].mean())
```

Out[ ]:
```
184.09137785954618
```

In [ ]:
```
# Validation score
abs(val_scores_rmse['test_score'].mean())
```

Out[ ]:
```
400.8173117198779
```

Fit the model on the whole training set and calculate the scores for the test set

In [ ]:
```
# Fit the ideal model on the whole training set
ideal_model_rf_NOD.fit(X_train_NOD, y_train_NOD)

# Calculate the scores for the test set
ideal_scores_NOD = show_scores(ideal_model_rf_NOD, X_train_NOD, X_test_NOD, y_trai
ideal_scores_NOD
```

Out[ ]:
```
{'Train MSE': 30918.26552343868,
 'Test MSE': 83421.34959151586,
 'Train RMSE': 175.83590510313496,
 'Test RMSE': 288.8275429932468,
 'Train R^2': 0.9792532676300386,
 'Test R^2': 0.9426473392659994}
```

The scores for the train set shown here correspond to the ones for the whole set without cross-validation.

Predictions with the optimized model and graphical representation

In [ ]:
```
# Make predictions on test data and save them
y_preds_rf_NOD = ideal_model_rf_NOD.predict(X_test_NOD)

# View predictions
y_preds_rf_NOD
```

Out[ ]:
```
array([4446.26999158, 5555.55555556, 5000.        , 6640.52287582,
       5000.        , 5152.10708215, 3462.49500932, 3906.25      ,
       6632.86847503, 7104.68106996, 5425.86973342, 6886.0899298 ,
       6250.        , 3462.49500932, 3893.66931727, 6935.47264585,
       4535.01683502, 6609.34744268, 5132.35044018, 3906.25      ,
       5404.97703355, 3906.25      , 3636.57518563, 6250.        ,
       6514.63990154, 6250.        , 4961.77800374, 5286.72170623,
       4444.44444444, 4129.08516381, 3462.49500932, 3850.91547331,
       3636.57518563, 5121.76606754, 5882.35294118, 6669.75308642,
       5541.50900126, 3382.65518329, 3462.49500932, 3854.31288256,
       3906.25      , 5000.        , 3367.73225938, 6623.29851219,
       5547.96371411, 3636.57518563, 5038.18369453])
```

In [ ]:
```
# Plot Y-train vs Y-preds for RFR
fig, ax = plt.subplots(figsize=(9, 7))

sns.regplot(x = y_test_NOD,
            y= y_preds_rf_NOD, scatter_kws={"color":"steelblue","alpha":0.3,"s":10(
            line_kws={"color":"black","alpha":0.7,"lw":3});

ax.tick_params(labelsize=20)
ax.set_xlabel("Experimental Nº of drops",fontsize=24)
ax.set_ylabel("Predicted Nº of drops",fontsize=24)

plt.xticks(np.arange(3000,8100, 500))
plt.yticks(np.arange(3000,8100, 500))
plt.xlim([3000, 7750])
plt.ylim([3000, 7750])
```

Out[ ]: (3000.0, 7750.0)

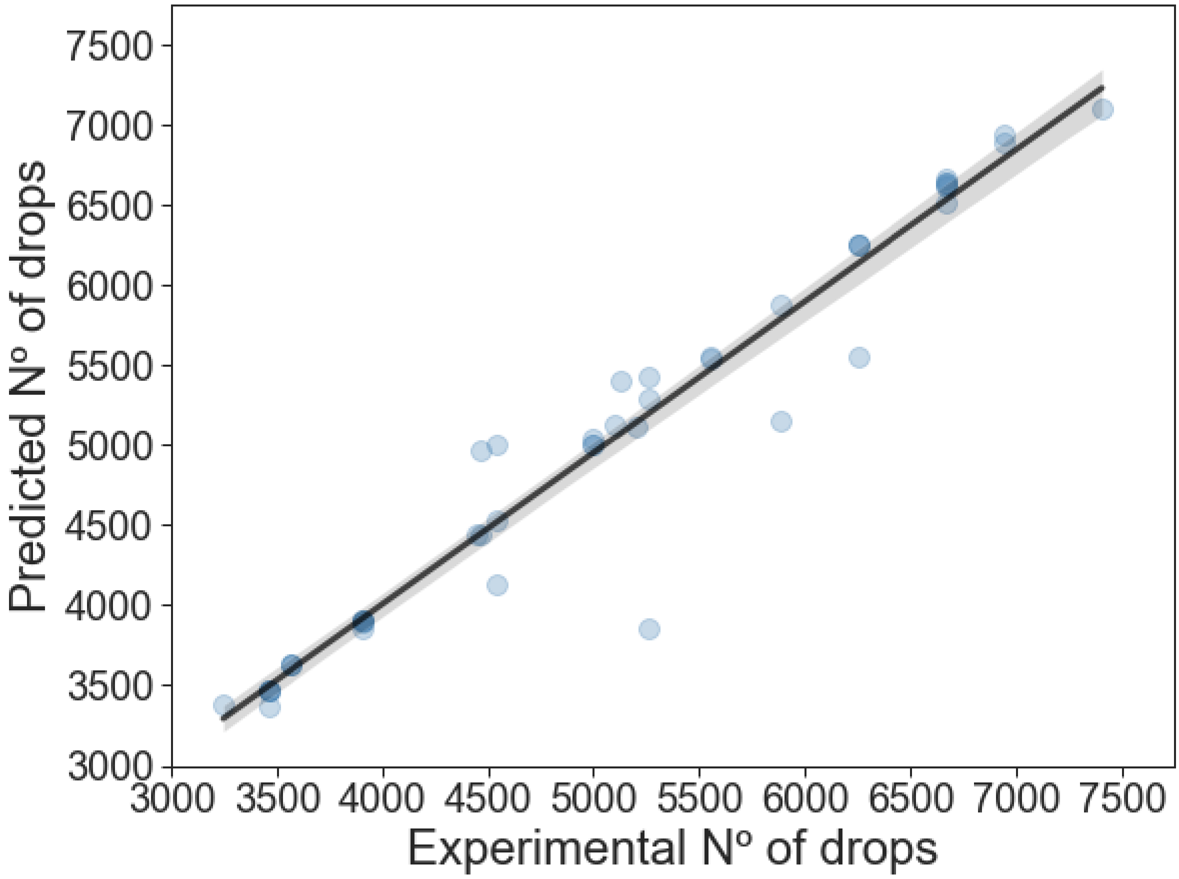

The above plot with the results from the predictions vs the experimentally determined Nº of drops shows that the model can predict with high accuracy the experimental values, as expected due to the high accuracy obtained.

Calculation of the variable importance

We will plot the variable importance which lists the variables of the model by order of importance in descending order. The top variables contribute more to the model than the bottom ones and thus have high predictive power. The SHAP library is being used to calculate it, and it is based on the mean absolute value of the SHAP values for each feature.

Check https://github.com/slundberg/shap for details on the implementation of the SHAP library.

Figure 4a

In [ ]:

```
# Explain the model's predictions using the SHAP library
explainer = shap.TreeExplainer(ideal_model_rf_NOD, X_train_NOD)
shap_values = explainer.shap_values(X_test_NOD)

# Create dataframe with SHAP values
shap_df = pd.DataFrame(shap_values, columns = X_test_NOD.columns)

# Calculate the average value of the absolute SHAP values for each feature
avg_values = shap_df.apply(lambda x: x.abs().mean(), axis = 0)

# Calculate the percentages
avg_values_percentage = (avg_values/avg_values.sum()) * 100
avg_values_percentage.sort_values(ascending = False, inplace = True)

# Plot the SHAP values
sns.barplot(x = avg_values_percentage, y = avg_values_percentage.index, palette = '

# Customize the plot
fig = plt.gcf()
fig.set_figheight(8)
fig.set_figwidth(10)
ax = plt.gca()
ax.set_xlabel('Influence in the model / %', fontsize=26)
ax.tick_params(labelsize=24)
ax.set_xlim([0, 65])

var_shap = [r'Drop Pitch (dx) / $\mu$m', r'Line Pitch (dy) / $\mu$m', 'Average Drop
            'Average Voltage (AV) / V', r'Average Pulse Length (APL) / $\mu$s', 'Ar

ax.set_yticklabels(var_shap)

# Add annotations
for p in ax.patches:
    ax.annotate(str(round(p.get_width(),1)), xy = (p.get_width()+1, p.get_y() + p.g

plt.show()
```

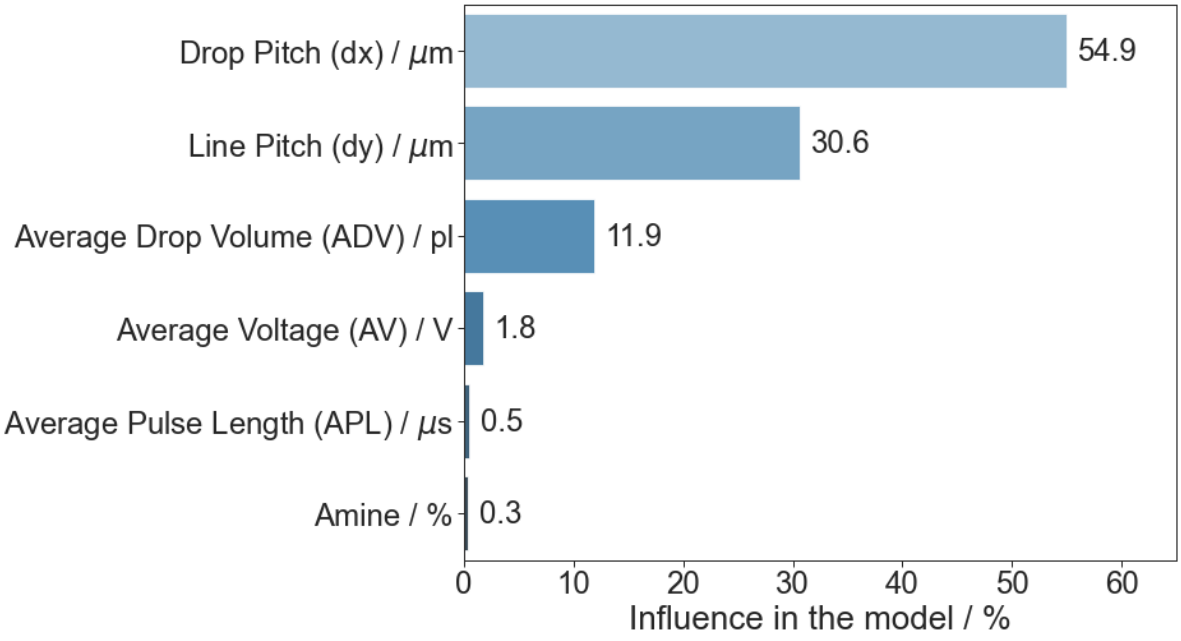

Prediction of the variable **Nº of drops** by dropping unimportant features

We will remove the previous unimportant variables from the model to determine how the accuracy and RMSE vary.

In [ ]:
```
# Drop columns from train and test sets
X_train_NOD_reduced = X_train_NOD.drop(labels=[
                                 'Average Voltage (V)', 'Average Pulse Length (us)
                                ], axis=1, inplace=False)

X_test_NOD_reduced = X_test_NOD.drop(labels=[
                                 'Average Voltage (V)', 'Average Pulse Length (us)
                                ], axis=1, inplace=False)
```

Train a new model with the reduced dataset

We will train a RF model with the reduced dataset

In [ ]:
```
%%time

# Most ideal hyperparameters
ideal_model_rf_NOD_reduced = RandomForestRegressor(n_estimators = 30,
                                    min_samples_leaf = 1,
                                    min_samples_split = 3,
                                    max_features = 'auto',
                                    max_depth = 10,
                                    max_samples = None,
                                    random_state = 42)
ideal_model_rf_NOD_reduced.fit(X_train_NOD_reduced, y_train_NOD)
```

```
CPU times: total: 31.2 ms
Wall time: 35 ms
```

Out[ ]:
```
RandomForestRegressor(max_depth=10, min_samples_split=3, n_estimators=30,
                      random_state=42)
```

In [ ]:
```
scores_NOD_reduced = show_scores(ideal_model_rf_NOD_reduced, X_train_NOD_reduced,
                                 X_test_NOD_reduced, y_train_NOD, y_test_NOD)
scores_NOD_reduced
```

Out[ ]:
```
{'Train MSE': 27742.26486554887,
 'Test MSE': 71455.57170582094,
 'Train RMSE': 166.56009385668847,
 'Test RMSE': 267.3117500332167,
 'Train R^2': 0.9813844232605544,
 'Test R^2': 0.9508738808270876}
```

Plot model accuracy and RMSE for full dataset vs reduced dataset

In [ ]:
```
ideal_scores_NOD
```

Out[ ]:
```
{'Train MSE': 30918.26552343868,
 'Test MSE': 83421.34959151586,
 'Train RMSE': 175.83590510313496,
 'Test RMSE': 288.8275429932468,
 'Train R^2': 0.9792532676300386,
 'Test R^2': 0.9426473392659994}
```

In [ ]:
```
# Create dataframe with scores from full dataset
ideal_scores_NOD_df = pd.DataFrame.from_dict(ideal_scores_NOD, orient = 'index', co
ideal_scores_NOD_df.reset_index(drop = False, inplace = True)
ideal_scores_NOD_df = ideal_scores_NOD_df.rename(columns = {'index':'Metric'})
ideal_scores_NOD_df.drop([ideal_scores_NOD_df.index[0], ideal_scores_NOD_df.index[
ideal_scores_NOD_df.reset_index(drop = True, inplace = True)
```

In [ ]:
```
ideal_scores_NOD_df
```

Out[ ]:

	Metric	Value
**0**	Train RMSE	175.835905
**1**	Test RMSE	288.827543
**2**	Train R^2	0.979253
**3**	Test R^2	0.942647

In [ ]:
```
# Create dataframe with scores from reduced dataset
reduced_scores_NOD_df = pd.DataFrame.from_dict(scores_NOD_reduced, orient = 'index
reduced_scores_NOD_df.reset_index(drop = False, inplace = True)
reduced_scores_NOD_df = reduced_scores_NOD_df.rename(columns = {'index':'Metric'})
reduced_scores_NOD_df.drop([reduced_scores_NOD_df.index[0], reduced_scores_NOD_df.i
reduced_scores_NOD_df.reset_index(drop = True, inplace = True)
```

In [ ]:
```
reduced_scores_NOD_df
```

Out[ ]:

	Metric	Value
**0**	Train RMSE	166.560094
**1**	Test RMSE	267.311750
**2**	Train R^2	0.981384
**3**	Test R^2	0.950874

In [ ]:
```
# Concatenate dataframes
concat_scores_NOD = pd.concat([ideal_scores_NOD_df, reduced_scores_NOD_df])
concat_scores_NOD.reset_index(drop=True, inplace = True)

# Separate dataframe in two and add column based on Full or Reduced dataset
concat_scores_NOD_rmse = concat_scores_NOD.loc[concat_scores_NOD['Metric'].isin(['
```

```
concat_scores_NOD_rmse.reset_index(drop=True, inplace = True)
concat_scores_NOD_rmse['Dataset'] = ['Full', 'Full', 'Reduced', 'Reduced']

concat_scores_NOD_r2 = concat_scores_NOD.loc[concat_scores_NOD['Metric'].isin(['Tra
concat_scores_NOD_r2.reset_index(drop=True, inplace = True)
concat_scores_NOD_r2['Dataset'] = ['Full', 'Full', 'Reduced', 'Reduced']
```

Figure S8

In [ ]:
```
# Plot the RMSE and R2 for both datasets
fig, axes = plt.subplots(nrows = 1, ncols = 2, figsize=(9,3.5))

g1 = sns.barplot(x = 'Metric', y = 'Value', palette = 'Blues_d', hue = 'Dataset',
                 data = concat_scores_NOD_rmse, ax = axes[0])
g2 = sns.barplot(x = 'Metric', y = 'Value', palette = 'Blues_d', hue = 'Dataset',
                 data = concat_scores_NOD_r2, ax = axes[1])

### Customize the axes[0] plot
# Customize the RMSE plot
axes[0].set_xlabel('', fontsize = 20)
axes[0].set_ylabel('Value', fontsize = 20)
axes[0].tick_params(labelsize = 18)
axes[0].legend(prop={"size":16}, loc = 0)

### Customize the axes[1] plot
# Customize the R2 plot
axes[1].set_xlabel('', fontsize = 20)
axes[1].set_ylabel('Value', fontsize = 20)
axes[1].tick_params(labelsize = 18)
axes[1].legend(prop={"size":16}, loc = 4)

for p in g1.patches:
    g1.annotate(str(round(p.get_height(),2)), (p.get_x() + 0.06, p.get_height() - 
                fontsize = 18, color = 'white')

for p in g2.patches:
    g2.annotate(str(round(p.get_height(),2)), (p.get_x() + 0.12, p.get_height() - 
                fontsize = 18, color = 'white')

# Figure labels
fig.text(-0.003, 1.35, '(a)', va='center', rotation='horizontal', fontsize = 20)
fig.text(0.705, 1.35, '(b)', va='center', rotation='horizontal', fontsize = 20)

x_offset = 0.00
y_offset = 0.00

# set the spacing between subplots
plt.subplots_adjust(left=0.0,
                    bottom=0.0,
                    right=0.9,
                    top=0.9,
                    wspace=1,
                    hspace=0.5)

plt.tight_layout(rect=(0, 0, 1.4, 1.4));
```

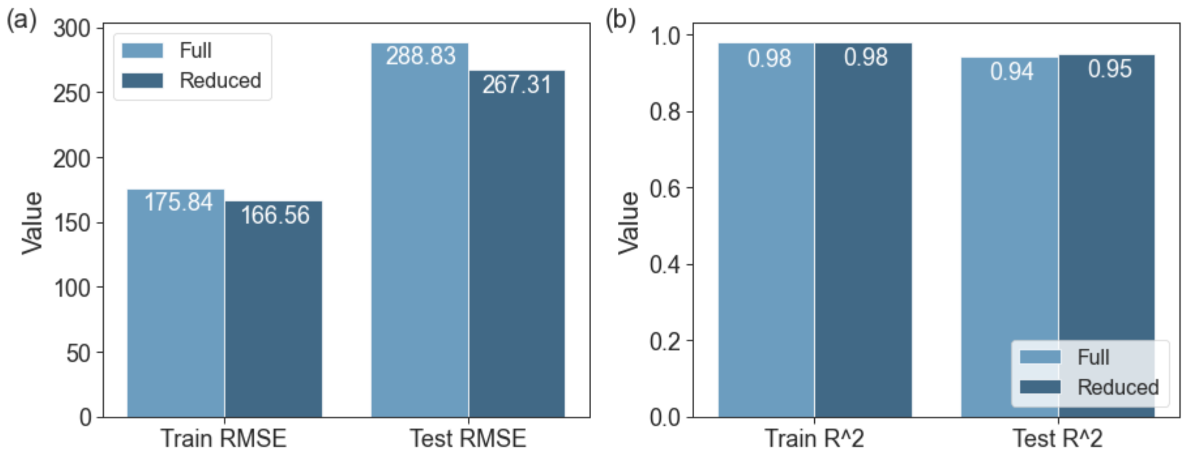

Predictions of the reduced data considering the feature importance for the Nº of drops model

In [ ]:

```
# Make predictions on test data and save them
y_preds_rf_NOD_reduced = ideal_model_rf_NOD_reduced.predict(X_test_NOD_reduced)

# View predictions
y_preds_rf_NOD_reduced
```

Out[ ]:

```
array([4476.66910198, 5566.44880174, 5000.        , 6666.66666667,
       5000.        , 5305.55555556, 3460.20761246, 3906.25      ,
       6655.41031227, 7172.06790123, 4968.19347268, 6959.64960058,
       6250.        , 3460.20761246, 3906.25      , 6959.64960058,
       4557.07490628, 6688.27160494, 5095.51331054, 3906.25      ,
       5298.70922728, 3906.25      , 3640.57239057, 6208.33333333,
       6492.58107802, 6250.        , 5179.78950127, 5285.74704736,
       4444.44444444, 4215.56170167, 3460.20761246, 3906.25      ,
       3640.57239057, 5190.1018404 , 5882.35294118, 6666.66666667,
       5561.00217865, 3380.44460208, 3460.20761246, 3924.18981481,
       3906.25      , 5000.        , 3460.20761246, 6666.66666667,
       5840.85635096, 3640.57239057, 5000.        ])
```

Figure 4b

In [ ]:

```
# Plot Y-train vs Y-preds for RFR
fig, ax = plt.subplots(figsize=(9, 7))

g1 = sns.regplot(x = y_test_NOD,
            y= y_preds_rf_NOD, scatter_kws={"color":"darkred","alpha":0.3,"s":100}
                line_kws={"color":"darkred","alpha":0.5,"lw":3}, label = 'All var
g2 = sns.regplot(x = y_test_NOD,
            y= y_preds_rf_NOD_reduced, scatter_kws={"color":"steelblue","alpha":0.
                line_kws={"color":"steelblue","alpha":0.5,"lw":3}, label = 'Impor

ax.tick_params(labelsize=20)
ax.set_xlabel("Experimental Nº of drops",fontsize=24)
ax.set_ylabel("Predicted Nº of drops",fontsize=24)
plt.legend(prop={"size":16}, loc = 4)

plt.xticks(np.arange(3000,8100, 750))
plt.yticks(np.arange(3000,8100, 750))
plt.xlim([3000, 7750])
plt.ylim([3000, 7750])
```

Out[ ]: (3000.0, 7750.0)

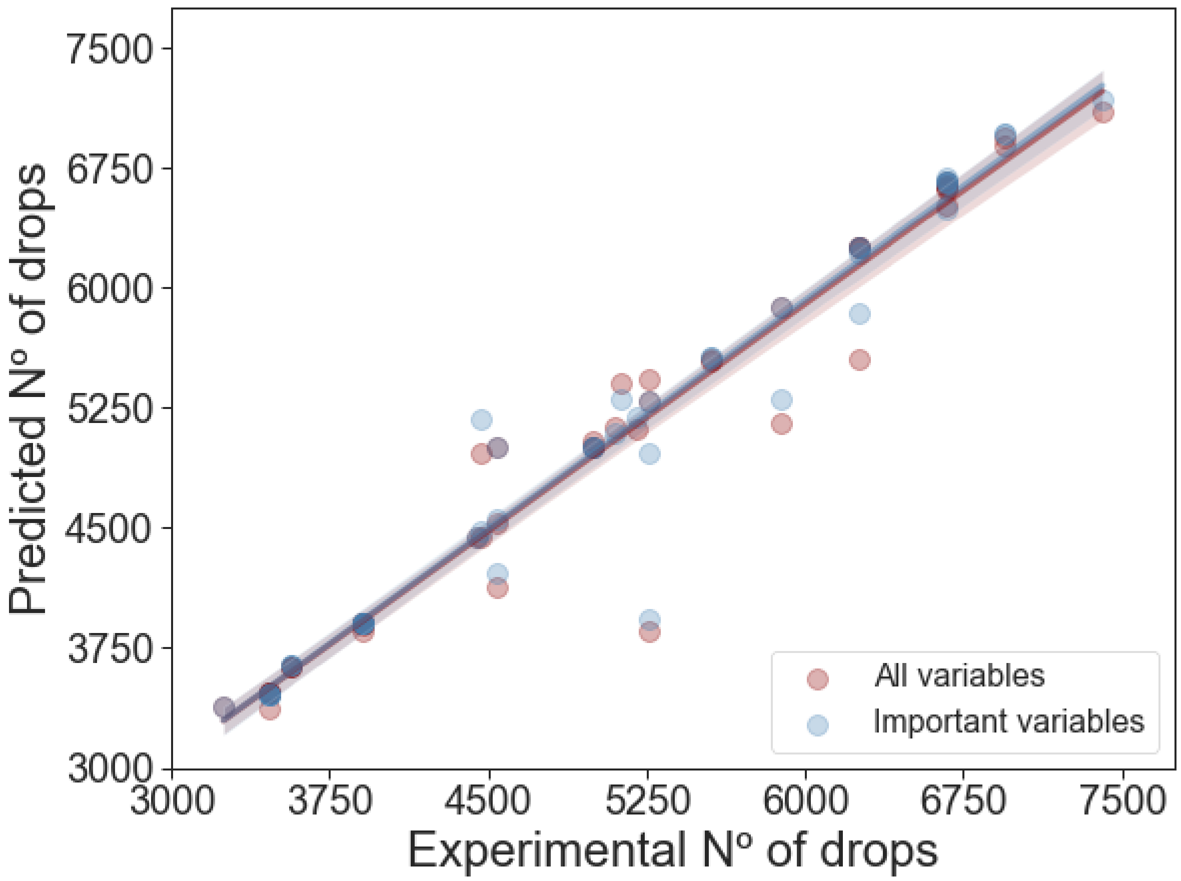

Individual SHAP Value Plot — Local Interpretability

Individual predictions will be made based on the model results and using the SHAP library, which will also allow knowing which input parameters would be required to obtain the predicted N° of drops.

Check https://github.com/slundberg/shap for details on the implementation of the SHAP library.

In [ ]:
```
# Get the predictions and put them with the test data.
X_output = X_test_NOD_reduced.copy()
X_output.loc[:,'predict'] = np.round(ideal_model_rf_NOD_reduced.predict(X_output),:

# Randomly pick some observations (all)
random_picks = np.arange(1,30,1) # Every 1 rows
S = X_output.iloc[random_picks]
S.rename(columns = {'predict': 'Predicted Nº of drops'}, inplace = True)
S
```

Out[ ]:

	Average Drop Volume (pl)	Drop Pitch (um)	Line Pitch (um)	Predicted Nº of drops
**66**	197.5	50	90.0	5566.45
**9**	222.5	50	110.0	5000.00
**170**	183.0	50	75.0	6666.67
**15**	210.0	50	100.0	5000.00
**200**	190.0	85	50.0	5305.56
**25**	215.0	85	85.0	3460.21
**196**	212.5	80	80.0	3906.25
**154**	187.0	125	30.0	6655.41
**126**	180.0	45	75.0	7172.07
**215**	190.0	190	25.0	4968.19
**19**	171.5	45	80.0	6959.65
**96**	202.5	50	80.0	6250.00
**178**	212.5	85	85.0	3460.21
**175**	215.0	160	40.0	3906.25
**137**	170.0	45	80.0	6959.65
**146**	200.0	220	25.0	4557.07
**30**	180.0	125	30.0	6688.27
**226**	190.0	245	20.0	5095.51
**185**	220.0	80	80.0	3906.25
**108**	180.0	75	65.0	5298.71
**162**	212.5	80	80.0	3906.25
**205**	225.0	200	35.0	3640.57
**181**	195.0	50	80.0	6208.33
**125**	200.0	125	30.0	6492.58
**18**	225.0	50	80.0	6250.00
**147**	180.0	160	35.0	5179.79
**101**	170.0	50	95.0	5285.75
**104**	220.0	75	75.0	4444.44
**84**	220.0	220	25.0	4215.56

In [ ]:

```
X_train_NOD_reduced.mean()
```

Out[ ]:

```
Average Drop Volume (pl)    203.336957
Drop Pitch (um)             100.326087
Line Pitch (um)              67.562500
dtype: float64
```

In [ ]:

```
y_train_NOD.mean()
```

Out[ ]:

```
4976.5952253347
```

In [ ]:
```
# Function to calculate SHAP force plots based on the predictions on the test set
def shap_plot(j):
    explainerModel = shap.TreeExplainer(ideal_model_rf_NOD_reduced)
    shap_values_Model = explainerModel.shap_values(S)
    p = shap.force_plot(explainerModel.expected_value, shap_values_Model[j], S.iloc
                        matplotlib = False, show = False) # set matplotlib to False

    return(p)
```

Figure 4c

In [ ]:
```
# visualize the first prediction's explanation (use matplotlib=True to avoid Javasc
shap_plot(26)
```

Out[ ]:

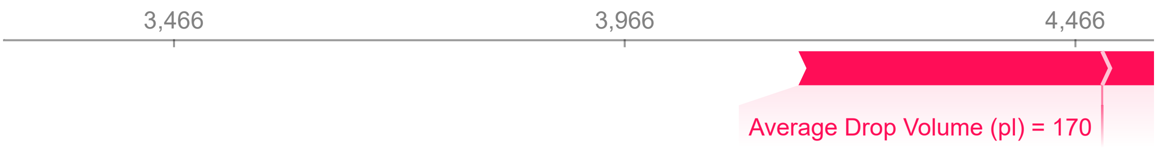

Figure S9

In [ ]:
```
shap_plot(13)
```

Out[ ]:

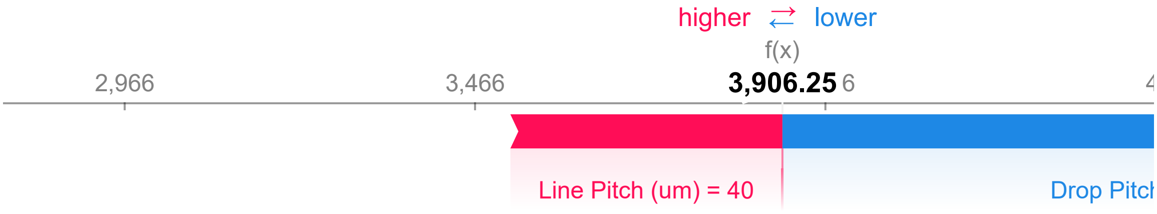

Figure 5

In [ ]:
```
# Represent bubble graph from SHAP predictions
fig, ax = plt.subplots(figsize=(9, 7))

g1 = sns.scatterplot(x = 'Drop Pitch (um)', y = 'Line Pitch (um)', data = S,
                     size = 'Average Drop Volume (pl)', sizes =(100, 1000),
                     hue = 'Predicted Nº of drops', alpha = 0.3)


ax.tick_params(labelsize=20)
ax.set_xlabel(r"Drop Pitch (dx) / $\mu$m",fontsize=24);
ax.set_ylabel(r"Line Pitch (dy) / $\mu$m",fontsize=24);
#ax.legend(fontsize = 14, loc = 1)

# Legend split and place outside
handles, labels = ax.get_legend_handles_labels()

#num_of_colors = (len(S['predict'].unique()) + 1)
h = int(len(handles)/2)
l = int(len(labels)/2)
```

```
color_hl = handles[:h], labels[:l-1]
sizes_hl = handles[h:], labels[l-1:]

# Call legend twice
color_leg = ax.legend(*color_hl,
                      loc = 3, fontsize = 14)
sizes_leg = ax.legend(*sizes_hl,
                      loc = 1, fontsize = 14)

# We need this because the 2nd call to legend() erases the first
ax.add_artist(color_leg)

plt.xticks(np.arange(20, 280, 20));
plt.yticks(np.arange(0,170, 20));
plt.xlim([20,255]);
plt.ylim([-10,120]);
```

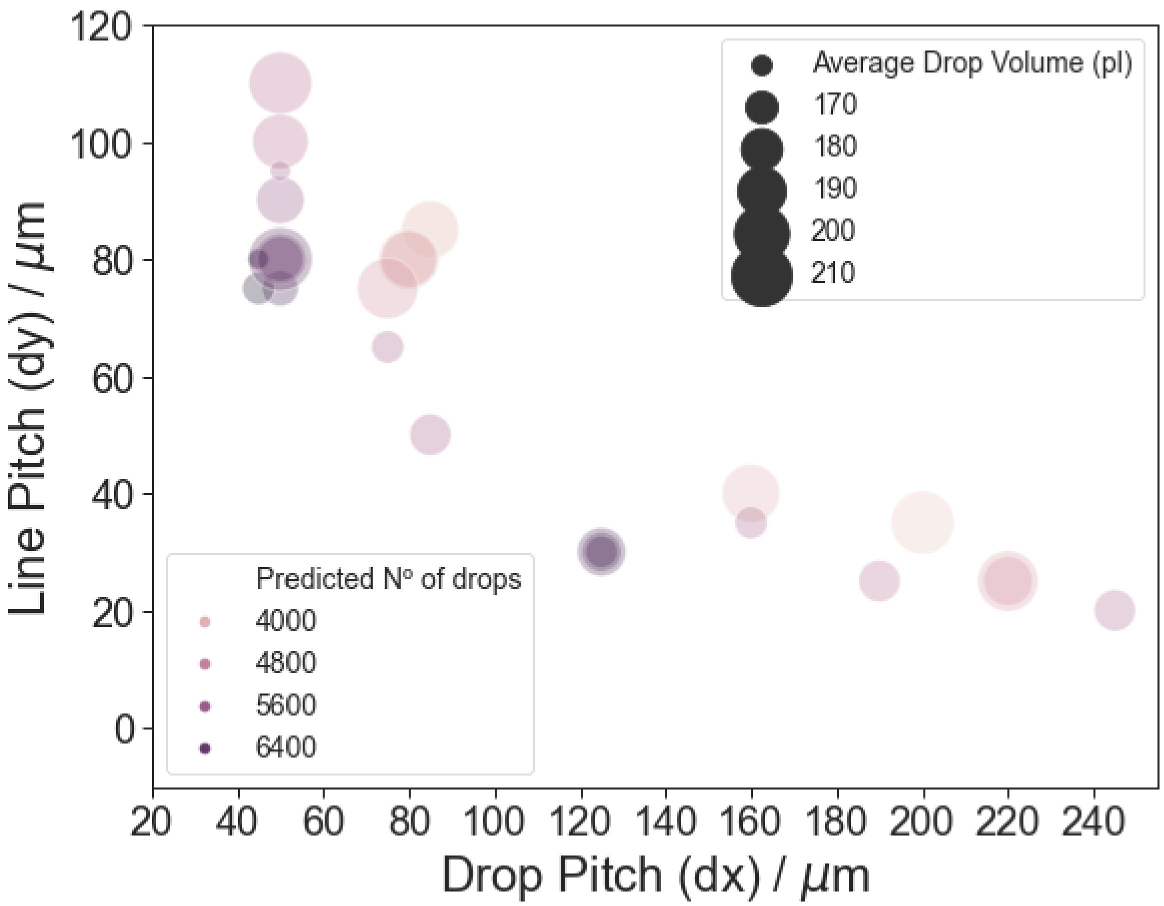

Supplement: TC-010-D1TC05913K-s001 [file TC-010-D1TC05913K-s001.pdf]
