# Supplementary material for: Defining inkjet printing conditions of superconducting cuprate films through machine learning
Source: J Mater Chem C Mater. 2022 Apr 7;10(17):6885–95. doi: 10.1039/d1tc05913k (PMC9069570; doi:10.1039/d1tc05913k)

Notebook description

This notebook presents the Preprocessing and Exploratory Data Analysis of the dataset used to build machine learning models capable of predicting different variables of interest concerning inkjet printing deposition parameters and the resulting films.

Loading the libraries and Preprocessing

Import libraries

Load the libraries necessary to do data preparation and for machine learning.

In [ ]:
```
# EDA and plotting libraries
import numpy as np
import pandas as pd
import matplotlib.pyplot as plt
import matplotlib.gridspec as gridspec
import seaborn as sns
import pandas_profiling

# The Patch library is imported to fix a legend issue, not allowing to show more th
from matplotlib.patches import Patch

# Library to format the axes
import matplotlib.ticker as mtick

# Plots appear in the same notebook cell
%matplotlib inline

# Seaborn style
sns.set_style("ticks", {"xtick.major.size": 8, "ytick.major.size": 8})
sns.set_context("paper")

# Activate latex text rendering for figure labels
from matplotlib import rc
rc('text', usetex=False)

# Function to display dataframes side_by_side
from IPython.display import display
from IPython.display import display_html
def display_side_by_side(*args):
    html_str=''
    for df in args:
        html_str+=df.to_html()
    display_html(html_str.replace('table','table style="display:inline"'),raw=True

# Ignore warning
import warnings
warnings.filterwarnings("ignore")
```

Load data

We will load the excel file. It is necessary to install the openpyxl library, because the "read_excel" function doesn't work for ".xlsx" files without this library.

In [ ]:
```
# Load dataset
data = pd.read_excel("ExpCond_pyrolysis_230samples_article.xlsx", engine='openpyxl
data.shape
```

Out[ ]:
```
(231, 16)
```

Check the dataset

We will check the head and tail of the excel file with the data.

In [ ]:
```
data.head()
```

Out[ ]:

	Nozzle 1	Nozzle 2	Nozzle 1 Voltage (V)	Nozzle 2 Voltage (V)	Average Voltage (V)	Nozzle 1 Pulse Length (us)	Nozzle 2 Pulse Length (us)	Average Pulse Length (us)	Drop Volume Nozzle 1 (pl)	Drop Volume Nozzle 2 (pl)	Aver D Vol
**0**	132	0	135.0	NaN	135.0	26.0	NaN	26.0	NaN	NaN	1
**1**	140	0	110.0	NaN	110.0	23.0	NaN	23.0	NaN	NaN	1
**2**	132	0	135.0	NaN	135.0	26.0	NaN	26.0	NaN	NaN	1
**3**	132	0	145.0	NaN	145.0	27.0	NaN	27.0	NaN	NaN	2
**4**	140	140	140.0	120.0	130.0	26.0	26.0	26.0	210.0	228.0	2

In [ ]:
```
data.tail()
```

Out[ ]:

	Nozzle 1	Nozzle 2	Nozzle 1 Voltage (V)	Nozzle 2 Voltage (V)	Average Voltage (V)	Nozzle 1 Pulse Length (us)	Nozzle 2 Pulse Length (us)	Average Pulse Length (us)	Drop Volume Nozzle 1 (pl)	Drop Volume Nozzle 2 (pl)	A V
**226**	132	140	170.0	140.0	155.0	26.0	26.0	26.0	220.0	230.0	
**227**	132	140	170.0	140.0	155.0	26.0	26.0	26.0	220.0	230.0	
**228**	132	140	170.0	140.0	155.0	26.0	26.0	26.0	220.0	230.0	
**229**	132	140	165.0	142.0	153.5	26.0	25.0	25.5	NaN	NaN	
**230**	132	140	NaN	NaN	NaN	NaN	NaN	NaN	NaN	NaN	

Drop columns

We will drop the columns with redundant information.

In [ ]:
```
# Drop columns
data_final = data.drop(labels=[
                                'Nozzle 1', 'Nozzle 1 Voltage (V)', 'Nozzle 1 Puls
                                'Drop Volume Nozzle 1 (pl)',
                                'Nozzle 2', 'Nozzle 2 Voltage (V)', 'Nozzle 2 Puls
```

```
        'Drop Volume Nozzle 2 (pl)'
    ], axis=1, inplace=False)
```

In [ ]:
```
# Check the dataset again
data_final.head()
```

Out[ ]:

	Average Voltage (V)	Average Pulse Length (us)	Average Drop Volume (pl)	Drop Pitch (um)	Line Pitch (um)	nº of drops	Total Volume Deposited (ul)	Amine (%)
**0**	135.0	26.0	185.0	175	75.0	1904.761905	0.352381	1.14
**1**	110.0	23.0	190.0	200	50.0	2500.000000	0.475000	1.14
**2**	135.0	26.0	190.0	175	50.0	2857.142857	0.542857	1.14
**3**	145.0	27.0	220.0	100	100.0	2500.000000	0.550000	1.14
**4**	130.0	26.0	219.0	95	95.0	2770.083102	0.606648	1.14

Statistics on the dataset

We will check different statistic parameters for the different variables of the dataset.

In [ ]:
```
# Check the statistics of the dataset
data_final.describe()
```

Out[ ]:

	Average Voltage (V)	Average Pulse Length (us)	Average Drop Volume (pl)	Drop Pitch (um)	Line Pitch (um)	nº of drops	Total Volume Deposited (ul)	Ami
**count**	213.000000	213.000000	231.000000	231.000000	231.00000	231.000000	231.000000	231.(
**mean**	149.873239	25.507042	203.259740	101.818182	66.67316	4990.487637	0.999752	1.6
**std**	22.927653	1.134243	19.419277	62.569487	26.21292	1220.740273	0.200036	0.4
**min**	95.000000	20.000000	140.000000	45.000000	20.00000	1904.761905	0.352381	0.(
**25%**	135.000000	25.000000	190.000000	50.000000	40.00000	3906.250000	0.839844	1.1
**50%**	150.000000	25.500000	205.000000	80.000000	80.00000	5000.000000	1.000000	2.(
**75%**	160.000000	26.000000	220.000000	160.000000	85.00000	5882.352941	1.164216	2.(
**max**	234.000000	30.000000	240.000000	250.000000	110.00000	10000.000000	1.800000	2.3

Null values treatment

From the previous representation of the dataset, we can see that there are **null** values present. Therefore, it will be necessary to check which columns have null values and treat them accordingly.

In [ ]:
```
# Check if there are null values and how many for each variable
data_final.isnull().sum()
```

Out[ ]:
```
Average Voltage (V)            18
Average Pulse Length (us)      18
Average Drop Volume (pl)        0
Drop Pitch (um)                 0
Line Pitch (um)                 0
nº of drops                     0
Total Volume Deposited (ul)     0
Amine (%)                       0
dtype: int64
```

Null values were found only in the columns **Average Voltage (V)** and **Average Pulse Length (us)**. We will fill them with the mean value of each column.

In [ ]:
```
# Fill NA values column 'Average Voltage (V)'
data_final['Average Voltage (V)'].fillna((data_final['Average Voltage (V)'].mean()

# Fill NA values column 'Average Pulse Length (us)'
data_final['Average Pulse Length (us)'].fillna((data_final['Average Pulse Length (
                                               inplace = True)
```

We will check the null values again to see the changes.

In [ ]:
```
# Check if there are null values and how many for each variable
data_final.isnull().sum()
```

Out[ ]:
```
Average Voltage (V)            0
Average Pulse Length (us)      0
Average Drop Volume (pl)       0
Drop Pitch (um)                0
Line Pitch (um)                0
nº of drops                    0
Total Volume Deposited (ul)    0
Amine (%)                      0
dtype: int64
```

Save the dataset

We will save the dataset after preprocessing for later use.

In [ ]:
```
# Save to csv
#data_final.to_csv('data_final.csv', index = False)
```

Exploratory Data Analysis (EDA)

EDA will be performed on the variables of the dataset to see their distribution and possible relations to be analyzed later in the models.

In [ ]:
```
# Check the shape of the dataset
data_final.shape
```

Out[ ]:
```
(231, 8)
```

In [ ]:
```
# Check the range of values from all columns
data_final.describe()
```

Out[ ]:

	Average Voltage (V)	Average Pulse Length (us)	Average Drop Volume (pl)	Drop Pitch (um)	Line Pitch (um)	nº of drops	Total Volume Deposited (ul)	Ami
**count**	231.000000	231.000000	231.000000	231.000000	231.00000	231.000000	231.000000	231.(
**mean**	149.873239	25.507042	203.259740	101.818182	66.67316	4990.487637	0.999752	1.6
**std**	22.012208	1.088955	19.419277	62.569487	26.21292	1220.740273	0.200036	0.4
**min**	95.000000	20.000000	140.000000	45.000000	20.00000	1904.761905	0.352381	0.(
**25%**	136.000000	25.000000	190.000000	50.000000	40.00000	3906.250000	0.839844	1.1
**50%**	150.000000	25.507042	205.000000	80.000000	80.00000	5000.000000	1.000000	2.(
**75%**	155.000000	26.000000	220.000000	160.000000	85.00000	5882.352941	1.164216	2.(
**max**	234.000000	30.000000	240.000000	250.000000	110.00000	10000.000000	1.800000	2.3

We can see that the prepared dataset with the reduced dimensionality and newly created variables consists of 231 rows (observations) and 9 columns (variables). We can also see the different statistics for each variable. The representation of each variable will be shown one by one in the following sections.

Distribution of the dataset and description of variables one by one

We will analyze the distribution of the data for each variable one by one. We will plot the data using different types of graphs that allow for better exploration of its distribution. In addition, we will use the `pandas-profiling` library to create a report of the variables in the `data_final` dataset.

In [ ]:

```
# Report for the dataset with pandas-profiling
profile = data_final.profile_report()

profile.to_widgets()
```

```
Summarize dataset: 100%|██████████| 85/85 [00:08<00:00,  9.98it/s, Completed]
Generate report structure: 100%|██████████| 1/1 [00:02<00:00,  2.47s/it]
```

```
VBox(children=(Tab(children=(Tab(children=(GridBox(children=(VBox(children=(Gridsp
ecLayout(children=(HTML(valu…
```

Drop formation variables

We will plot the variables that are related to the formation of the inkjet printing drops such as the **Average Drop Voltage (V)**, **Average Pulse Length (μs)**, and **Amine (%)**. We will also consider the **Average Drop Volume (pl)** in this set of variables since it is highly related with them.

Figure S1 from the Supporting Information

In [ ]:

```
### Box plots

# Define drop formation variables
drop_formation = ['Average Voltage (V)', 'Average Pulse Length (us)', 'Amine (%)',
drop_formation_colors = ['steelblue', 'lightsteelblue', 'lightskyblue', 'skyblue']

# Figure subplots and grid
fig, axs = plt.subplots(nrows = len(drop_formation), figsize=(8,10))

# Loop to generate the figure
for index, column in enumerate(drop_formation):

    # Plot the box plots
    sns.boxplot(x = column, data = data_final, color = drop_formation_colors[index
                ax=axs[index])

    # Customize ticks
    axs[index].tick_params(labelsize = 20)

# Customize label names
axs[0].set_xlabel('Average Voltage (AV) / V', fontsize = 25)
axs[1].set_xlabel(r'Average Pulse Length (APL) / $\mu$s', fontsize = 25)
axs[2].set_xlabel('Amine / %', fontsize = 25)
axs[3].set_xlabel('Average Drop Volume (ADV) / pl', fontsize = 25)

# Show the plot
plt.tight_layout(rect=(0, 0, 1.2, 1.2));
plt.show()
```

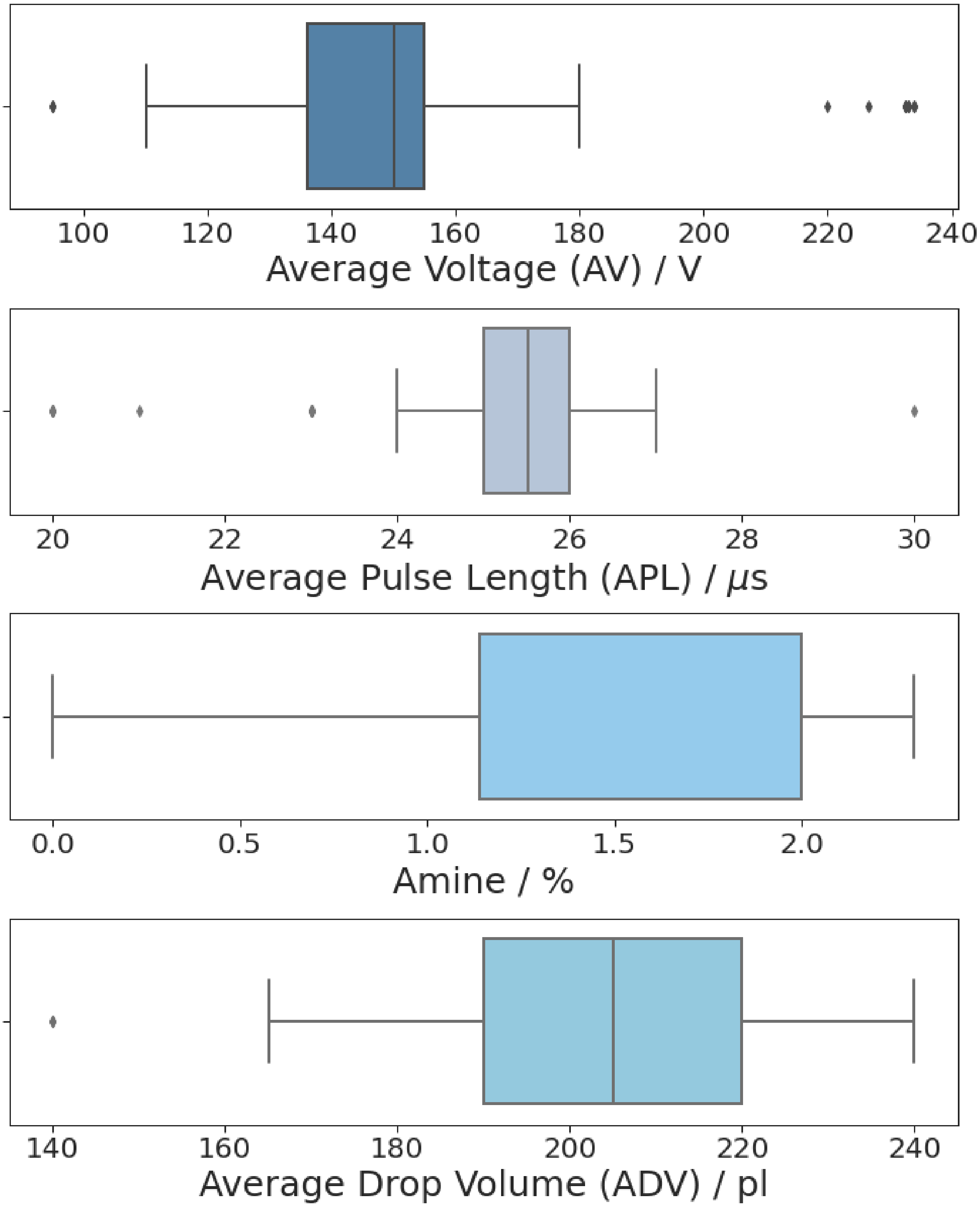

In [ ]:

```
### Histograms

# Define the grid
fig, axes = plt.subplots(nrows = 4, figsize=(8,10))

# Loop to generate the figure
for index, column in enumerate(drop_formation):

    # Plot the histograms

    data_final[column].hist(bins = 40, edgecolor = drop_formation_colors[index], li
                            xlabelsize = 26, ylabelsize = 26, grid = False,
                              color = drop_formation_colors[index], ax = axes[index]

# Customize the ticks, labels and ranges
axes[0].set_xlabel('Average Voltage (AV) / V', fontsize = 25)
axes[0].tick_params(labelsize = 20)
axes[0].set_ylim([0, 65])
```

```
axes[0].set_yticks(np.arange(0, 65, 20))

axes[1].set_xlabel(r'Average Pulse Length (APL) / $\mu$s', fontsize = 25)
axes[1].tick_params(labelsize = 20)
axes[1].set_ylim([0, 90])
axes[1].set_yticks([0, 20, 40, 60, 80])

axes[2].set_xlabel('Amine / %', fontsize = 25)
axes[2].tick_params(labelsize = 20)
axes[2].set_ylim([0, 125])
axes[2].set_yticks([0, 25, 50, 75, 100, 125])

axes[3].set_xlabel('Average Drop Volume (ADV) / pl', fontsize = 25)
axes[3].tick_params(labelsize = 20)
axes[3].set_ylim([0, 35])
axes[3].set_yticks([0, 10, 20, 30])

fig.text(-0.03, 0.65, 'Frequency / Counts', va='center', rotation='vertical', font

# Show the plot
plt.tight_layout(rect=(0, 0, 1.2, 1.2));
plt.show()
```

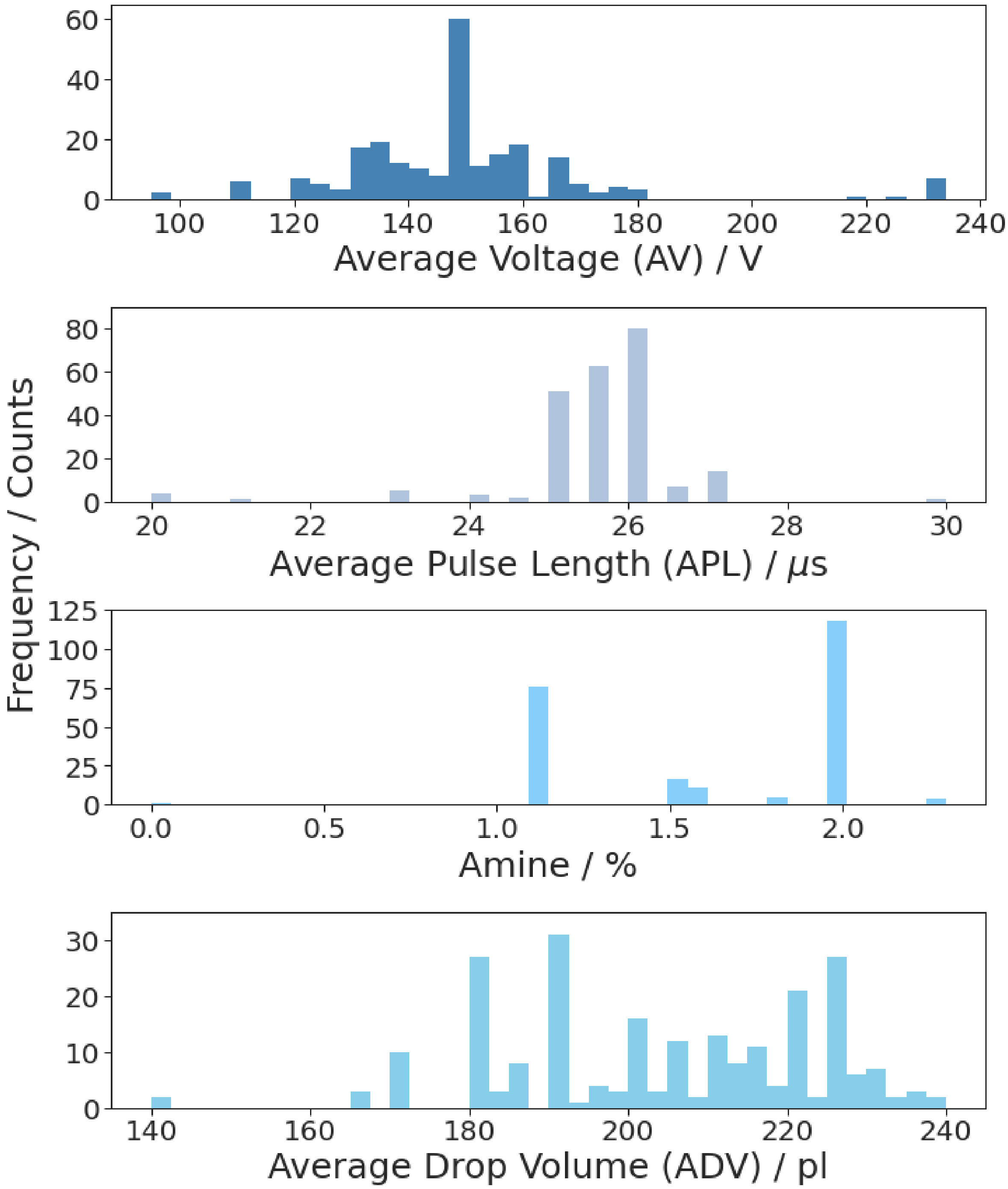

Deposition and film variables

We will plot the variables that are related to the deposition by inkjet printing, i.e., the **Drop Pitch (μm)**, **Line Pitch (μm)**, **nº of drops** and **Total Volume Deposited (μl)**.

Figure S2 from the Supporting Information

In [ ]:
```
### Box plots

# Define deposition variables
deposition = ['Drop Pitch (um)', 'Line Pitch (um)', 'nº of drops', 'Total Volume De
deposition_colors = ['darkred', 'firebrick', 'indianred', 'lightcoral']

# Figure subplots and grid
fig, axs = plt.subplots(nrows = len(deposition), figsize=(8,10))
```

```
# Loop to generate the figure
for index, column in enumerate(deposition):

    # Plot the box plots
    sns.boxplot(x = column, data = data_final, color = deposition_colors[index],
                linewidth = 2.0, ax=axs[index])

    # Customize ticks
    axs[index].tick_params(labelsize = 20)

# Customize the plot
axs[0].set_xlabel(r'Drop Pitch (dx) / $\mu$m', fontsize = 25)
axs[1].set_xlabel(r'Line Pitch (dy) / $\mu$m', fontsize = 25)
axs[2].set_xlabel('Nº of drops (NoD)', fontsize = 25)
axs[3].set_xlabel(r'Total Volume Deposited (TVD) / $\mu$l', fontsize = 25)

# Show the plot
plt.tight_layout(rect=(0, 0, 1.2, 1.2));
plt.show()
```

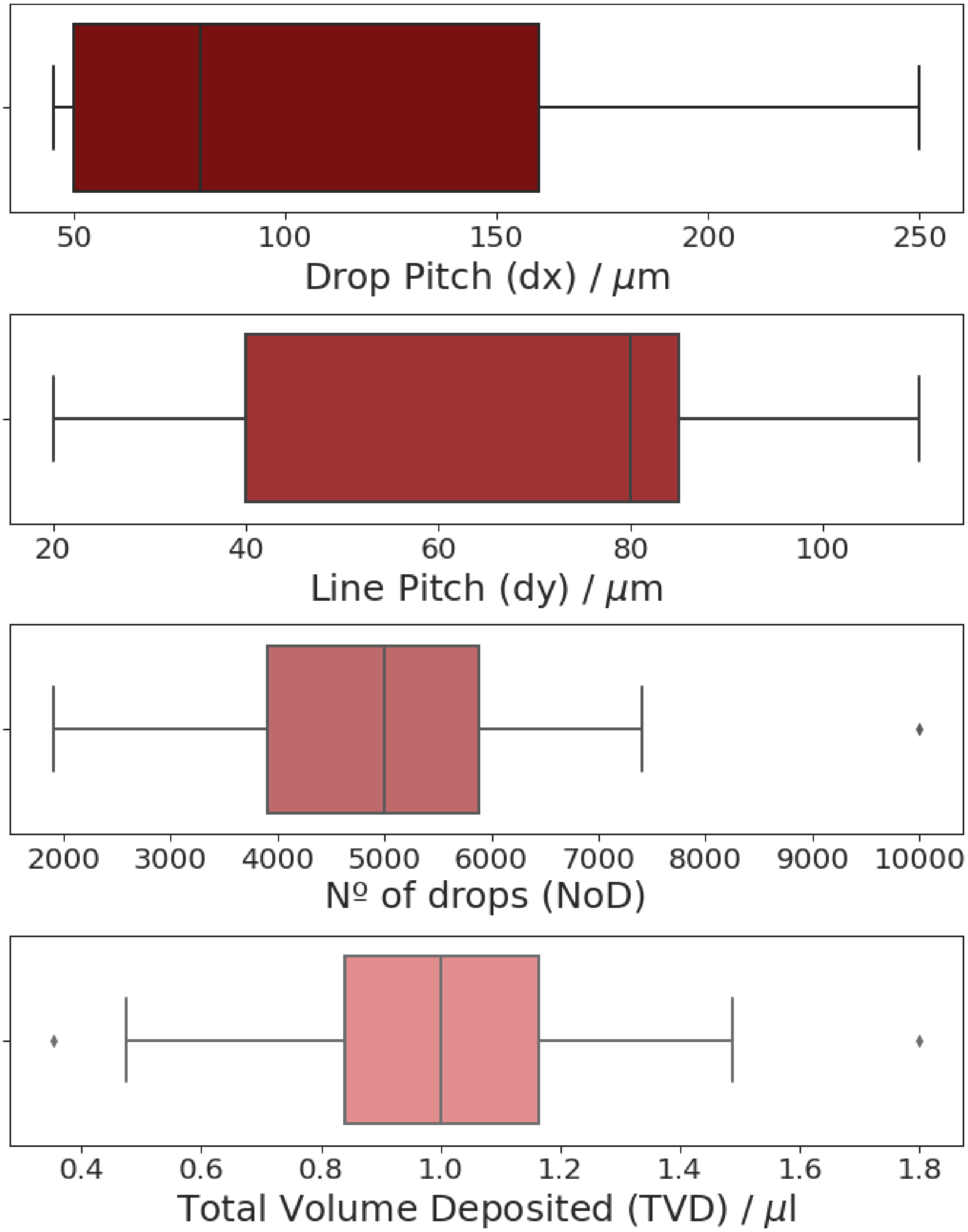

In [ ]:

```
### Histograms

# Define the grid
fig, axes = plt.subplots(nrows = 4, figsize=(8,10))

# Loop to generate the figure
for index, column in enumerate(deposition):

    # Plot the box plots
    sns.boxplot(x = column, data = data_final, color = deposition_colors[index],
                linewidth = 2.0, ax=axs[index])

    data_final[column].hist(bins = 40, edgecolor = deposition_colors[index], linewi
                            xlabelsize = 26, ylabelsize = 26, grid = False,
                              color = deposition_colors[index], ax = axes[index])
```

```
# Customize the plot
axes[0].set_xlabel(r'Drop Pitch (dx) / $\mu$m', fontsize = 25)
axes[0].tick_params(labelsize = 20)
axes[0].set_ylim([0, 100])
axes[0].set_yticks([0, 25, 50, 75, 100])

axes[1].set_xlabel(r'Line Pitch (dy) / $\mu$m', fontsize = 25)
axes[1].tick_params(labelsize = 20)
axes[1].set_ylim([0, 40])
axes[1].set_yticks([0, 10, 20, 30, 40])

axes[2].set_xlabel('Nº of drops (NoD)', fontsize = 25)
axes[2].tick_params(labelsize = 20)
axes[2].set_ylim([0, 45])
axes[2].set_yticks([0, 10, 20, 30, 40])

axes[3].set_xlabel(r'Total Volume Deposited (TVD) / $\mu$l', fontsize = 25)
axes[3].tick_params(labelsize = 20)
axes[3].set_ylim([0, 30])
axes[3].set_yticks([0, 10, 20, 30])

fig.text(-0.025, 0.65, 'Frequency / Counts', va='center', rotation='vertical', fon

# Show the plot
plt.tight_layout(rect=(0, 0, 1.2, 1.2));
plt.show()
```

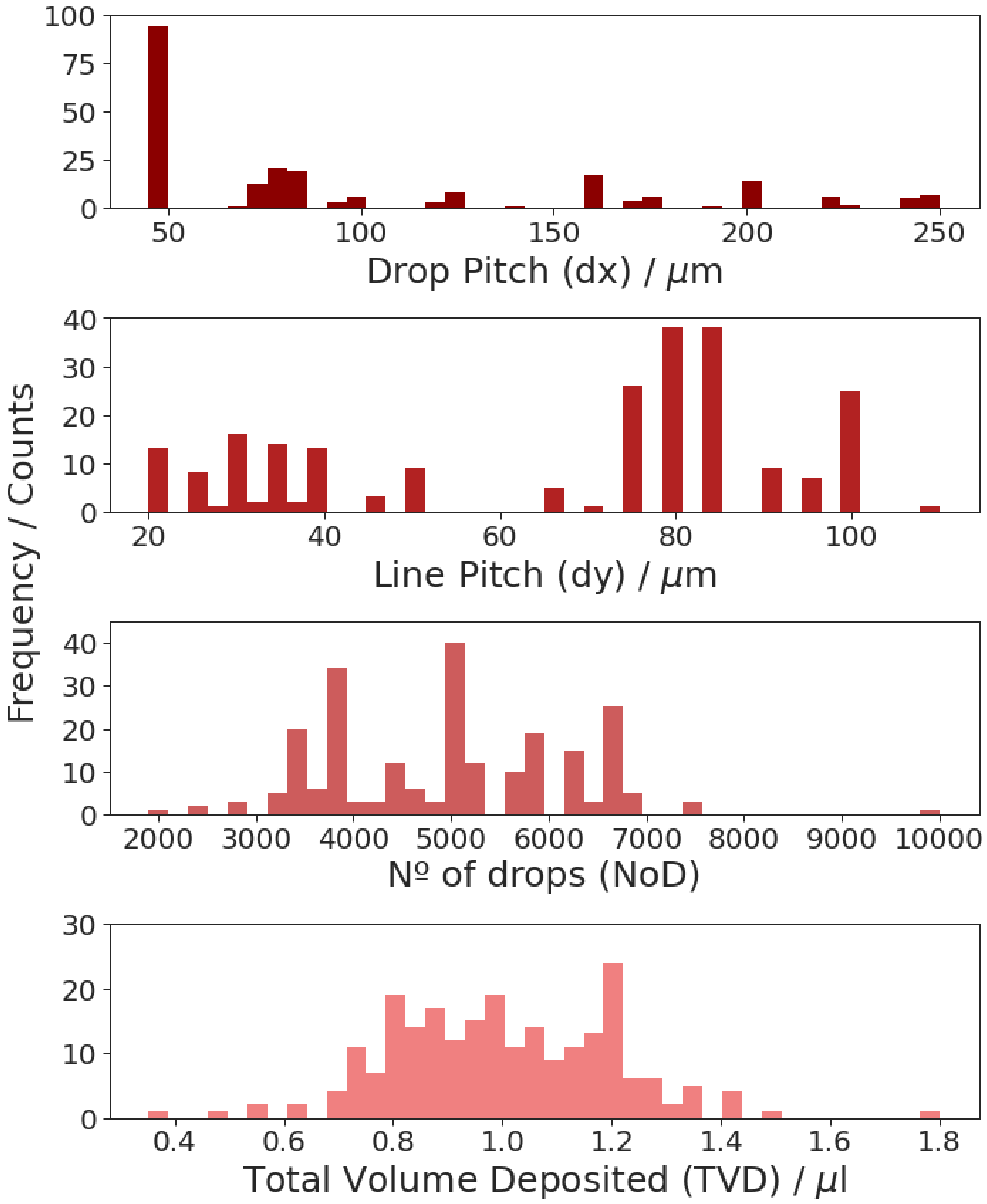

Correlation matrix of the dataset

We will represent the correlation matrix of the dataset with the average variables.

Figure 2

In [ ]:
```
# Correlation matrix of the final dataset after filling NA values and using the ave
corr = round(data_final.corr(), 2)

# Generate a mask for the correlation matrix to show the lower part
corr = corr.where(np.tril(np.ones(corr.shape)).astype(np.bool))

# Plot the correlation matrix
fig, ax = plt.subplots(figsize=(15,10))
ax.tick_params(labelsize=20)
```

```
g = sns.heatmap(corr, annot=True, annot_kws={"size": 20})

# Use matplotlib.colorbar.Colorbar object
cbar = ax.collections[0].colorbar

# Set the labelsize for the color bar
cbar.ax.tick_params(labelsize=20)

var_names = ['Average \nVoltage (AV) / V', r'Average Pulse' '\n' 'Length (APL) / $\
             'Average Drop \nVolume (ADV) / pl', r'Drop Pitch' '\n' '(dx) / $\mu$m
             r'Line Pitch' '\n' '(dy) / $\mu$m', 'Nº of Drops (NoD)',
             r'Total Volume' '\n' 'Deposited (TVD) / $\mu$l', 'Amine / %']

ax.set_xticklabels(var_names)
ax.set_yticklabels(var_names)

# Show the plot
plt.show()
```

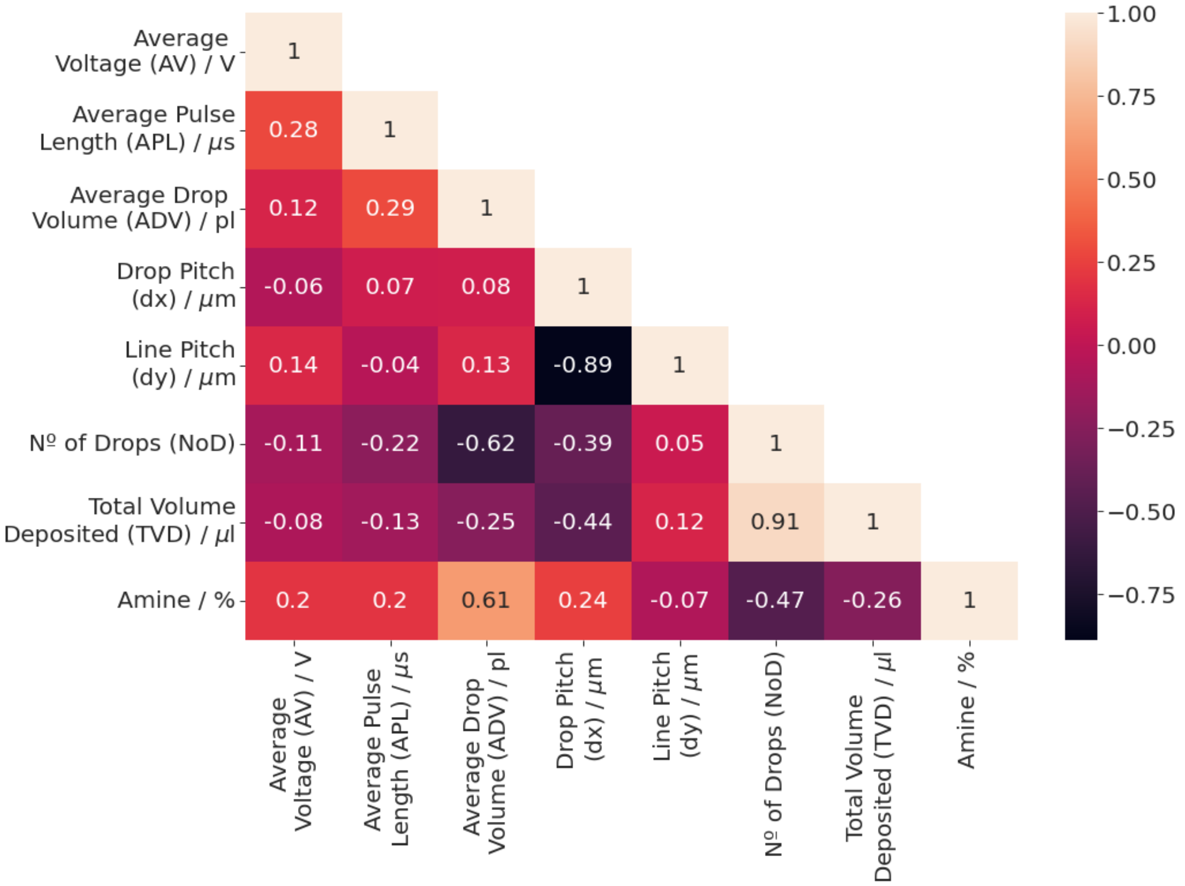

Scatter plots and linear regressions

We will represent the scatter plots and the linear regressions between variables.

Figure S3: Plots for the drop formation variables

In [ ]:
```
### Linear relations of the drop formation variables

# Define the grid
fig, axs_lr_adv = plt.subplots(nrows = 2, ncols = 2, figsize=(8,6))

sns.regplot(x = "Average Voltage (V)", y = "Average Drop Volume (pl)",
            data = data_final, scatter_kws={"color":"steelblue","alpha":0.3,"s":10(
```

```
            line_kws={"color":"steelblue","alpha":0.7,"lw":2}, ax = axs_lr_adv[0][(

sns.regplot(x = "Average Pulse Length (us)", y = "Average Drop Volume (pl)",
            data = data_final, scatter_kws={"color":"lightsteelblue","alpha":0.3,"s
            line_kws={"color":"lightsteelblue","alpha":0.7,"lw":2}, ax = axs_lr_adv

sns.regplot(x = "Amine (%)", y = "Average Drop Volume (pl)",
            data = data_final, scatter_kws={"color":"skyblue","alpha":0.3,"s":100}
            line_kws={"color":"skyblue","alpha":0.7,"lw":2}, ax = axs_lr_adv[1][0]

sns.regplot(x = "Average Voltage (V)", y = "Amine (%)",
            data = data_final, scatter_kws={"color":"steelblue","alpha":0.3,"s":10(
            line_kws={"color":"steelblue","alpha":0.7,"lw":2}, ax = axs_lr_adv[1][1

## Customize the plot
# ADV vs V
axs_lr_adv[0][0].set_xlabel('Average Voltage (AV) / V', fontsize = 20)
axs_lr_adv[0][0].set_ylabel('Average Drop \nVolume (ADV) / pl', fontsize = 20)
axs_lr_adv[0][0].tick_params(labelsize = 18)
axs_lr_adv[0][0].set_xlim([90,240])
axs_lr_adv[0][0].set_ylim([130,260])
axs_lr_adv[0][0].set_xticks(np.arange(100, 250, 20))
axs_lr_adv[0][0].set_yticks(np.arange(130, 260, 20))

# ADV vs PL
axs_lr_adv[0][1].set_xlabel(r'Average Pulse Length (APL) / $\mu$s', fontsize = 20)
axs_lr_adv[0][1].set_ylabel('Average Drop \nVolume (ADV) / pl', fontsize = 20)
axs_lr_adv[0][1].tick_params(labelsize = 18)
axs_lr_adv[0][1].set_xlim([19,31])
axs_lr_adv[0][1].set_ylim([130,260])
axs_lr_adv[0][1].set_xticks(np.arange(20, 31, 2))
axs_lr_adv[0][1].set_yticks(np.arange(130, 260, 20))

# ADV vs EA
axs_lr_adv[1][0].set_xlabel('Amine / %', fontsize = 20)
axs_lr_adv[1][0].set_ylabel('Average Drop \nVolume (ADV) / pl', fontsize = 20)
axs_lr_adv[1][0].tick_params(labelsize = 18)
axs_lr_adv[1][0].set_xlim([-0.2,2.5])
axs_lr_adv[1][0].set_ylim([130,260])
axs_lr_adv[1][0].set_xticks(np.arange(0, 2.6, 0.5))
axs_lr_adv[1][0].set_yticks(np.arange(130, 260, 20))

# EA vs V
axs_lr_adv[1][1].set_xlabel('Average Voltage (AV) / V', fontsize = 20)
axs_lr_adv[1][1].set_ylabel('Amine / %', fontsize = 20)
axs_lr_adv[1][1].tick_params(labelsize = 18)
axs_lr_adv[1][1].set_xlim([90,240])
axs_lr_adv[1][1].set_ylim([-0.2,2.8])
axs_lr_adv[1][1].set_xticks(np.arange(100, 250, 20))
axs_lr_adv[1][1].set_yticks(np.arange(0, 3, 0.5))

# Figure labels
fig.text(-0.015, 1.35, '(a)', va='center', rotation='horizontal', fontsize = 20)
fig.text(0.67, 1.35, '(b)', va='center', rotation='horizontal', fontsize = 20)
fig.text(-0.015, 0.68, '(c)', va='center', rotation='horizontal', fontsize = 20)
fig.text(0.67, 0.68, '(d)', va='center', rotation='horizontal', fontsize = 20)

# Set the spacing between subplots
plt.subplots_adjust(left=0.1,
                    bottom=0.1,
                    right=0.9,
                    top=0.9,
                    wspace=5,
                    hspace=5)
```

```
# Show the plot
plt.tight_layout(rect=(0, 0, 1.4, 1.4), pad = 2.0);
plt.show()
```

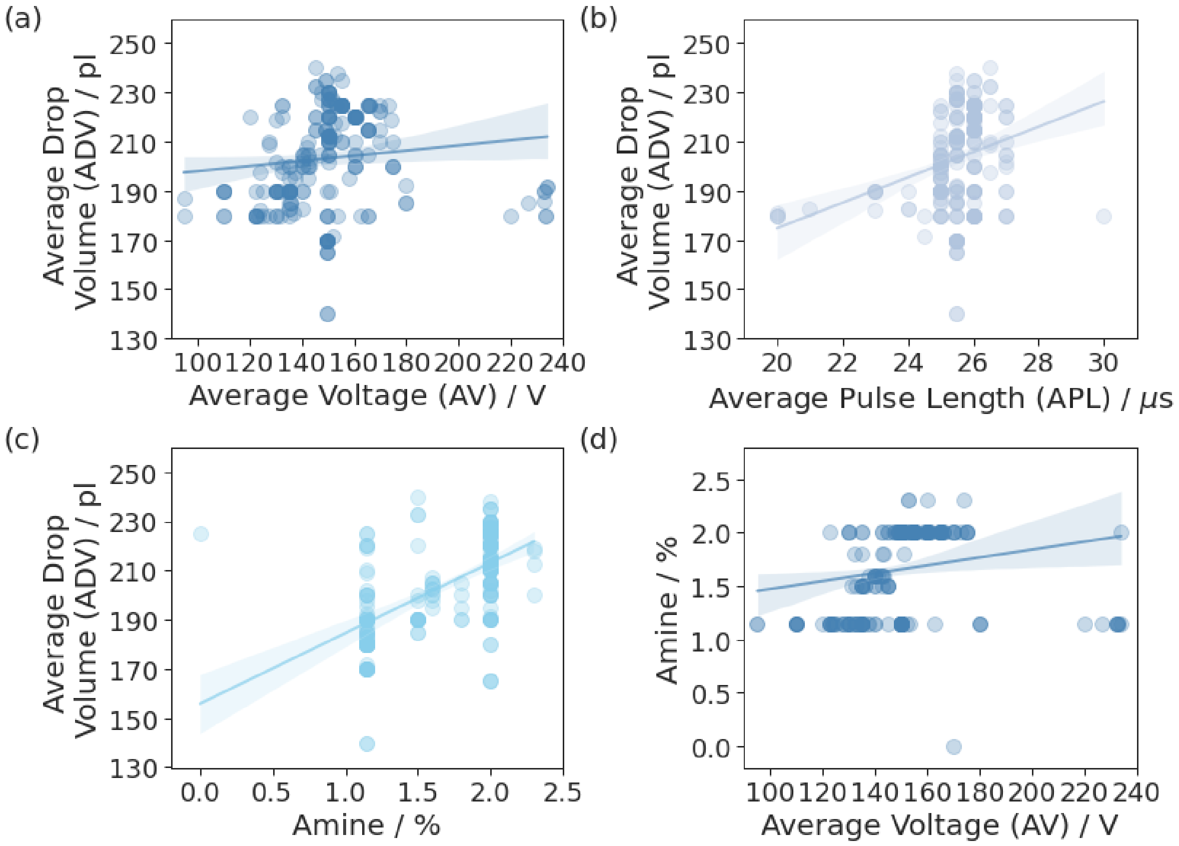

Figure S4: Plot for the Drop and Line Pitch

In [ ]:
```
# Plot Drop pitch vs Line pitch
fig, axs_dp_lp = plt.subplots(nrows = 1, ncols = 1, figsize=(4,3))

sns.regplot(x = "Drop Pitch (um)", y = "Line Pitch (um)",
            data = data_final, scatter_kws={"color":"darkred","alpha":0.3,"s":100},
            line_kws={"color":"darkred","alpha":0.7,"lw":2}, ax = axs_dp_lp);


axs_dp_lp.set_xlabel('Drop pitch (dx) / $\mu$m', fontsize = 20)
axs_dp_lp.set_ylabel('Line pitch (dy) / $\mu$m', fontsize = 20)
axs_dp_lp.tick_params(labelsize = 18)
axs_dp_lp.set_xlim([30,260])
axs_dp_lp.set_ylim([0,120])
axs_dp_lp.set_xticks(np.arange(30, 260, 30))
axs_dp_lp.set_yticks(np.arange(0, 120, 20))

# set the spacing between subplots
plt.subplots_adjust(left=0.1,
                    bottom=0.1,
                    right=0.9,
                    top=0.9,
                    wspace=5,
                    hspace=0.5)

plt.tight_layout(rect=(0, 0, 1.4, 1.4));
```

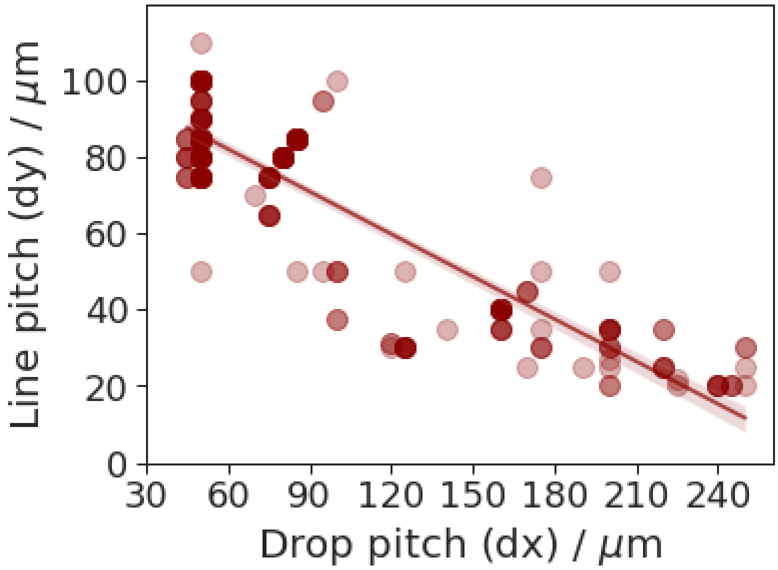

Figure S5: Plot for the deposition variables with the Nº of drops

In [ ]:
```
### Linear relations with the Nº of drops

# Define the grid
fig, axs_lr_drops = plt.subplots(nrows = 2, ncols = 3, figsize=(12,6))

sns.regplot(x = "Drop Pitch (um)", y = "nº of drops",
            data = data_final, scatter_kws={"color":"indianred","alpha":0.3,"s":10
            line_kws={"color":"indianred","alpha":0.7,"lw":2}, ax = axs_lr_drops[0

sns.regplot(x = "Line Pitch (um)", y = "nº of drops",
            data = data_final, scatter_kws={"color":"lightcoral","alpha":0.3,"s":1
            line_kws={"color":"lightcoral","alpha":0.7,"lw":2}, ax = axs_lr_drops[

sns.regplot(x = "Average Voltage (V)", y = "nº of drops",
            data = data_final, scatter_kws={"color":"firebrick","alpha":0.3,"s":10
            line_kws={"color":"firebrick","alpha":0.7,"lw":2}, ax = axs_lr_drops[0

sns.regplot(x = "Average Pulse Length (us)", y = "nº of drops",
            data = data_final, scatter_kws={"color":"indianred","alpha":0.3,"s":10
            line_kws={"color":"indianred","alpha":0.7,"lw":2}, ax = axs_lr_drops[1

sns.regplot(x = "Amine (%)", y = "nº of drops",
            data = data_final, scatter_kws={"color":"lightcoral","alpha":0.3,"s":1
            line_kws={"color":"lightcoral","alpha":0.7,"lw":2}, ax = axs_lr_drops[

sns.regplot(x = "Average Drop Volume (pl)", y = "nº of drops",
            data = data_final, scatter_kws={"color":"firebrick","alpha":0.3,"s":10
            line_kws={"color":"firebrick","alpha":0.7,"lw":2}, ax = axs_lr_drops[1

## Customize the plot
# Drops vs DP
axs_lr_drops[0][0].set_xlabel(r'Drop Pitch (dx) / $\mu$m', fontsize = 20)
axs_lr_drops[0][0].set_ylabel('Nº of drops (NoD)', fontsize = 20)
axs_lr_drops[0][0].tick_params(labelsize = 18)
axs_lr_drops[0][0].set_xlim([30,260])
axs_lr_drops[0][0].set_ylim([1000,10500])
axs_lr_drops[0][0].set_xticks(np.arange(30,260, 30))
axs_lr_drops[0][0].set_yticks(np.arange(1000,10500, 2000))

# Drops vs LP
```

```
axs_lr_drops[0][1].set_xlabel(r'Line Pitch (dy) / $\mu$m', fontsize = 20)
axs_lr_drops[0][1].set_ylabel('Nº of drops (NoD)', fontsize = 20)
axs_lr_drops[0][1].tick_params(labelsize = 18)
axs_lr_drops[0][1].set_xlim([10,120])
axs_lr_drops[0][1].set_ylim([1000,10500])
axs_lr_drops[0][1].set_xticks(np.arange(10, 120, 20))
axs_lr_drops[0][1].set_yticks(np.arange(1000,10500, 2000))

# Drops vs AV
axs_lr_drops[0][2].set_xlabel('Average Voltage (AV) / V', fontsize = 20)
axs_lr_drops[0][2].set_ylabel('Nº of drops (NoD)', fontsize = 20)
axs_lr_drops[0][2].tick_params(labelsize = 18)
axs_lr_drops[0][2].set_xlim([80, 250])
axs_lr_drops[0][2].set_ylim([1000,10500])
axs_lr_drops[0][2].set_xticks(np.arange(80, 250, 20))
axs_lr_drops[0][2].set_yticks(np.arange(1000,10500, 2000))

# Drops vs PL
axs_lr_drops[1][0].set_xlabel(r'Average Pulse Length (APL) / $\mu$s', fontsize = 2(
axs_lr_drops[1][0].set_ylabel('Nº of drops (NoD)', fontsize = 20)
axs_lr_drops[1][0].tick_params(labelsize = 18)
axs_lr_drops[1][0].set_xlim([19,31])
axs_lr_drops[1][0].set_ylim([1000,10500])
axs_lr_drops[1][0].set_xticks(np.arange(19,32,2))
axs_lr_drops[1][0].set_yticks(np.arange(1000,10500, 2000))

# Drops vs EA
axs_lr_drops[1][1].set_xlabel('Amine / %', fontsize = 20)
axs_lr_drops[1][1].set_ylabel('Nº of drops (NoD)', fontsize = 20)
axs_lr_drops[1][1].tick_params(labelsize = 18)
axs_lr_drops[1][1].set_xlim([-0.1, 2.6])
axs_lr_drops[1][1].set_ylim([1000,10500])
axs_lr_drops[1][1].set_xticks(np.arange(0, 2.6, 0.5))
axs_lr_drops[1][1].set_yticks(np.arange(1000,10500, 2000))

# Drops vs ADV
axs_lr_drops[1][2].set_xlabel('Average Drop Volume (ADV) / pl', fontsize = 20)
axs_lr_drops[1][2].set_ylabel('Nº of drops (NoD)', fontsize = 20)
axs_lr_drops[1][2].tick_params(labelsize = 18)
axs_lr_drops[1][2].set_xlim([130, 250])
axs_lr_drops[1][2].set_ylim([1000,10500])
axs_lr_drops[1][2].set_xticks(np.arange(130, 250, 20))
axs_lr_drops[1][2].set_yticks(np.arange(1000,10500, 2000))

# Figure labels
fig.text(0.00, 1.375, '(a)', va='center', rotation='horizontal', fontsize = 20)
fig.text(0.465, 1.375, '(b)', va='center', rotation='horizontal', fontsize = 20)
fig.text(0.931, 1.375, '(c)', va='center', rotation='horizontal', fontsize = 20)
fig.text(0.00, 0.6875, '(d)', va='center', rotation='horizontal', fontsize = 20)
fig.text(0.465, 0.6875, '(e)', va='center', rotation='horizontal', fontsize = 20)
fig.text(0.931, 0.6875, '(f)', va='center', rotation='horizontal', fontsize = 20)

# set the spacing between subplots
plt.subplots_adjust(left=0.1,
                    bottom=0.1,
                    right=0.9,
                    top=0.9,
                    wspace=0,
                    hspace=0.5)

plt.tight_layout(rect=(0, 0, 1.4, 1.4));
```

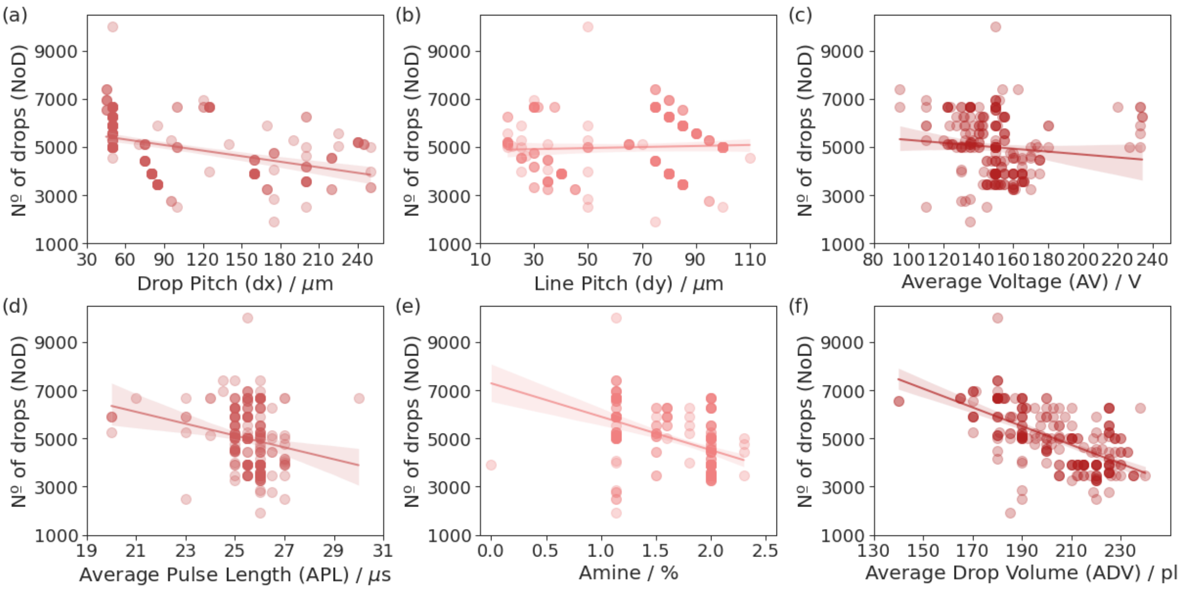

Figure S6: Plot for the deposition variables with the Total Volume Deposited

In [ ]:
```
### Linear relations with the Total Deposited Volume

# Define the grid
fig, axs_lr_tdv = plt.subplots(nrows = 2, ncols = 4, figsize=(16,7))

sns.regplot(x = "nº of drops", y = "Total Volume Deposited (ul)",
            data = data_final, scatter_kws={"color":"mediumseagreen","alpha":0.3,"
            line_kws={"color":"mediumseagreen","alpha":0.7,"lw":2}, ax = axs_lr_td

sns.regplot(x = "Average Drop Volume (pl)", y = "Total Volume Deposited (ul)",
            data = data_final, scatter_kws={"color":"darkgreen","alpha":0.3,"s":10
            line_kws={"color":"darkgreen","alpha":0.7,"lw":2}, ax = axs_lr_tdv[0][

sns.regplot(x = "Average Voltage (V)", y = "Total Volume Deposited (ul)",
            data = data_final, scatter_kws={"color":"darkgreen","alpha":0.3,"s":10
            line_kws={"color":"darkgreen","alpha":0.7,"lw":2}, ax = axs_lr_tdv[0][

sns.regplot(x = "Average Pulse Length (us)", y = "Total Volume Deposited (ul)",
            data = data_final, scatter_kws={"color":"forestgreen","alpha":0.3,"s":
            line_kws={"color":"forestgreen","alpha":0.7,"lw":2}, ax = axs_lr_tdv[0

sns.regplot(x = "Amine (%)", y = "Total Volume Deposited (ul)",
            data = data_final, scatter_kws={"color":"darkseagreen","alpha":0.3,"s"
            line_kws={"color":"darkseagreen","alpha":0.7,"lw":2}, ax = axs_lr_tdv[

sns.regplot(x = "Drop Pitch (um)", y = "Total Volume Deposited (ul)",
            data = data_final, scatter_kws={"color":"forestgreen","alpha":0.3,"s":
            line_kws={"color":"forestgreen","alpha":0.7,"lw":2}, ax = axs_lr_tdv[1

sns.regplot(x = "Line Pitch (um)", y = "Total Volume Deposited (ul)",
            data = data_final, scatter_kws={"color":"darkseagreen","alpha":0.3,"s"
            line_kws={"color":"darkseagreen","alpha":0.7,"lw":2}, ax = axs_lr_tdv[

## Customize the plot
# TDV vs Drops
axs_lr_tdv[0][0].set_xlabel('Nº of drops (NoD)', fontsize = 20)
axs_lr_tdv[0][0].set_ylabel(r'Total Volume' '\n' 'Deposited (TVD) / $\mu$l', fonts
axs_lr_tdv[0][0].tick_params(labelsize = 18)
axs_lr_tdv[0][0].set_xlim([1000,10500])
axs_lr_tdv[0][0].set_ylim([0.2,2])
```

```
axs_lr_tdv[0][0].set_xticks(np.arange(1000,10500, 2000))
axs_lr_tdv[0][0].set_yticks(np.arange(0.2,2.1, 0.2))

# TDV vs ADV
axs_lr_tdv[0][1].set_xlabel('Average Drop Volume (ADV) / pl', fontsize = 20)
axs_lr_tdv[0][1].set_ylabel(r'Total Volume' '\n' 'Deposited (TVD) / $\mu$l', fonts
axs_lr_tdv[0][1].tick_params(labelsize = 18)
axs_lr_tdv[0][1].set_xlim([130, 250])
axs_lr_tdv[0][1].set_ylim([0.2,2])
axs_lr_tdv[0][1].set_xticks(np.arange(130, 250, 20))
axs_lr_tdv[0][1].set_yticks(np.arange(0.2,2.1, 0.2))

# TDV vs AV
axs_lr_tdv[0][2].set_xlabel('Average Voltage (AV) / V', fontsize = 20)
axs_lr_tdv[0][2].set_ylabel(r'Total Volume' '\n' 'Deposited (TVD) / $\mu$l', fonts
axs_lr_tdv[0][2].tick_params(labelsize = 18)
axs_lr_tdv[0][2].set_xlim([80, 250])
axs_lr_tdv[0][2].set_ylim([0.2,2])
axs_lr_tdv[0][2].set_xticks(np.arange(80, 250, 20))
axs_lr_tdv[0][2].set_yticks(np.arange(0.2,2.1, 0.2))

# TDV vs PL
axs_lr_tdv[0][3].set_xlabel(r'Average Pulse Length (APL) / $\mu$s', fontsize = 20)
axs_lr_tdv[0][3].set_ylabel(r'Total Volume' '\n' 'Deposited (TVD) / $\mu$l', fonts
axs_lr_tdv[0][3].tick_params(labelsize = 18)
axs_lr_tdv[0][3].set_xlim([19,31])
axs_lr_tdv[0][3].set_ylim([0.2,2])
axs_lr_tdv[0][3].set_xticks(np.arange(19,32,2))
axs_lr_tdv[0][3].set_yticks(np.arange(0.2,2.1, 0.2))

# TDV vs EA
axs_lr_tdv[1][0].set_xlabel('Amine / %', fontsize = 20)
axs_lr_tdv[1][0].set_ylabel(r'Total Volume' '\n' 'Deposited (TVD) / $\mu$l', fonts
axs_lr_tdv[1][0].tick_params(labelsize = 18)
axs_lr_tdv[1][0].set_xlim([-0.1, 2.6])
axs_lr_tdv[1][0].set_ylim([0.2,2])
axs_lr_tdv[1][0].set_xticks(np.arange(0.2, 2.6, 0.5))
axs_lr_tdv[1][0].set_yticks(np.arange(0.2,2.1, 0.2))

# TDV vs DP
axs_lr_tdv[1][1].set_xlabel(r'Drop Pitch (dx) / $\mu$m', fontsize = 20)
axs_lr_tdv[1][1].set_ylabel(r'Total Volume' '\n' 'Deposited (TVD) / $\mu$l', fonts
axs_lr_tdv[1][1].tick_params(labelsize = 18)
axs_lr_tdv[1][1].set_xlim([30,260])
axs_lr_tdv[1][1].set_ylim([0.2,2])
axs_lr_tdv[1][1].set_xticks(np.arange(30,260, 30))
axs_lr_tdv[1][1].set_yticks(np.arange(0.2,2.1, 0.2))

# TDV vs LP
axs_lr_tdv[1][2].set_xlabel(r'Line Pitch (dy) / $\mu$m', fontsize = 20)
axs_lr_tdv[1][2].set_ylabel(r'Total Volume' '\n' 'Deposited (TVD) / $\mu$l', fonts
axs_lr_tdv[1][2].tick_params(labelsize = 18)
axs_lr_tdv[1][2].set_xlim([10,120])
axs_lr_tdv[1][2].set_ylim([0.2,2])
axs_lr_tdv[1][2].set_xticks(np.arange(10, 120, 20))
axs_lr_tdv[1][2].set_yticks(np.arange(0.2,2.1, 0.2))

# 8th axis off
axs_lr_tdv[-1, -1].axis('off')

# Figure labels
fig.text(0.0, 1.375, '(a)', va='center', rotation='horizontal', fontsize = 20)
fig.text(0.35, 1.375, '(b)', va='center', rotation='horizontal', fontsize = 20)
fig.text(0.70, 1.375, '(c)', va='center', rotation='horizontal', fontsize = 20)
```

```
fig.text(1.05, 1.375, '(d)', va='center', rotation='horizontal', fontsize = 20)

fig.text(0.0, 0.6875, '(e)', va='center', rotation='horizontal', fontsize = 20)
fig.text(0.35, 0.6875, '(f)', va='center', rotation='horizontal', fontsize = 20)
fig.text(0.70, 0.6875, '(g)', va='center', rotation='horizontal', fontsize = 20)

# set the spacing between subplots
plt.subplots_adjust(left=0.0,
                    bottom=0.0,
                    right=0.9,
                    top=0.9,
                    wspace=50,
                    hspace=1)

plt.tight_layout(rect=(0, 0, 1.4, 1.4));
```

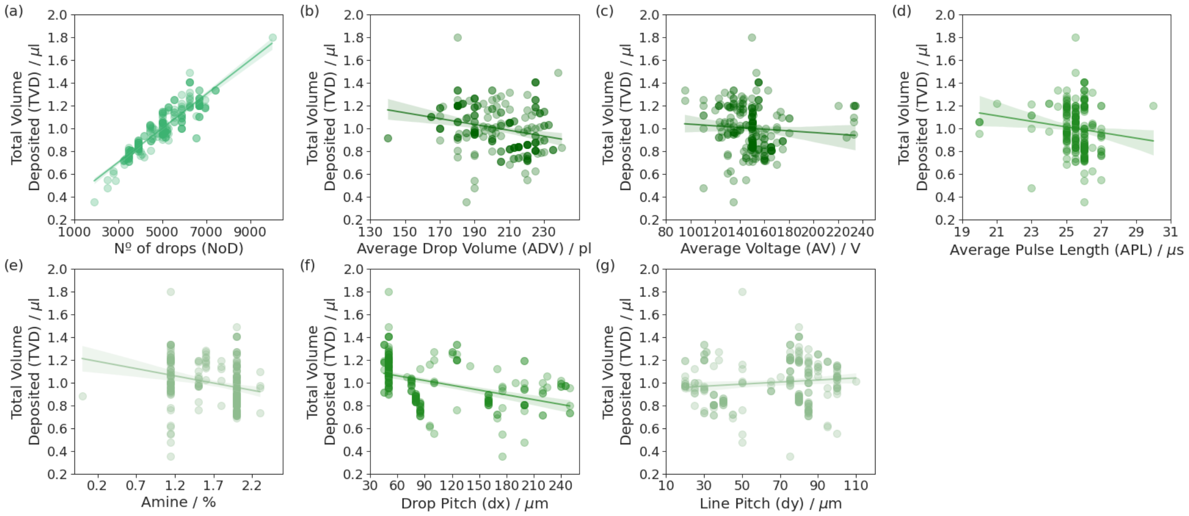

Supplement: TC-010-D1TC05913K-s002 [file TC-010-D1TC05913K-s002.pdf]
