# Supplementary material for: Defining inkjet printing conditions of superconducting cuprate films through machine learning
Source: J Mater Chem C Mater. 2022 Apr 7;10(17):6885–95. doi: 10.1039/d1tc05913k (PMC9069570; doi:10.1039/d1tc05913k)
Supplement: TC-010-D1TC05913K-s003 [file TC-010-D1TC05913K-s003.pdf]

Electronic Supplementary Material (ESI) for Journal of Materials Chemistry C.
This journal is © The Royal Society of Chemistry 2022

Supporting Information

Defining Inkjet Printing Conditions of Superconducting Cuprate Films through Machine Learning

Albert Queraltó, Adrià Pacheco, Nerea Jiménez, Susagna Ricart, Xavier Obradors, Teresa Puig**

Institut de Ciència de Materials de Barcelona (ICMAB-CSIC), Campus UAB, 08193 Bellaterra, Catalonia, Spain

*Corresponding Authors:

Prof. Dr. Teresa Puig

E-mail: teresa.puig@icmab.es

Tel. +34 93 580 18 53

Dr. Albert Queraltó

E-mail: aqueralto@icmab.es

Tel. +34 93 580 18 53

S1. Framework for Machine Learning implementation

Machine learning is a multidisciplinary field comprising elements of mathematics, computer science, artificial intelligence, as well as the discipline of application (materials science in this case).[1] Its goal is to find patterns in existing data through the implementation of a set of tools (e.g. algorithms) to develop models which provide understanding on diverse phenomena and that can be later used to make informed decisions based on data.[2] For example, enabling the selection of best processing parameters to enhance specific physical properties in materials.

The diversity of fields of application for machine learning has led to the definition of a basic framework to implement machine learning tasks that we will describe for our case:[2]

- **Data Collection:** consists of acquiring the data from different experiments, as well as characterization techniques. In our case, it comprises the data from inkjet printing variables defined in the **Methodology section**, as well as the data obtained from optical microscopy images after the deposition that was only used to identify their quality (degree of homogeneity), but not as input in the models. In addition, it must be considered that the range of parameters explored always ensured rather uniform depositions.
- **Data Preparation:** is the process were the collected data is treated to correct errors such as missing values, inconsistencies, combination or creation of variables, etc.[2] In our case, it involved the calculation of the average values for the variables described in the Methodology section and removing of redundant features already included in those variables. We also treated the few null values present in the Average Voltage and Average Pulse Length by filling them with the mean value.
- **Exploratory data analysis:** it is a step performed before building machine learning models and consists in the representation of the distribution of all variables and their relations. This provides

a deep understanding of its characteristics, as well as the relations between the different features to be used in the models. The detailed analysis of the drop formation (AV, APL, Amine and ADV) and deposition (dx, dy, NoD and TVD) features employed in this study is reported in **Section S3**. An initial exploration of the relations between variables is usually performed by building a correlation matrix which represents the linear relationships between variables, two-by-two, based on the Pearson correlation coefficients (ρ_{xy}) which are defined by:[3]

$$\rho_{xy} = \frac{\sigma_{xy}^2}{\sigma_x \sigma_y} \tag{4}$$

where σ_{xy}^2 is the covariance between two variables, and $\sigma_x \sigma_y$ is the product of the standard deviations for each variable. ρ_{xy} indicates the magnitude of the correlation and takes values between -1 and 1. Values above 0 indicate that both variables are positively correlated, i.e., both variables will increase (decrease) together. On the other hand, for negative correlations, one variable raises (shrinks) while the other diminishes (increases). Finally, when $\rho_{xy} = 0$, there is no correlation between variables, and they are independent. Pearson correlation coefficients are used to select the independent variables that will be used in machine learning models by removing highly correlated variables since the information contained is very much the same. This also helps reducing computational costs without affecting much the precision, especially on large datasets.[4]

- **Train/test splitting and cross-validation.** A prior step to the development of machine learning models consists of splitting the data into train and test datasets. This procedure is commonly used in machine learning to evaluate the precision after optimization of model parameters, ensuring that they are able to perform well on unseen data and, thus, prevent overfitting.[5] In first place, we shuffled the data to avoid a bias during splitting due to the way data was introduced in the Excel file used as input. Then, the data was separated into features or attribute variables (*X*) and the target or predicted variable (*Y*). Usually, a feature scaling process is performed by standardizing every variable so that the average value is 0 and the standard deviation is 1. This gives the same

weights to all variables and prevents the predominance of features with larger values on the model. However, feature scaling is only required when algorithms are sensitive to such variations which is not the case of ensemble methods. Therefore, this was not implemented in our workflow, so that we could obtain information on specific values during the interpretation step described later. Anyhow, in the dataset, we provide the same code and models, considering feature scaling to demonstrate that similar results are obtained in terms of precisions and feature importance. Thirdly, the data was randomly split with an 80/20 ratio into train and test datasets, respectively. A random state was used in both the shuffling and train/test splitting steps to ensure reproducibility of model results. The train dataset was divided again, using k-fold cross-validation (CV) into 5 different subsets using the same 80/20 proportion.[6] This allowed us to evaluate and compare the different models considered on the CV datasets, as well as select and optimize the parameters of the most adequate algorithm before conducting a final evaluation on the test dataset.[5]

- **Algorithms selection.** Machine learning algorithms were picked based on the low number of observations from the experimental data. In this sense, we used ensemble methods which work well with small datasets and aim to improve the predictive performance of algorithms, decision trees in our case, by following two different approaches, bagging or boosting, to build our models. On the one hand, we employed the Random Forest (RF) regressor which is based on the bagging approach by building a large amount of decision trees in parallel on random subsamples of the train dataset and averaging the outputs to obtain the final model.[7] On the other hand, the boosting approach (AdaBoost (AB) and Gradient Boosting (GB) regressors)[8,9] consists of the sequential implementation of different decision trees by fitting them iteratively, aiming to minimize the error at each step and obtain a final model with low bias. The basic difference between AB and GB is that the former updates the weights related to each observation of the train dataset at every step, while the latter updates the values of the observations to be used in constructing the next model.[10] Furthermore, the optimization of tree-based models is very simple in comparison to other algorithms due to the low number of parameters to tune. Finally, we compared the precision of the

models developed using ensemble methods with linear regression models which are based on the minimization of the residual sum of squares between the values of the target variable in the dataset, which is used to train the model, and the predicted ones. The implementation of the machine learning algorithms was done with the Scikit-Learn Python library.[11]

- **Model evaluation:** model performance was evaluated against the test set by employing the following metrics:[2]
    - **Coefficient of determination (R^2):** similarly to linear regression, it measures how well a model can predict the dependent variable as a function of the independent ones by comparing with the test values using the following formula:

$$R^2 = 1 - \frac{\sum_{i=0}^{n-1} \left(y_{test,i} - y_{pred,i}\right)^2}{\sum_{i=0}^{n-1} \left(y_{test,i} - \bar{y}_{test,i}\right)^2} \qquad \text{(S1)}$$

where $y_{test,i}$ is the value of the dependent variable in the test set, $\bar{y}_{test,i}$ is the average value of the dependent variable in the test set, $y_{pred,i}$ is the predicted value by the model, and n is the number of samples in the test set. Values close to 1 are indicative of models that perform well on unseen data.

- **Root Mean Squared Error (RMSE):** it calculates the average difference between the predicted and test values for the dependent variable by employing the following equation:

$$RMSE = \sqrt{\frac{1}{n}\sum_{i=0}^{n-1} \left(y_{test,i} - y_{pred,i}\right)^2} \qquad \text{(S2)}$$

From this formula, one can see that the lower the RMSE, the more accurate will be the model in making predictions from the independent variables.

The values of the aforementioned metrics were used to tune model parameters in order to optimize model performance (see **Results and Discussion section**). It is worth noting that better metrics for

the train set as compared to the test set are usually attributed to the model being fitted and optimized on the former. Nevertheless, the closer the metrics for both datasets, the better the model can generalize.

- **Interpretation of model outputs with the SHAP library.** Interpretation of the results obtained from machine learning models is often difficult due to the intricate relations between variables. Ensemble methods are an example of this complexity given that they are a combination of many individual models. The SHAP (Shapley Additive exPlanations) library (https://github.com/slundberg/shap) has been developed with the purpose to provide interpretability to this "black-box" models. The approach consists of transforming a complex machine learning model such as Random Forest into a combination of simple linear models by using *additive feature attribution methods*. This means that each prediction from the original model, $f(x)$, where x is a data point, is converted to an explanatory model, $g(x^{'})$, which is the linear combination of a set of simplified input variables $(x_i^{'})$ in binary form (0=not present, 1=present) by following the transformation:[12,13]

$$f(x) \approx g(x^{'}) = \phi_0 + \sum_i \phi_i x_i^{'} \qquad \text{(S3)}$$

These simplified features are mapped with the original data using a mapping function $x = h_x(x')$. ϕ_0 is the output value with no input variables, and ϕ_i is the effect of each variable i to a prediction, also known as the SHAP value. The SHAP value can be calculated using the Shapley values formula for a model with a prediction function and F variables:[12,13]

$$\phi_i = \sum_{S \subseteq F \setminus \{i\}} \frac{|S|!(|F| - |S| - 1)!}{|F|!} \left[f_{S \cup \{i\}}(x_{S \cup \{i\}}) - f_S(x_S) \right] \qquad \text{(S3)}$$

Where $|F|$ is the total number of variables, S is any subset of variables from F that does not include the i-th element and $|S|$ is the dimensions of the subset. The f functions represent the models trained with the respective features.By adding all ϕ_i, we can obtain the approximate predicted value of the original model, $f(x)$. More details on how these values are calculated can be found on the articles by Lundberg et al.[12–15]

The code and Jupyter Notebooks used for the different steps described above, as well as the figures and models is freely available at http://dx.doi.org/10.20350/digitalCSIC/14016.

S2. Descriptive statistics of the dataset characteristics

The experimental data has been classified in the variables that influence the drop formation which are mainly related to solution and equipment parameters, as well as variables that will define the deposition on the substrates. In this section, we will analyze the characteristics of the different features.

S2a. Analysis of drop formation variables

The features involved in the drop formation are the Average Voltage (*AV*), Average Pulse Length (*APL*) and Amine (**Methodology section** of the main manuscript). We will also analyze the Average Drop Volume (*ADV*) which results from their combination although it could also be considered a deposition variable since it has an impact on its result. The distribution of these attributes was analyzed by representing the data in histograms and box plots (**Figure S1**).

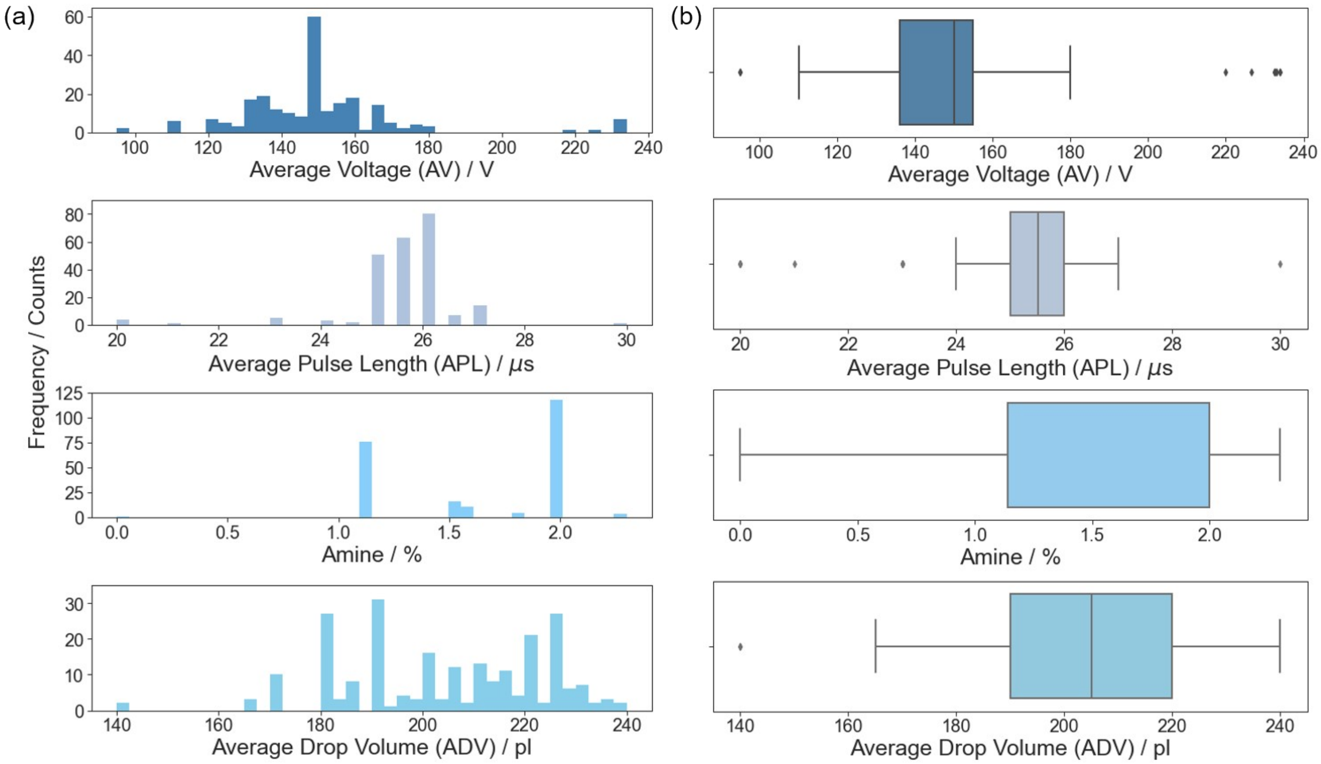

Figure S1. *(a) Histograms, and (b) box plots of the variables Average Voltage (AV), Average Pulse Length (APL), Amine and Average Drop Volume (ADV). The box plots show the 1st, 2nd and 3rd quartiles where most of the values can be found (boxes), the error bars indicate the deviation on each side from the most common values, and the rhombohedra indicate the samples that are considered as outliers.*

The values of *AV* used in the experiments ranged from 95 to 234 V (**Table S1**) and the data follows a rather normal distribution centered around 150 V, the voltage value with a higher number of counts (60 counts). In addition, most of the experimental voltages lay between 136 and 155 V and have 10 to 20 counts (**Figure S1a**). The mean *AV* value, 149.87 ± 22.01 V, is very close to the median, indicating an evenly distributed variable (**Table S1**). The box plot shows that voltages below 100 V, as well as 220 V and above are outliers since they are far and isolated from the main distribution (rhombohedra in **Figure S1b**). Therefore, these values will not be significant in building the machine learning models. In addition, most of the voltages used are in the medium range of the working conditions for the nozzles, since the equipment has a maximum applied voltage of 255 V. It is worth noting that the requirement for such voltages is highly determined by the solution, as well as its concentration and viscosity which are highly

affected by the percentage of amine added to it.[16] Furthermore, the higher voltage values are attributed to a specific nozzle as seen in the raw dataset file (http://dx.doi.org/10.20350/digitalCSIC/14016).

Table S1. Descriptive statistics of the variables involved in the deposition by inkjet printing.

	**Average Voltage (AV) (V)**	**Average Pulse Length (APL) (µs)**	**Amine (%)**	**Average Drop Volume (ADV) (pl)**	**Drop Pitch (dx) (µm)**	**Line Pitch (dy) (µm)**	**Nº of drops (NoD)**	**Total Volume Deposited (TVD) (µl)**
**Mean**	149.87	25.51	1.66	203.26	101.82	66.67	4990.49	0.99
**Standard deviation**	22.93	1.13	0.41	19.42	62.57	26.21	1220.74	0.20
**Minimum**	95	20	0	140	45	20	1904.76	0.35
**1st quartile**	135	25	1.14	190	50	40	3906.25	0.84
**Median**	150	25.5	2	205	80	80	5000	1
**3rd quartile**	160	26	2	220	160	85	5882.35	1.16
**Maximum**	234	30	2.3	240	250	110	10000	1.8

The experimental *APL*s employed are distributed in a range from 20 to 30 µs (**Table S1**), although values between 25 and 26 µs with 50 to 80 counts were mainly used (**Figure S1a**). Few outliers were found below 24 µs, and at 30 µs (**Figure S1b**). This shows that the experimental variation of the *APL* is not very significant in the dataset. Thus, it should have a lower relevance than the *AV* in the drop formation and in the design of machine learning models, given the small change in values.

The percentage of amine was explored within the range from 0 to 2.3 % with a mean value of 1.66 ± 0.41 % (**Table S1**). The data distribution is highly unbalanced since most solutions containing 2 % or 1.14 % amine with 118 and 76 counts, respectively (**Figure S1a**). The remaining percentages employed in the solutions have 10 or less counts. The box plots show that the values of amine employed are in the high range and no outliers are found for this feature (**Figure S1b**). Therefore, it can be expected that the percentage of amine will not have a significant contribution to the models.

Finally, the *ADV* results from the combination of the previous features since they are used experimentally to achieve a stable drop formation as explained in the **Methodology section** of the main manuscript. The experimental distribution of drop volumes ranged from 140 to 240 pl (**Table S1**), although most of them are within 190 and 220 pl with counts between 10 and 30. **Figure S1a** shows that the distribution of values is quite even along the range of *ADVs* recorded. In addition, the box plots indicate that most of the values are in the higher range with an average value of 203.26 ± 19.42 pl, and only a single outlier was observed for an *ADV* of 140 pl (**Figure S1b**).

S2b. Analysis of deposition variables

The variables from the dataset involved in the deposition are the *dx*, *dy*, *NoD* and the *TVD* (**Methodology section** of the main manuscript). The *ADV* could also be considered a deposition variable since it experimentally defines which inkjet printing patterns will be used. **Figure S2** shows the distribution of these variables from histograms and box plots.

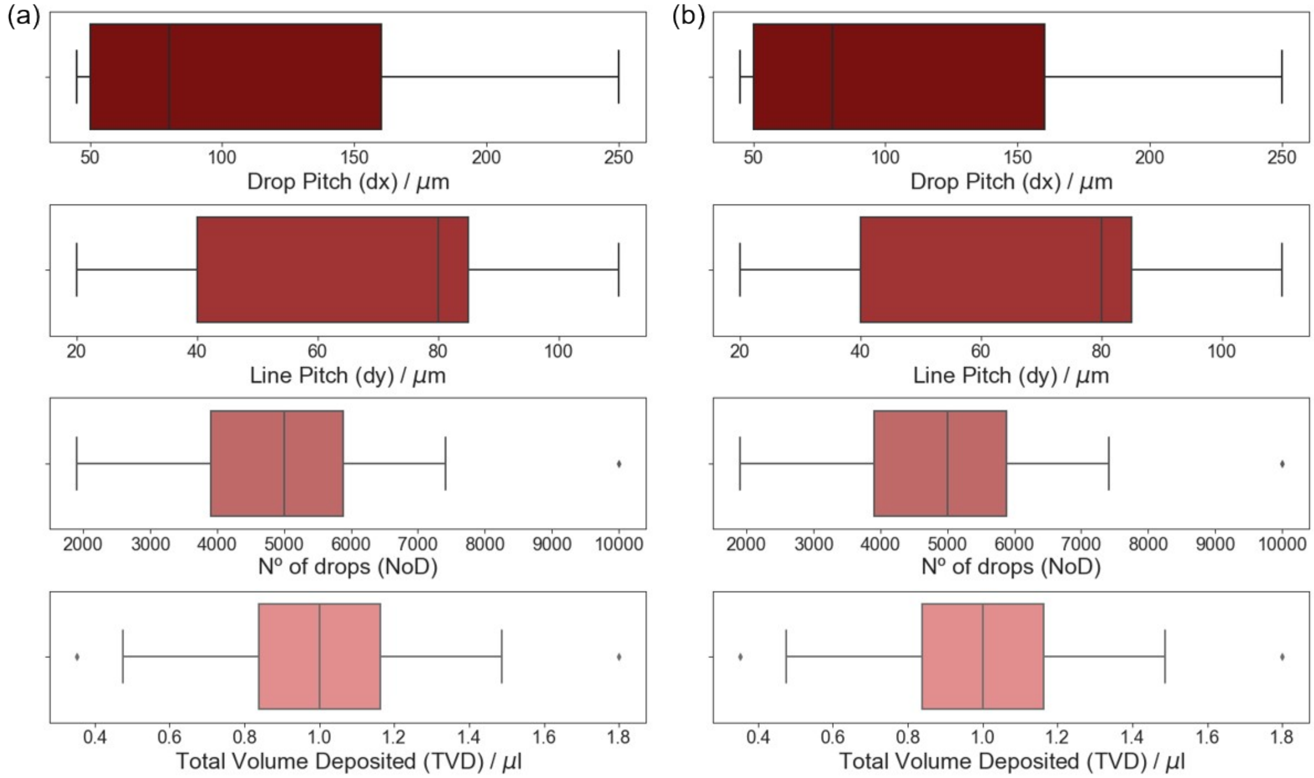

Figure S2. *(a) Histograms, and (b) box plots of the variables Drop pitch (dx), Line pitch (dy), Nº of drops (NoD), and Total Volume Deposited (TVD). The box plots show the 1st, 2nd and 3rd quartiles where most of the values can be found (boxes), the error bars indicate the deviation on each side from the most common values, and the rhombohedra indicate the samples that are considered as outliers.*

The first two features, *dx* and *dy*, define the spacing between inkjet printing drops. On the one hand, the *dx* was experimentally explored in the range from 45 to 250 μm, but most experiments used values within 50 and 160 μm, and the mean *dx* is 101.82 ± 62.57 μm (**Table S1**). We see a rather unbalanced distribution with most observations (84 counts) for a drop pitch of 50 μm (**Figure S2a**), while the rest are all below 25 counts. The box plot in **Figure S2b** shows no outliers and most of the experiments were conducted with a *dx* close to 80 μm. On the other hand, the *dy* was experimentally explored from 20 to 110 μm with a mean value of 66.67 ± 26.21 μm and a median of 80 μm (**Table S1**), but most values can be also found between 75 and 85 μm, and 100 μm with 25 to 40 counts (**Figure S2a**). The remaining values of *dy* are all below 20 counts. The box plot in **Figure S2b** shows no outliers and most of the experiments were

conducted within a small range of *dy* within 40 and 85 μm. The main reason for such distribution of *dx* and *dy* values is caused by the limitations imposed experimentally which are essentially required to obtain a homogeneous deposition without liquid movement, as well as maintain a crack-free film after the pyrolysis step (**Methodology section** of the main manuscript).

The variable *NoD* was calculated by considering the substrate dimensions, as well as *dx* and *dy*, as explained in the **Methodology section** of the main manuscript. The values that this variable takes range from 1905 to 10000 drops (**Table S1**), although the most experimentally used values are 3906 and 5000 with 34 and 29 counts, respectively, followed by values of 5882, 3460, 6667 and 6250 drops with 19, 18, 16 and 15 counts (**Figure S2a**). The remaining values have counts lower than 10. In addition, the mean value is of 4990 ± 1221 drops and the median is 5000 drops, indicating that the variable is evenly distributed (**Table S1**). The box plot in **Figure S2b** shows that one outlier has been measured which corresponds to a value of 10000 drops.

Finally, the *TVD* was calculated by considering the *ADV* and *NoD*, as explained in the **Methodology section** of the main manuscript. This variable follows a rather normal distribution centered around 1 μl and has a mean value of 0.99 ± 0.20 μl (**Table S1**). Although the experimental values range from 0.35 to 1.8 μl, most of them are found within 0.8 and 1.2 μl (**Figure S2a**). Particularly, the most common values are 1.2 μl (11 counts), 0.84 μl (8 counts), 0.8 and 0.86 μl (7 counts), 1 μl (6 counts), and 0.83, 0.87, 0.95 and 1.05 μl (5 counts). The box plot in **Figure S2b** indicates that only two outliers can be found which corresponds to values of 0.34 and 1.8 μl.

S3. Linear relationships between variables

In this section, we present the linear relationships between different features that are considered important in obtaining homogeneous depositions without liquid movement and that will later lead to crack-free films

after the pyrolysis process. **Figure S3a** and **Figure S3b** show scatter plots of the *AV* and *APL* with the *ADV* and their linear regressions. We can see that the data is quite scattered around 136 – 155 V for the former and 25 – 26 μs for the latter, which leads to weak and positive linear relations with correlation coefficients of 0.12 and 0.29 (**Figure 2** in the main manuscript). The color intensity indicates how many instances of the plotted values are present in the data; light and dark color for low and high counts, respectively. **Figure S3c** and **Figure S3d** present the scatter plots and their linear regressions for the *ADV* and *AV* with *Amine*, respectively. The data is highly spread for *Amine* values of 1.14 and 2 %. While both linear relations are positive, the former is stronger than the latter and the correlation coefficients are 0.61 and 0.2, respectively. This indicates a larger contribution of the *Amine* in defining the *ADV*. As mentioned in the main manuscript, this could be caused by the need to raise the *AV* and *APL* due to the increase in viscosity caused by the *Amine*.[16]

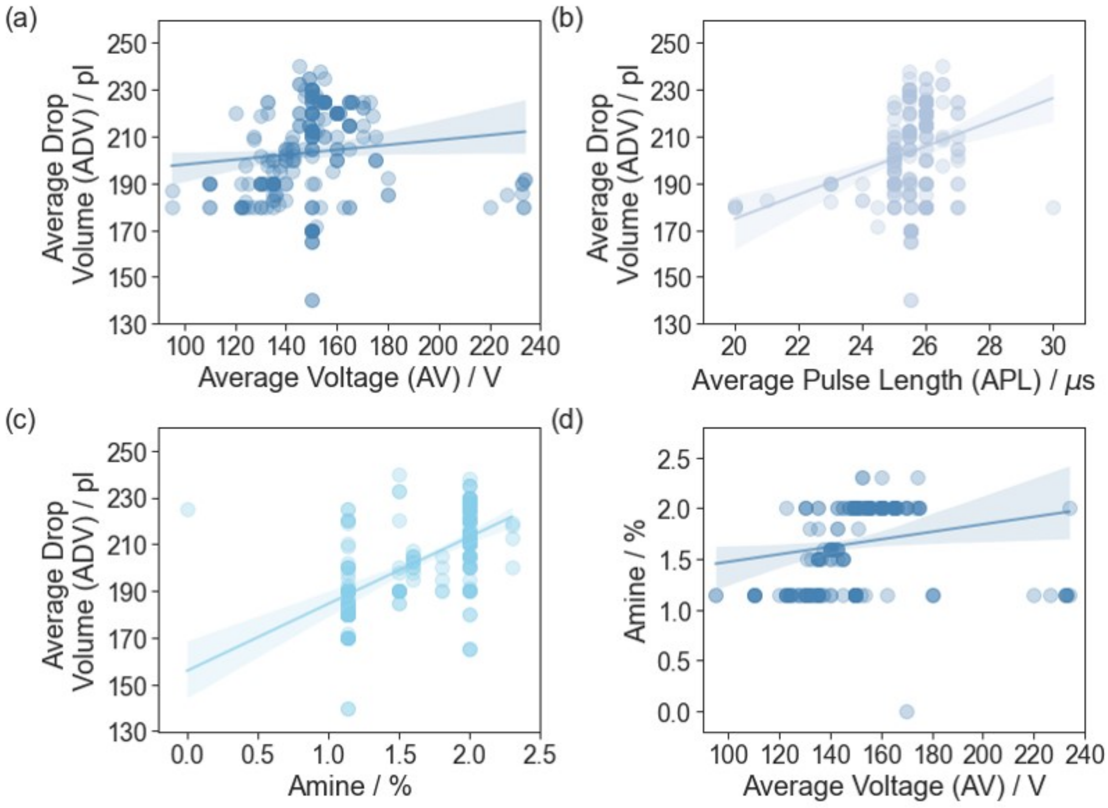

Figure S3. *Scatter plots and linear regressions for the Average Drop Volume (ADV) with variables controlling drop formation: (a) Average Voltage (AV), (b) Average Pulse Length (APL), and (c) Amine. (d) Scatter plot and linear regression between the Average Voltage (AV) and Amine.*

Figure S4 displays the scatter plot between *dx* and *dy* variables that define the IJP grid size that will be printed on the substrates. We can see that there is some scattering around some data points, particularly at a *dx* of 50 µm. In addition, the linear relationship between both variables is strong and negative with a correlation coefficient of -0.89 (**Figure 2**). The reason for this relationship is related to the experimental requirements that enable us to deposit continuous films on the substrates. Other relations could have been obtained if this restriction was not considered, but this would not provide useful information towards our aim to optimize the IJP deposition process.

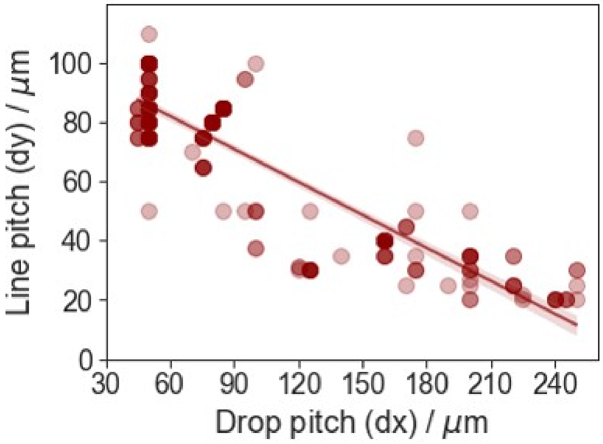

Figure S4. *Scatter plot and linear regression between the Drop pitch (dx) and the Line pitch (dy).*

Figure S5a and **S5b** presents the scatter plots of the *dx* and *dy* with the *NoD* and their linear regressions. We can see that there is a large dispersion in the data, which leads to a weak negative linear coefficient (ρ = -0.39) with the *dx* and a slightly positive (ρ = 0.05) with *dy* (**Figure 2**). **Figure S5c** and **S5d** show the scatter plots for the *AV* and *APL* with the *NoD* and their linear regressions. In this case, the data is highly spread around the most common values, 136 – 155 V for the former and 25 – 26 µs for the latter, which leads to weak and negative linear relations (correlations of -0.11 and -0.22) (**Figure 2**). **Figure S5e** and **S5f** display the scatter plots for the *Amine* and *ADV* with the *NoD* and their linear regressions. The former shows scattering for *Amine* values of 1.14 and 2 %, while it is more evenly distributed in the latter.

Both linear regressions are quite strong and negative with coefficients of -0.47 and -0.62 (**Figure 2**). It must be considered that variables with low correlation coefficients have a large dispersion which implies a weak or not statistically significant linear relationship of these variables and the *NoD.*

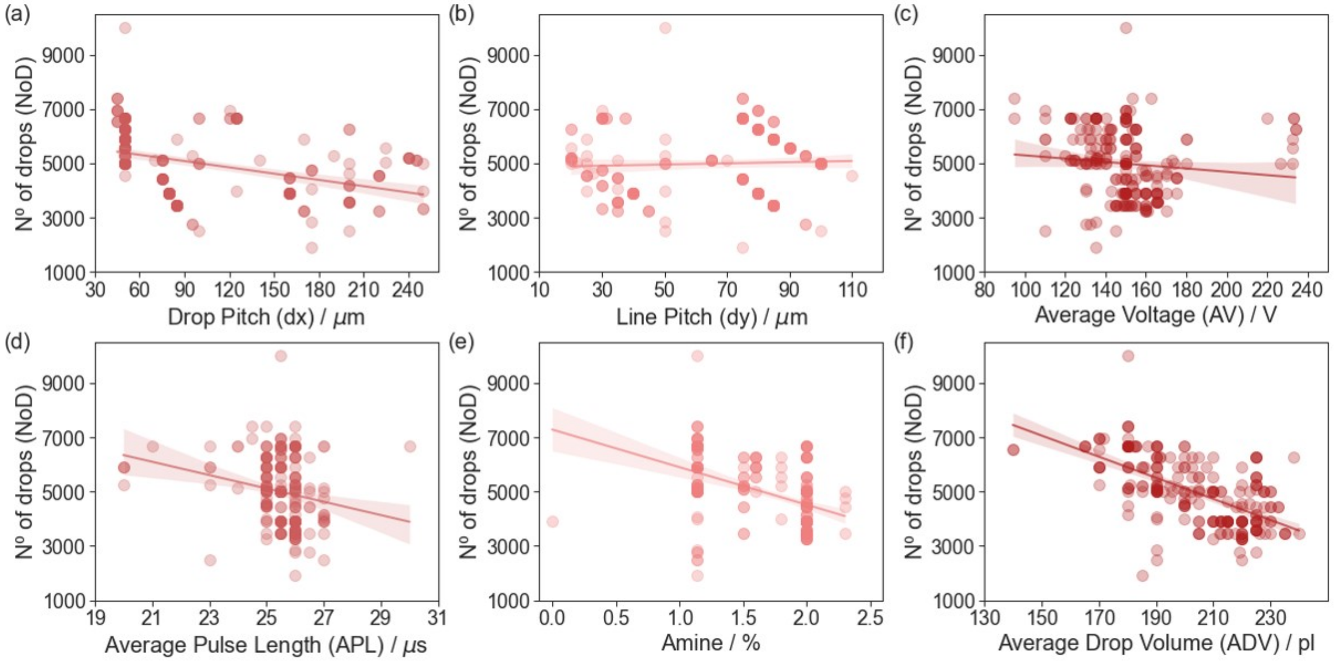

Figure S5. *Scatter plots and linear regressions for the Nº of drops (NoD) with variables defining the deposition: (a) Drop Pitch (dx), (b) Line Pitch (dy), (c) Average Voltage (AV), (d) Average Pulse Length (APL), (e) Amine, and (f) Average Drop Volume (ADV).*

Figure S6a shows the scatter plot of the *NoD* with the *TVD*. We can see that the data is highly confined, indicating a strong and positive linear relation between both variables with a correlation coefficient of 0.91 (**Figure 2**). This may imply a large importance of the *NoD* in defining the *TVD* as we pointed out in the main manuscript. **Figure S6b-S6e** display the scatter plots of the *ADV, AV, APL* and *Amine* with the *NoD*. For the *ADV* and *AV*, the data is scattered along a broad range of values, while the dispersion is centered on the most common values for the *APL* and *Amine*, 25 – 26 µs and 1.14 – 2 %, respectively. All linear regressions show a weak and negative trend with correlation coefficients of -0.08, -0.13, -0.25 and -0.26 (**Figure 2**). **Figure S6f** and **S6g** presents the scatter plots for the *dx* and *dy* with the *TVD* and their

linear regressions. The data is scattered among a broad range of values, although the *dx* has more data points on the range between 50 and 90 µm. The linear regression is rather strong and negative for the *dx*, while it is weak and positive for the *dy*. It must be considered that the large dispersion in the data for variables with low correlation coefficients means that the linear relationship between them and the *TVD* is not statistically significant.

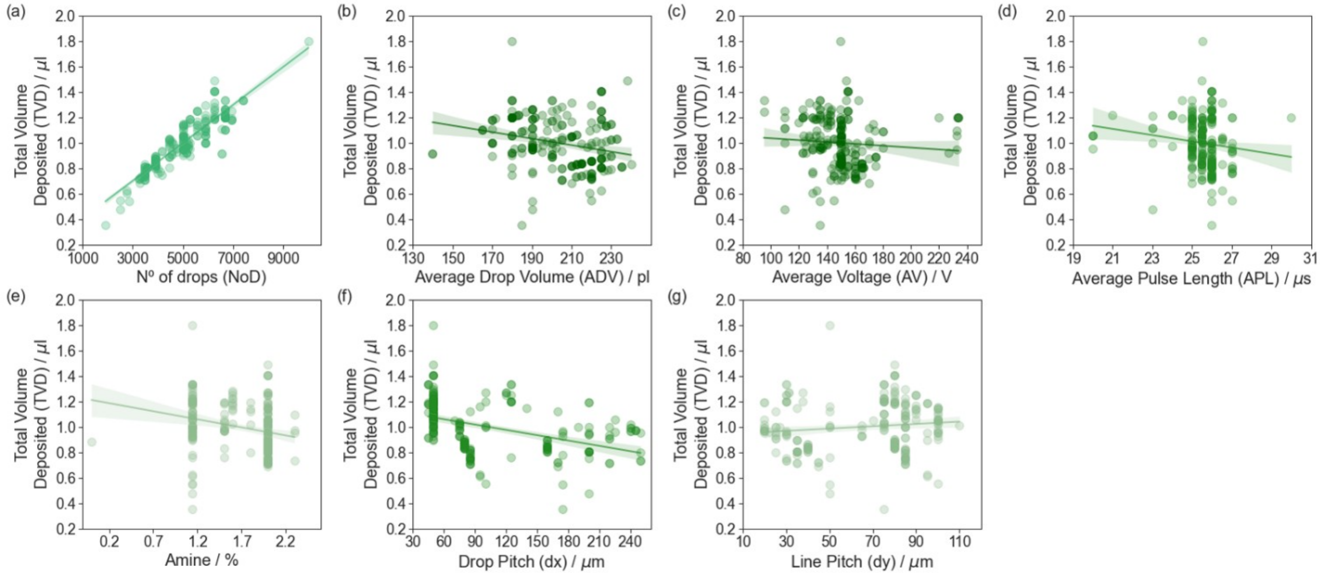

Figure S6. *Scatter plots and linear regressions for the Total Volume Deposited (TVD) with variables defining the deposition: (a) Nº of drops (NoD), (b) Average Drop Volume (ADV), (c) Average Voltage (AV), (d) Average Pulse Length (APL), (e) Amine, (f) Drop Pitch (dx), and (g) Line Pitch (dy).*

S4. Additional information for the model Nº of drops (NoD)

In this section, we present additional information related to the model developed for the variable NoD.

As mentioned in the main manuscript, we have chosen the RF algorithm due to is reliability in preventing overfitting and capturing non-linear interactions. In addition, optimization of RF requires tuning very few hyperparameters. We mainly focused on the *Nº of estimators*, the *Minimum Sample Split* and the *Maximum Depth* and used 5-fold CV to maximize the average R^2 (minimize the average root mean squared error (RMSE)). Then, evaluated these metrics on the validation set (**Figure S7**). First, we tuned

the *Nº of estimators* by exploring a range from 0 to 500 with steps of 10 (**Figure S7a**) and obtained a maximum R^2 (minimum RMSE) around 30 estimators. The *Minimum Sample Split* was explored from 2 to 10 with increments of 1 and using the previous value for the *Nº of estimators*. In this case, the optimum value is found for a *Minimum Sample Split* of 3 (**Figure S7b**). Finally, we optimized the *Maximum Depth* within a range from 2 to 20 and steps of 1, obtaining and optimum value of approximately 10 (**Figure S7c**).

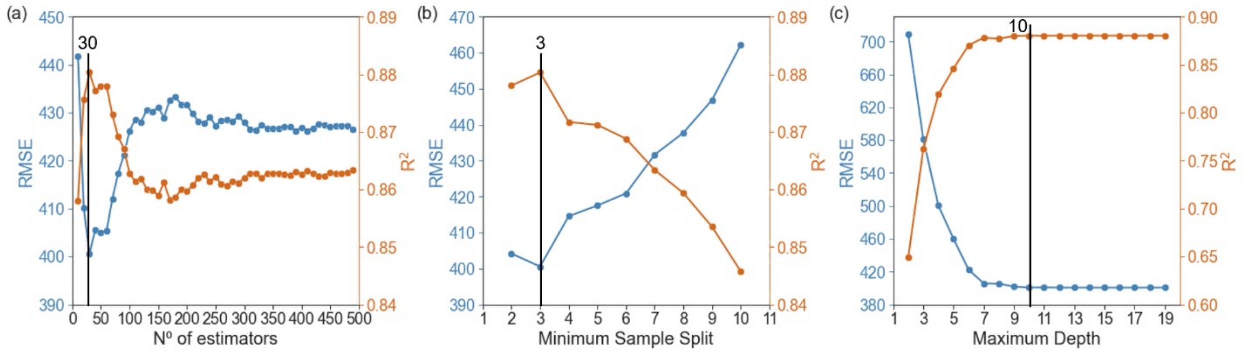

Figure S7. *Evolution of the Root Mean Squared Error (RMSE) and Precision (R^2) with the (a) Nº of estimators (Minimum Sample Split = 3, Maximum Depth =10), (b) Minimum Sample Split (Nº of estimators = 30, Maximum Depth =10), and (c) Maximum Depth (Nº of estimators = 30, Minimum Sample Split = 3).*

Afterwards, we selected the best parameters and retrained the model on the whole train set (including the validation part). **Figure S8** shows these results by also comparing between the RMSE and R^2 for train and test datasets, before and after removing the unimportant variables from the model for the *NoD*. With the full dataset, both metrics are better for the train set as compared to the test one with RMSE values of 175.84 and 288.83, respectively (**Figure S8a**), while the R^2 are 98% and 94% (**Figure S8b**). For the model with only the important variables (reduced dataset), we can see that the train and test RMSE for the *NoD* are slightly lower, 166.56 and 267.31, respectively, whereas the precision remains the same for the train set (98 %) and increases slightly for the test set (95 %). The reason for the improvement between

validation and test scores is likely caused by the averaged metrics over the 5 validation sets that were split from the original train set during cross-validation, while for the test set we have only a single metric.

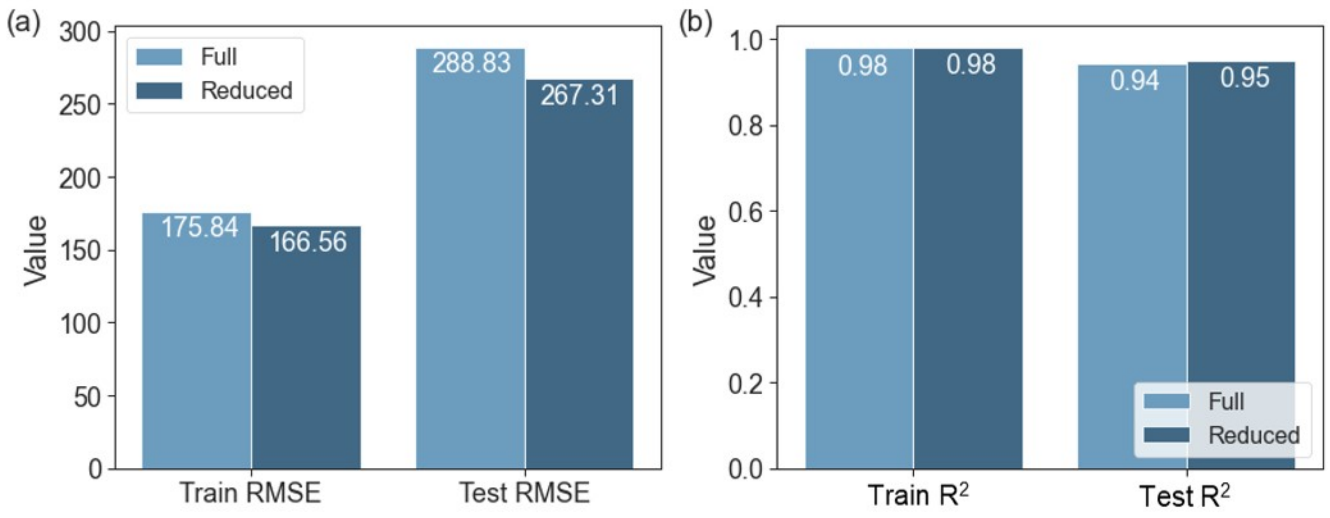

Figure S8. *Comparison between the (a) RMSE and (b) precisions (R^2) for the train and test sets of the Nº of drops (NoD) model employing all or a reduced number of variables based on the importance determined in* ***Figure 4****. The model was obtained with the Random Forest (RF) algorithm and the optimized hyperparameters (Nº of estimators = 30, Minimum Sample Split = 3, Maximum Depth =10).*

Table S2 presents the experimental parameters that would be required to obtain the *Predicted NoD* calculated with the machine learning model built using the reduced dataset with the most important variables selected from feature importance based on the average SHAP values (**Figure 4a** in the main manuscript).

Table S2. *Predicted values of the Nº of drops (NoD) and their relationship with the Average Drop Volume (ADV), Drop Pitch (dx) and Line Pitch (dy).*

**Average Drop Volume (ADV) (pl)**	**Drop Pitch (dx) (µm)**	**Line Pitch (dy) (µm)**	**Predicted Nº of drops (NoD)**
197.5	50	90	5566.45

222.5	50	110	5000
183	50	75	6666.67
210	50	100	5000
190	85	50	5305.56
215	85	85	3460.21
212.5	80	80	3906.25
187	125	30	6655.41
180	45	75	7172.07
190	190	25	4968.19
171.5	45	80	6959.65
202.5	50	80	6250
212.5	85	85	3460.21
215	160	40	3906.25
170	45	80	6959.65
200	220	25	4557.07
180	125	30	6688.27
190	245	20	5095.51
220	80	80	3906.25
180	75	65	5298.71
212.5	80	80	3906.25
225	200	35	3640.57
195	50	80	6208.33
200	125	30	6492.58
225	50	80	6250
180	160	35	5179.79
170	50	95	5285.75
220	75	75	4444.44
220	220	25	4215.56

Figure S9 shows a plot equivalent to that of **Figure 4c** where the contribution of each experimental parameter is evaluated to reach a predicted *NoD* of 3906.3. In this example, a *dx* of 160 µm, a *dy* of 40 µm, and an *ADV* of 215 pl would be required to obtain the predicted value of the *NoD*. In addition, we

see that the *dx* and *ADV* have a negative contribution (reduce) on the final value, while the *dy* increases it.

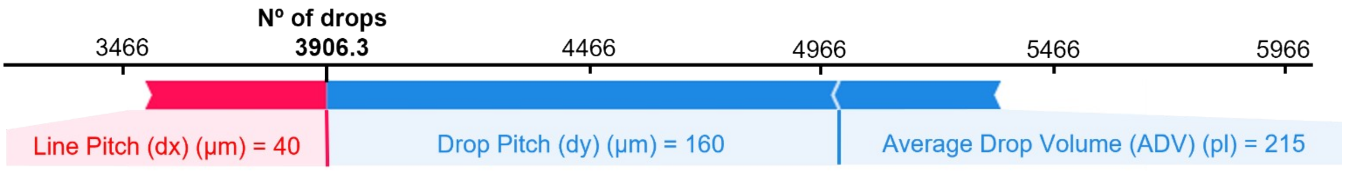

Figure S9. *Prediction of the influence of model parameters on a specific Nº of drops (NoD). The contribution of each feature on the predicted NoD is obtained based on their average values in the train set (<dx> = 100.3 μm, <ADV> = 203.3 pl, and <dy> = 67.6 μm). The red color indicates the features that contribute increasing the predicted value, while blue is used for those that contribute to reduce it. The size of the arrow indicates how strong is the effect of each variable.*[12]

S5. Additional information for the model Total Volume Deposited (TVD)

In this section, we present additional information related to the model developed for the variable *TVD*.

Figure S10 shows the R^2 values for four different machine learning algorithms that were employed to predict the *TVD* variable based on the descriptors. The results are shown for a linear regression, as well as ensemble algorithms (Random Forest, AdaBoost and Gradient Boosting) using the default hyperparameters, and indicate that precision values (R^2) are very similar between them. For the train set, they range from 0.95 to 1, while we have precision values from 0.89 to 0.99 for the validation set. This indicates a high degree of linearity of this variable with the descriptors. Nevertheless, for comparison purposes with the *NoD*, we employed a Random Forest algorithm to model this variable also to extract possible hidden non-linear patterns in the data, as explained in the main manuscript.

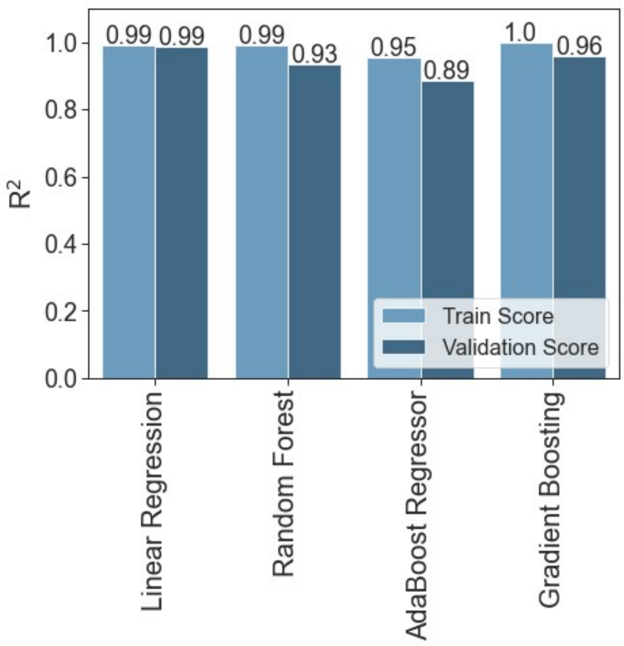

Figure S10. *Precision (R^2) values on the training and validation datasets for the Total Volume Deposited (TVD) models built with linear regression and ensemble methods using the default hyperparameters.*

Like the *NoD* case, we enhanced the model parameters, focusing on the *Nº of estimators*, the *Minimum Sample Split* and the *Maximum Depth*, also carrying out 5-fold cross-validation and maximizing the average R^2 (minimizing the RMSE) by evaluating the model on the test set (**Figure S11**). First, we tuned the *Nº of estimators* by exploring a range from 0 to 500 with steps of 10 (**Figure S11a**) and obtained a maximum R^2 (minimum RMSE) around 120 estimators. The *Minimum Sample Split* was explored from 2 to 10 with increments of 1 and using the previous value for the *Nº of estimators*. In this case, the optimum value is found for a *Minimum Sample Split* of 3 (**Figure S11b**). Finally, we optimized the Maximum Depth within a range from 2 to 20 and steps of 1, obtaining and optimum value of 10 (**Figure S11c**).

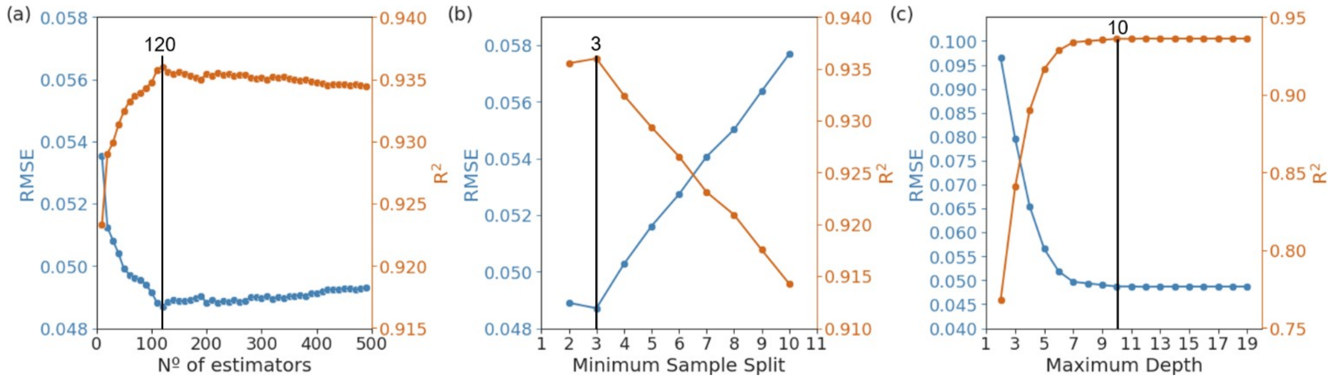

Figure S11. *Evolution of the Root Mean Squared Error (RMSE) and precision (R^2) with the (a) Nº of estimators (Minimum Sample Split = 3, Maximum Depth =10), (b) Minimum Sample Split (Nº of estimators = 120, Maximum Depth =10), and (c) Maximum Depth (Nº of estimators = 120, Minimum Sample Split = 3).*

After retraining the model on the whole train set (including the validation part), we obtained precisions of 0.99 and 0.98 for the train and tests sets, while the RMSE are 0.021 and 0.026 (**Figure S12**). These results illustrate a good match of the model with unseen data that follows the criteria that printed samples must be homogeneous, without liquid movement and no cracks after the pyrolysis.

Figure S12 also shows a comparison between the RMSE and R^2 for train and test datasets, before and after removing the unimportant variables from the model for the *TVD*. With the full dataset, both metrics are slightly better for the train set than for the test one with RMSE values of 0.021 and 0.026, respectively (**Figure S12a**), while the R^2 are 99% and 98% (**Figure S12b**). For the model with only the important variables (reduced dataset), we can see that the train and test RMSE for the *NoD* are slightly lower, 0.018 and 0.021, respectively, whereas the R^2 remains the same for the train set (99 %) and increases slightly for the test set (99 %).

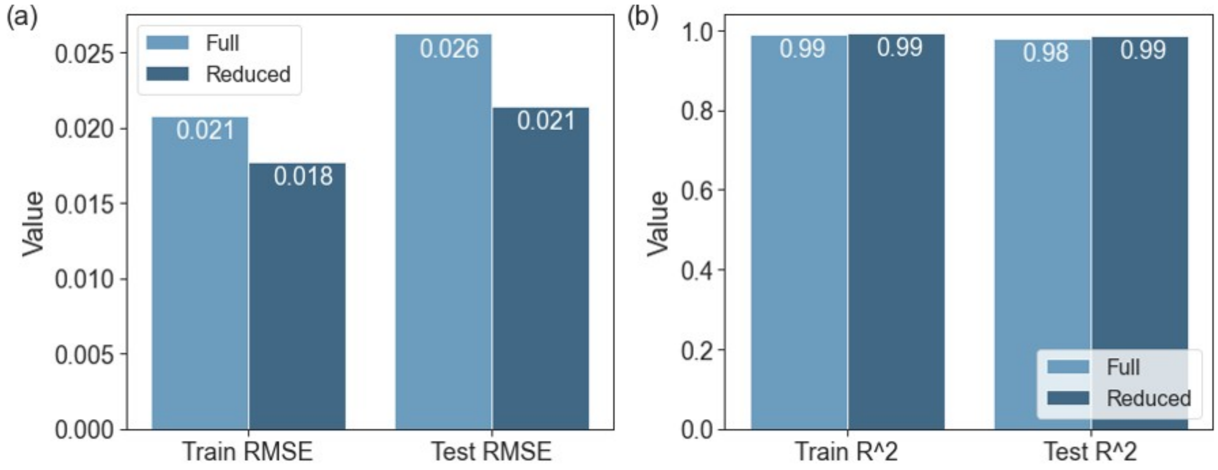

Figure S12. *Comparison between the (a) RMSE and (b) precisions (R^2) for the train and test sets of the Total Volume Deposited (TVD) model employing all or a reduced number of variables based on the importance determined in* ***Figure 6****. The model was obtained with the Random Forest algorithm and the optimized hyperparameters (Nº of estimators = 120, Minimum Sample Split = 3, Maximum Depth =10).*

Table S3 presents the experimental parameters that would be required to obtain the *Predicted TVD* calculated with the machine learning model built using the reduced dataset with the most important variables selected from feature importance based on the average SHAP values (**Figure 6a** in the main manuscript).

Table S3. *Predicted values of the Total Volume Deposited (TVD) and their relationship with the Average Drop Volume (ADV), Drop Pitch (dx), Line Pitch (dy) and Nº of drops (NoD).*

**Average Drop Volume (ADV) (pl)**	**Drop Pitch (dx) (µm)**	**Line Pitch (dy) (µm)**	**Nº of drops (NoD)**	**Predicted Total Volume Deposited (TVD) (µl)**
225	50	95	5263.16	1.18
225	95	95	2770.08	0.6
225	200	30	4166.67	0.92
190	170	25	5882.35	1.12
210	50	100	5000	1.05
220	170	45	3267.97	0.72

212.5	80	80	3906.25	0.83
190	175	35	4081.63	0.77
190	120	31.25	6666.67	1.27
190	50	80	6250	1.2
200	220	25	4545.45	0.91
227.5	75	75	4444.44	1.01
225	80	80	3906.25	0.88
215	80	80	3906.25	0.84
215	160	40	3906.25	0.84
190	100	37.5	6666.67	1.24
225	50	80	6250	1.41
140	45	85	6535.95	0.96
180	50	85	5882.35	1.06
200	160	35	4464.29	0.9
190	50	90	5555.56	1.07
182	50	75	6666.67	1.21
210	50	80	6250	1.31
220	80	80	3906.25	0.86
210	160	40	3906.25	0.82
205	50	80	6250	1.27
230	85	85	3460.21	0.79
225	75	75	4444.44	1
215	160	40	3906.25	0.84

Figure S13 presents a similar plot to that shown in **Figure 6c** which evaluates the influence of each independent variable from the model developed on the reduced dataset (*NoD* and *ADV*) on the predicted value for the *TVD*. In particular, a *NoD* of 4545 (*dx*=220 µm and *dy*=25 µm) and *ADV* of 200 pl would be needed to deposit a total volume of 0.91 µl on a 5 x 5 mm^2 substrate. Both variables contribute to reduce the the final value of the *TVD*.

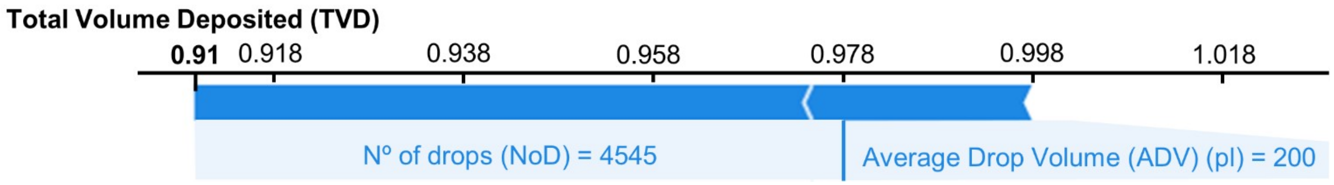

Figure S13. *Prediction of the influence of model parameters on a specific Total Volume Deposited (TVD). The contribution of each feature on the predicted TVD is obtained based on their average values in the train set (<ADV> = 203.3 pl, and <NoD> = 4976.6 drops). A red color would indicate features that contribute increasing the predicted value, while blue is used for those that reduce it. The size of the arrow indicates how strong is the effect of each variable.*[12]

References:

(1) Hastie, T.; Tibshirani, R.; Friedman, J. *The Elements of Statistical Learning*; Springer Series in Statistics; Springer New York: New York, NY, 2009. https://doi.org/10.1007/978-0-387-84858-7.

(2) Sarkar, D.; Bali, R.; Sharma, T. *Practical Machine Learning with Python*; Apress: Berkeley, CA, 2018. https://doi.org/10.1007/978-1-4842-3207-1.

(3) Bonamente, M. *Statistics and Analysis of Scientific Data*; Graduate Texts in Physics; Springer New York: New York, NY, 2017. https://doi.org/10.1007/978-1-4939-6572-4.

(4) Moore, A.; Lee, M. S. Cached Sufficient Statistics for Efficient Machine Learning with Large Datasets. *jair* **1998**, *8*, 67–91. https://doi.org/10.1613/jair.453.

(5) Wang, A. Y.-T.; Murdock, R. J.; Kauwe, S. K.; Oliynyk, A. O.; Gurlo, A.; Brgoch, J.; Persson, K. A.; Sparks, T. D. Machine Learning for Materials Scientists: An Introductory Guide toward Best Practices. *Chem. Mater.* **2020**, *32* (12), 4954–4965. https://doi.org/10.1021/acs.chemmater.0c01907.

(6) Cawley, G. C.; Talbot, N. L. C. On Over-FItting in Model Selection and Subsequent Selection Bias in Performance Evaluation. *Journal of Machine Learning Research* **2010**, *11*, 2079–2107.

(7) Breiman, L. Random Forests. *Machine Learning* **2001**, *45* (1), 5–32. https://doi.org/10.1023/A:1010933404324.

(8) Freund, Y.; Schapire, R. E. A Decision-Theoretic Generalization of On-Line Learning and an Application to Boosting. *Journal of Computer and System Sciences* **1997**, *55* (1), 119–139. https://doi.org/10.1006/jcss.1997.1504.

(9) Friedman, J. H. Greedy Function Approximation: A Gradient Boosting Machine. *Ann. Statist.* **2001**, *29* (5). https://doi.org/10.1214/aos/1013203451.

(10) Bühlmann, P. Bagging, Boosting and Ensemble Methods. In *Handbook of Computational Statistics*; Gentle, J. E., Härdle, W. K., Mori, Y., Eds.; Springer Berlin Heidelberg: Berlin, Heidelberg, 2012; pp 985–1022. https://doi.org/10.1007/978-3-642-21551-3_33.

(11) Pedregosa, F.; Varoquaux, G.; Gramfort, A.; Michel, V.; Thirion, B.; Grisel, O.; Blondel, M.; Prettenhofer, P.; Weiss, R.; Dubourg, V.; Vanderplas, J.; Passos, A.; Cournapeau, D. Scikit-Learn: Machine Learning in Python. *Journal of Machine Learning Research* **2011**, *12*, 2825–2830.

(12) Lundberg, S. M.; Lee, S.-I. A Unified Approach to Interpreting Model Predictions. *31st Conference on Neural Information Processing Systems* **2017**, 4768–4777.

(13) Lundberg, S. M.; Nair, B.; Vavilala, M. S.; Horibe, M.; Eisses, M. J.; Adams, T.; Liston, D. E.; Low, D. K.-W.; Newman, S.-F.; Kim, J.; Lee, S.-I. Explainable Machine-Learning Predictions for the Prevention of Hypoxaemia during Surgery. *Nat Biomed Eng* **2018**, *2* (10), 749–760. https://doi.org/10.1038/s41551-018-0304-0.

(14) Lundberg, S. M.; Erion, G.; Chen, H.; DeGrave, A.; Prutkin, J. M.; Nair, B.; Katz, R.; Himmelfarb, J.; Bansal, N.; Lee, S.-I. From Local Explanations to Global Understanding with Explainable AI for Trees. *Nat Mach Intell* **2020**, *2* (1), 56–67. https://doi.org/10.1038/s42256-019-0138-9.

(15) Lundberg, S. M.; Erion, G. G.; Lee, S.-I. Consistent Individualized Feature Attribution for Tree Ensembles. *arXiv:1802.03888 [cs, stat]* **2019**.

(16) Queraltó, A.; Banchewski, J.; Pacheco, A.; Gupta, K.; Saltarelli, L.; Garcia, D.; Alcalde, N.; Mocuta, C.; Ricart, S.; Pino, F.; Obradors, X.; Puig, T. Combinatorial Screening of Cuprate Superconductors by Drop-On-Demand Inkjet Printing. *ACS Appl. Mater. Interfaces* **2021**, *13* (7), 9101–9112. https://doi.org/10.1021/acsami.0c18014.
